# Syntheses, Structures, and Corrosion Inhibition of Various Alkali Metal Carboxylate Complexes

**DOI:** 10.3390/molecules28145515

**Published:** 2023-07-19

**Authors:** Vidushi P. Vithana, Zhifang Guo, Glen B. Deacon, Peter C. Junk

**Affiliations:** 1 College of Science & Engineering, James Cook University, Townsville, QLD 4811, Australia; vidushi.vithana@my.jcu.edu.au (V.P.V.); zhifang.guo@jcu.edu.au (Z.G.); 2 School of Chemistry, Monash University, Clayton, VIC 3800, Australia; glen.deacon@monash.edu

**Keywords:** alkali metal, carboxylate complexes, 3-thiophenecarboxylate, 2-methyl-3-furoate, 3-furoate, 4-hydroxycinnamate, 4-hydroxybenzoate, structures, corrosion inhibitors

## Abstract

Complexes of the alkali metals Li-Cs with 3-thiophenecarboxylate (3tpc), 2-methyl-3-furoate (2m3fur), 3-furoate (3fur), 4-hydroxycinnamate (4hocin), and 4-hydroxybenzoate (4hob) ions were prepared via neutralisation reactions, and the structures of [Li_2_(3tpc)_2_]_n_ (**1Li**); [K_2_(3tpc)_2_]_n_ (**1K**); [Rb(3tpc)(H_2_O)]_n_ (**1Rb**); [Cs{H(3tpc)_2_}]_n_ (**1Cs**); [Li_2_(2m3fur)_2_(H_2_O)_3_] (**2L**i); [K_2_(2m3fur)_2_(H_2_O)]_n_ (**2K**); [Li(3fur)]_n_(**3L**i); [K(4hocin](H_2_O)_3_]_n_ (**4K**); [Rb{H(4hocin)_2_}]_n_.nH_2_O (**4Rb**); [Cs(4hocin)(H_2_O)]_n_ (**4Cs**); [Li(4hob)]_n_ (**5Li**); [K(4hob)(H_2_O)_3_]_n_ (**5K**); [Rb(4hob)(H_2_O)]_n_ (**5Rb**); and [Cs(4hob)(H_2_O)]_n_ (**5Cs**) were determined via X-ray crystallography. Bulk products were also characterised via XPD, IR, and TGA measurements. No sodium derivatives could be obtained as crystallographically suitable single crystals. All were obtained as coordination polymers with a wide variety of carboxylate-binding modes, except for dinuclear **2Li**. Under conditions that normally gave coordinated carboxylate ions, the ligation of hydrogen dicarboxylate ions was observed in **1Cs** and **4Rb**, with short H-bonds and short O…O distances associated with the acidic hydrogen. The alkali-metal carboxylates showed corrosion inhibitor properties inferior to those of the corresponding rare-earth carboxylates.

## 1. Introduction

The addition of chemical corrosion inhibitors is a highly efficient approach in managing the multi-billion-dollar cost associated with corrosion in metallic structures, particularly in steel infrastructure [1]. Corrosion protection through the use of metal–organic corrosion inhibitors has emerged as a substitute for highly toxic and environmentally hazardous Cr(VI) compounds [2]. The use of metal–organic corrosion inhibitors is associated with the formation of protective layers on metals [2]. In recent years, rare-earth carboxylate derivatives have gained significant attention as effective corrosion inhibitors [3,4,5,6,7]. Our previous work demonstrated the usefulness of various rare-earth (RE) carboxylates as corrosion inhibitors [2,4,6,7,8,9,10]. Specifically, we found cerium salicylate [4], lanthanum 4-hydroxycinnamate [11], yttrium 3-(4′-methylbenzoyl)-propanoate [12], yttrium 3-furoate [13], and rare-earth 3-thiophenecarboxylates [14] and 4-hydroxybenzoates [15] to be promising. The numerous possible coordination modes of carboxylate ligands [16,17] have also made this area fruitful for coordination and structural chemistry.

Rare-earth carboxylates are normally prepared via metathesis reactions between sodium carboxylates, often prepared in situ, and rare-earth salts. Because it has been claimed that the use of alkali-metal salts of carboxylic acids prevent corrosion in water treatment facilities [10], the sodium salts are usually tested for comparison with the rare-earth carboxylates, and inevitably perform much worse [3,4,10,13,18]. Moreover, preliminary immersion studies performed by Wormwell and Mercer [19] demonstrated that lithium and potassium benzoates also exhibit inhibitory properties against mild-steel corrosion. However, relatively high concentrations of alkali-metal carboxylates are needed to achieve a good inhibitory effect, where they often act as anodic inhibitors [11,20]. 

**Chart 1:**
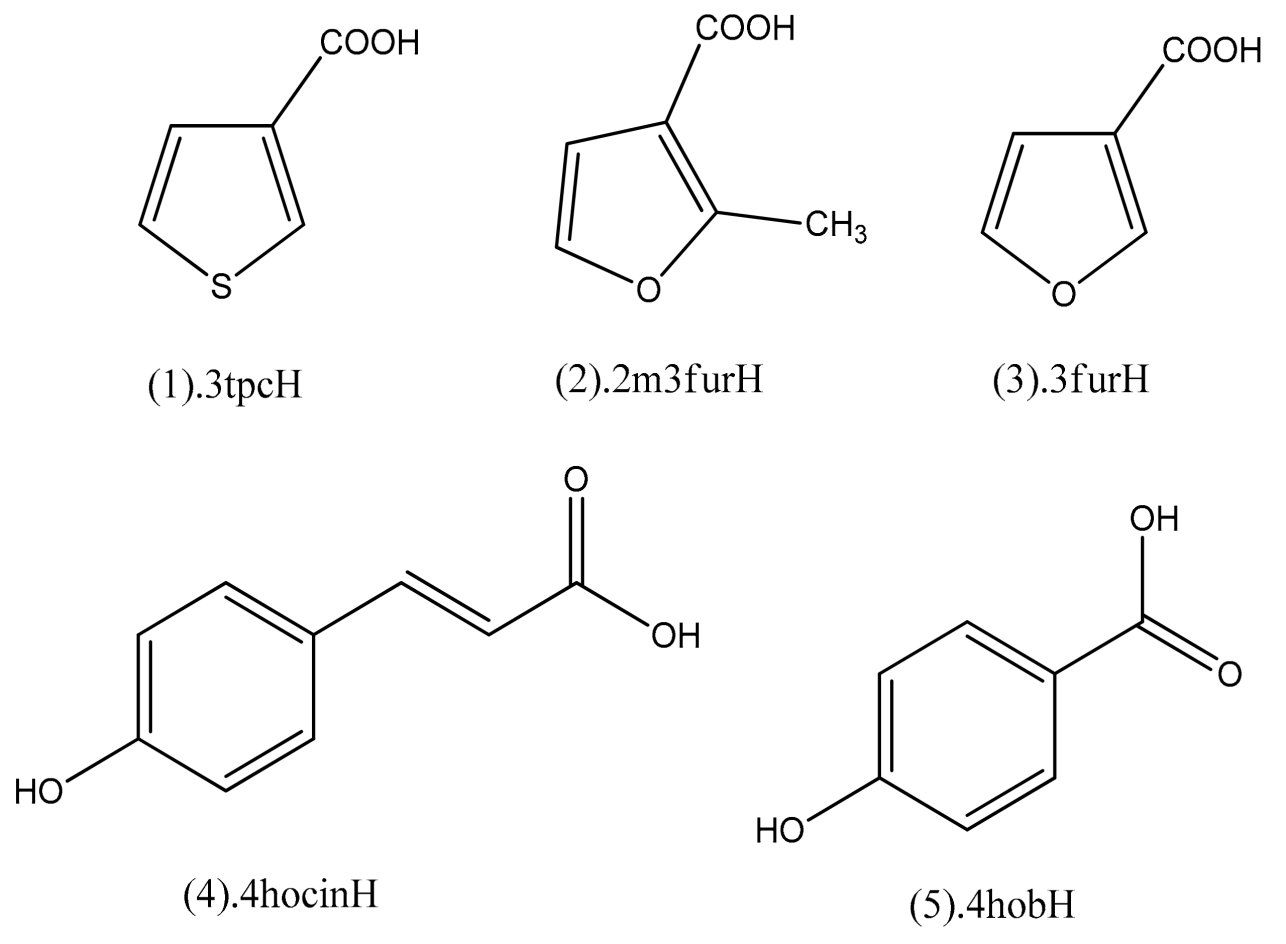
Structures of (**1**) 3-thiophenecarboxylic acid (3tpcH), (**2**) 2-methyl-3-furoic acid (2m3furH), (**3**) 3-furoic acid (3furH), (**4**) 4-hydroxycinnamic acid (4hocinH), and (**5**) 4-hydroxybenzoic acid (4hobH).

Mercer et al. examined a series of benzoate compounds as corrosion inhibitors and concluded that the introduction of various substituents (-CH_3_, -NO_2_, -NH_2_, -OH, -CH=CH-) improves the inhibition performance of benzoates [10,21]. Whilst we have regularly compared the performances of sodium salts with those of their rare-earth counterparts, we have not examined Li, K, Rb, and Cs analogues. When we examined the CCDC structural database, there were no structures of the alkali-metal derivatives of the carboxylic acids (1)–(4) (Chart 1) used in our rare-earth inhibitors [22]. In the case of 4-hydroxybenzoic acid (4hobH, (5) Chart 1), only the structure of the sodium salt has been determined and from X-ray powder data [23], whilst the structures of potassium and rubidium hydrogen di-4-hydroxybenzoates [M[H(4hob)_2_]] (M = K, Rb) [24,25], but not M(4hob) derivatives, are known. There is also a lack of vertical (Li-Cs) studies on structures of rare-earth carboxylates [26,27]. In view of this lack of detailed structural studies, we investigated the synt-heses, structures, and corrosion inhibitor properties of the alkali-metal complexes of these carboxylate ions.

The alkali metals are highly prevalent in the Earth’s crust, and two out of the eight most common elements found in the crust belong to the Group 1 elements. The importance of alkali metals in biology, and specifically Na^+^ and K^+^ ions, cannot be overstated [27]. Approximately one-third of all proteins rely on the presence of metal ions for their structural stability and proper functioning [26]. The extensive utilisation of alkali-metal compounds in diverse technical applications emphasises their immense importance in both chemistry and biology. These applications range from the utilisation of lithium compounds in batteries, organic synthesis, and pharmaceuticals, to the use of sodium in silicate-based glasses, and the use of potassium ions to enhance the scratch resistance of glass surfaces [27].

## 2. Results

### 2.1. Synthesis and Characterisation

**Scheme 1:**
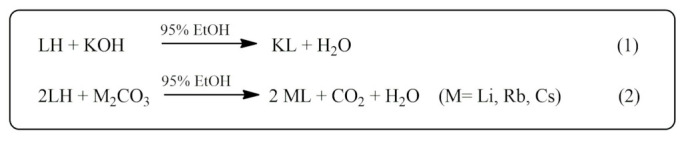
The synthesis of alkali-metal carboxylate complexes.

By varying the preparative conditions, the syntheses of alkali-metal (Li–Cs) complexes of 3-thiophenecarboxylate (3tpc), 2-methyl-3-furoate (2m3fur), 3-furoate (3fur), 4-hydroxycinnamate (4hocin), and 4-hydroxybenzoate (4hob) were carried out via neutralisation reactions in an aqueous solution (Scheme 1). Only the complexes that were characterised by single-crystal XRD are reported in this paper. When complexed with sodium, none of the five ligands formed crystalline products suitable for X-ray crystallography. The bulk samples of sodium complexes were characterised by powder XRD, IR, and TGA in the ESI^†^, and the results suggest that they have dissimilar structural profiles compared to the other alkali-metal carboxylate complexes in the same group. Therefore, further characterisations were not carried out. In the case of **1Cs** and **4Rb**, acid salts [M{H(L)_2_}] (M = Rb, Cs) were obtained, despite intentionally maintaining a 1:1 M:LH reaction stoichiometry (M = alkali metal; L = ligand) to prevent the incorporation of the carboxylic acid into the product (see Introduction for previous similar issues). Among the structures, **1Cs**, **2K**, and **5Li** exhibited slight hygroscopicity. This was evident from the thermal analysis of **1Cs** and **2K**, as well as the microanalysis of **5Li**, which showed values consistent with water absorption.

The compositions of the [M(3tpc)]_n_ (M = Li, K, Rb, and Cs) complexes in the **1M** series were determined through X-ray crystallography, elemental analysis, and thermogravimetric analysis (TGA). The elemental analysis agreed with the single-crystal compositions for all four compounds. The TGA results of **1Li**, **1K**, and **1Rb** (Figure S1) were consistent with the compositions from the single-crystal data. However, the TGA of **1Cs** showed a loss of 0.5 water molecules in a single step below 150 °C. Nonetheless, the powder XRD analyses of all four compounds showed close similarities to the patterns generated from the single-crystal data (Figure S2).

In the [M(2m3fur)]_n_ (M = Li, K) **2M** series, the TGA and elemental analysis of compound **2Li** aligns with the composition determined crystallographically as [Li_2_(2m3fur)_2_(H_2_O)_3_]. Although the elemental analysis of compound **2K** matches the composition of [K_2_(2m3fur)_2_(H_2_O)]_n_ of the crystal structure, the TGA of the bulk precipitate indicated the presence of water of crystallisation (Figure S4). The weight loss below 200 °C indicated the evolution of nearly two water molecules. This suggests that the compound may be hygroscopic after undergoing drying to a constant weight over silica gel. Nonetheless, the powder XRD analyses revealed similar d-spacings to those generated from the single-crystal data for both **2Li** and **2K** (Figure S5).

The elemental analysis of lithium-3-furoate (**3Li**) aligns with the calculated percentage composition for [Li(3fur)]_n_ (**3Li**), and the thermogravimetric analysis (Figure S7) and IR analysis (Figure S9) of the sample confirm the absence of any water. Additionally, the powder diffractogram obtained from the bulk precipitate matches the calculated diffractogram based on the single-crystal data (Figure S8).

All three compounds in the M(4hocin) **4M** series (M = K, Rb, and Cs) have different compositions. The TGA of compound **4K** yielded results consistent with its crystal composition, revealing the loss of three water molecules in a single step below 100 °C (Figure S10), but the elemental analysis of it indicated a loss of 1.5 ligated water molecules due to the drying of the samples over silica gel. Nonetheless, the powder diffractogram of the bulk sample matches the one generated from the crystal data (Figure S11).

In the case of compound **4Rb**, the structure was determined as [Rb{H(4hocin)_2_}]_n_.nH_2_O, but the analysis of the bulk product through microanalysis and TGA suggests that 0.75 4hocinH had been lost from the crystal composition. Unlike in **4K**, the experimental powder diffractogram of the bulk sample **4Rb** exhibited a noticeable difference when compared to the diffractogram generated from the crystal data attributed to the loss of 4hocinH. Upon drying the crystals of compound **4Rb** over silica gel, they seemed to lose their crystalline form and appeared more amorphous.

By comparison, the caesium analogue prepared under similar conditions was determined via crystallography to be [Cs(4hocin)(H_2_O)]_n_, which was consistent with the elemental analysis and TGA results. The powder XRD analysis of the bulk sample of **4Cs** closely resembles the powder diffractogram generated from the crystal data, but a few minor discrepancies can be observed (Figure S11).

The TGA thermal data of **5Li** (Figure S13) indicated that it is an anhydrous compound, which aligns with the X-ray composition of [Li(4hob)]_n_. However, the elemental analysis of the complex indicated the presence of some water, suggesting it is slightly hygroscopic. The powder diffractogram closely resembles the generated powder diffractogram obtained from the crystal data (Figure S14), indicating that the structural integrity is maintained.

The TGA of **5K** revealed the loss of three water molecules in two steps, occurring below 75 °C and below 150 °C. However, the elemental analyses of the bulk sample yielded a molecular composition of [K(4hob)(H_2_O)]_n_, as opposed to the composition derived from the crystal data (namely, [K(4hob)(H_2_O)_3_]_n_), indicating efflorescence. Additionally, the experimentally obtained powder diffractogram differs from the powder diffractogram generated based on its crystal data, consistent with the water loss (Figure S14).

The two complexes **5Rb** and **5Cs** have the general formulae [M(4hob)(H_2_O)]_n_ (M = Rb or Cs) from elemental analyses (C, H). The TGA results for **5Rb** are in support, but the bulk [Cs(4hob)(H_2_O)]_n_ (**5Cs**) lost a small fraction of coordinated water during drying prior to the TGA. The powder diffractograms of both compounds exhibited some similarities and displayed peaks that matched the generated diffractograms based on their crystal data.

The IR spectra of all the complexes displayed peaks corresponding to carboxylate COO^-^ stretching bands in the range of 1560–1360 cm^−1^ (Figure S15). These peaks included both asymmetric stretching bands (from 1509 to 1556 cm^−1^) and symmetric stretching bands (1361–1399 cm^−1^). Additionally, the presence of a broad band in the range of 3200–3550 cm^−1^ indicates the presence of water molecules and hydrogen-bonded alcohol O–H stretching (3400–3200 cm^−1^) in the compounds. Having absorption bands in the region of 1617–1682 cm^−1^ in **1Cs** confirms the presence of an [H(3tpc)_2_] ligand with the C(O)OHO(O)C group (Figure S3). The IR spectra for all three compounds in the **4M** series have bands of moderate intensity in the range of 1633–1636 cm^−1^, which corresponds to the stretching of the propenyl C=C double bond. The consistent υ(C=C) frequencies indicate that the propenyl group is not engaged in coordination [28]. The carbonyl absorption band of [H(4hocin)_2_] in the range of 1617–1682 cm^−1^ is not clear in the spectrum of compound **4Rb** (Figure S12). Table 1 presents a summary of the significant absorption bands observed in the IR spectra of all 14 compounds.

### 2.2. X-ray Crystal Structures

**Figure 1:**
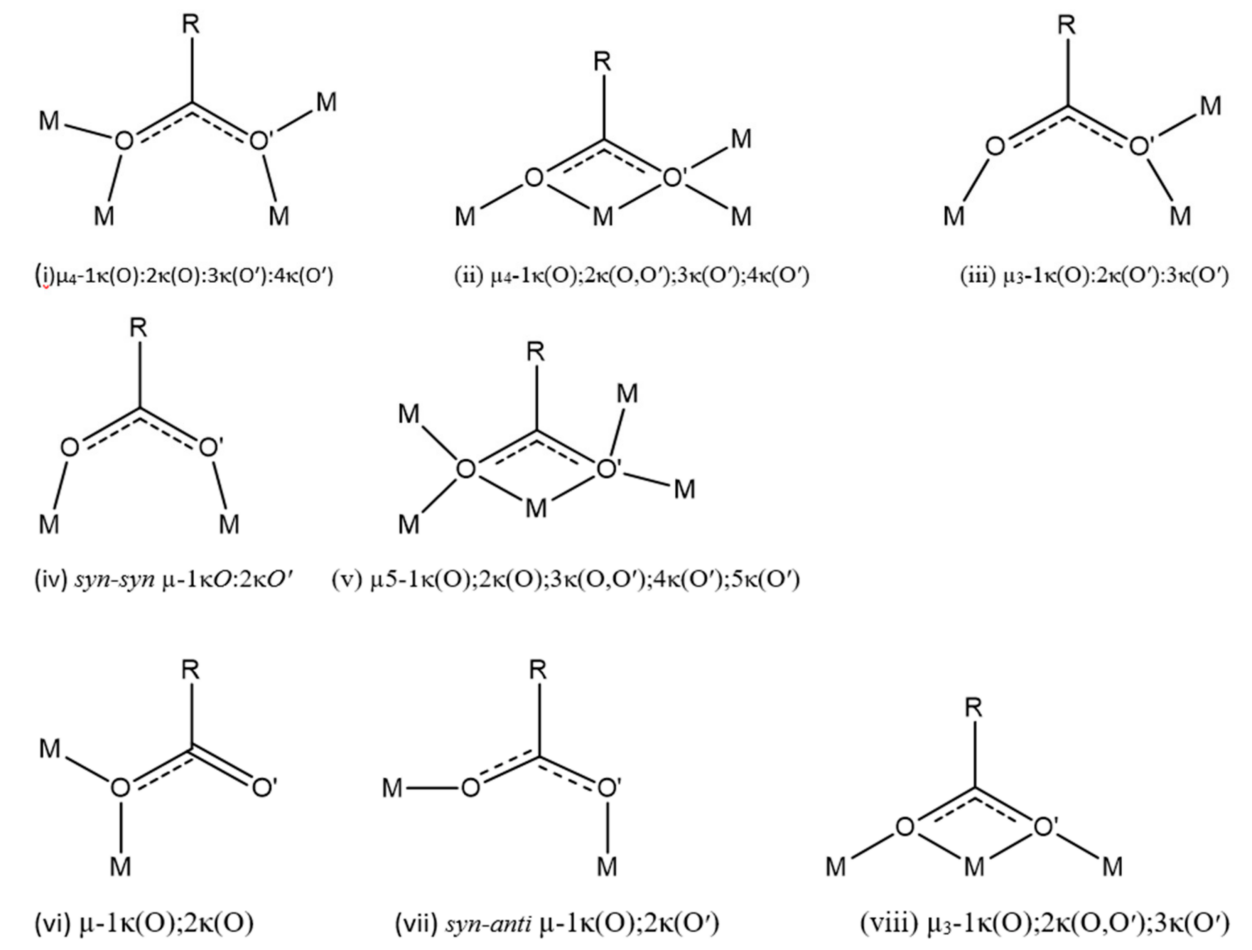
Different coordination modes observed for 3tpc, 2m3fur, 3fur, 4hocin, and 4hob ions.

The carboxylate-binding modes observed in the complexes are shown in Figure 1, and a summary of the features of the structures is given in Table 2. The increase in the average metal–oxygen bond distances, as well as the increase in the coordination number, can be observed as the ionic radius increases from Li to Cs [29] within each series. However, **4K** stands out as an outlier in this trend (Table 2). The ESI^†^ provides detailed information regarding the bond distances and angles.

#### 2.2.1. M(3tpc) Complexes (1M = 1Li, 1K, 1Rb, 1Cs)

**Figure 2:**
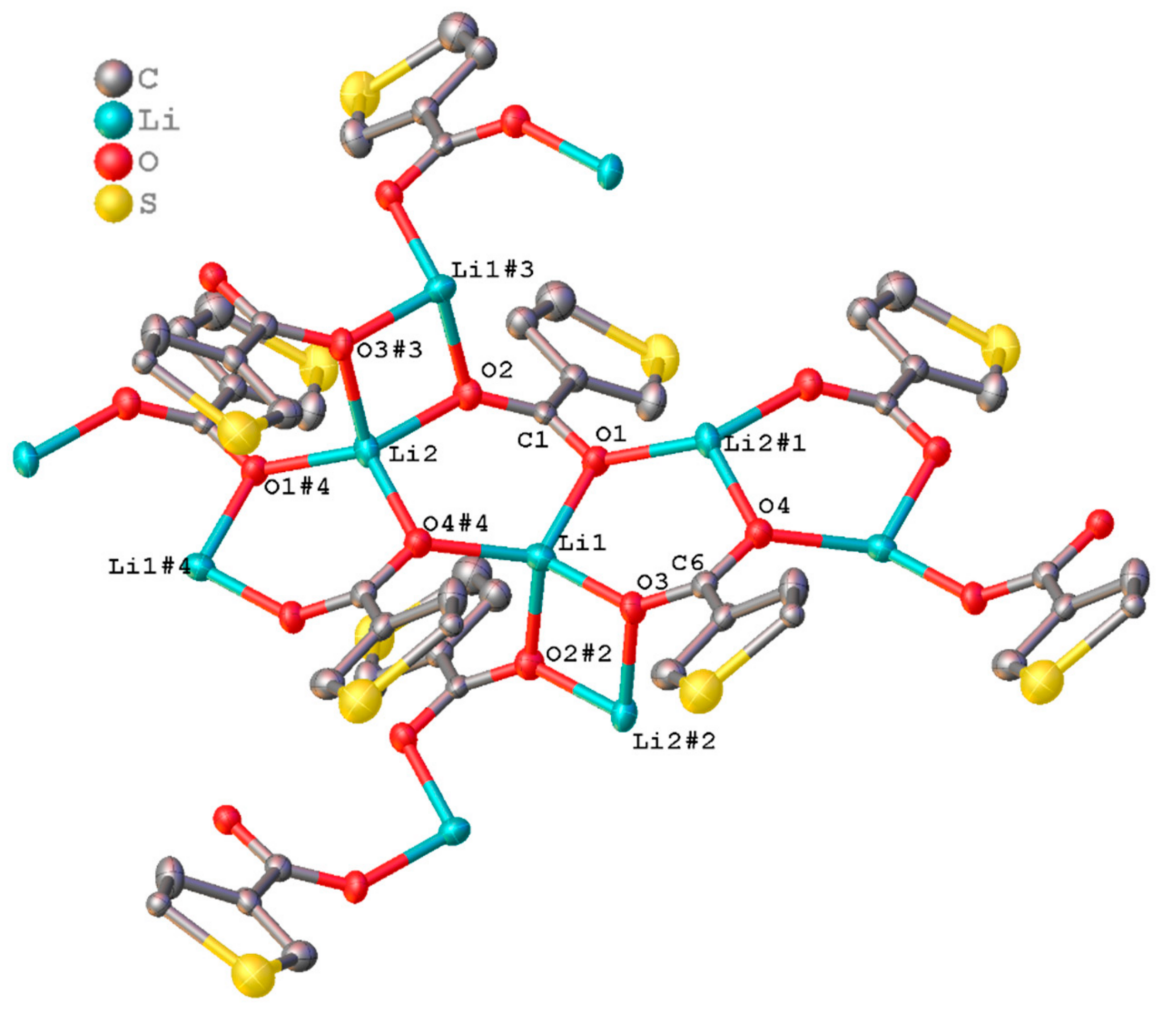
The asymmetric unit of [Li_2_(3tpc)_2_]_n_ **1Li** expanded to show the Li coordination spheres. Symmetry code: ^#1^ + X,1 + Y, + Z; ^#2^ 1/2 + X,1/2-Y, + Z; ^#3^ -1/2 + X,1/2-Y, + Z; ^#4^ + X,-1 + Y, + Z.

[Li_2_(3tpc)_2_]_n_ (**1Li**) crystallises in the *Pna*2_1_ space group as a two-dimensional network. There are two tetracoordinated metal atoms, each Li1 and Li2, with a tetrahedral geometry. Each lithium atom is coordinated by four µ_4_-1κ(O):2κ(O):3κ(O′):4κ(O′) carboxylate oxygen atoms: Li1: O1, 3, 2#2, and 4#4, and Li2: O2, 3#3, 1#4, and 4#4. Li1 and Li2 are linked together in alternating six-member rings, forming a 1D chain produced by the bridging of five µ_4_-1κ(O):2κ(O):3κ(O′):4κ(O′) carboxylate oxygen atoms (O1, O2, O4#4, O3, O4). The Li1…Li2 separation is 3.146(11) Å, and the Li1#4…Li2 separation is 3.116(10) Å. The five oxygen atoms in the six-membered rings have distinct coordination angles: Li1–O1–C1 (139.3(4)°); Li2–O2–C1 (115.9(3)°); Li1–O4#4–Li2 (105.6(4)°); Li1-O3-C6 (138.7(4)°); and Li2#1-O4-C6 (115.6(3)°) (Figure 2).

**Figure 3:**
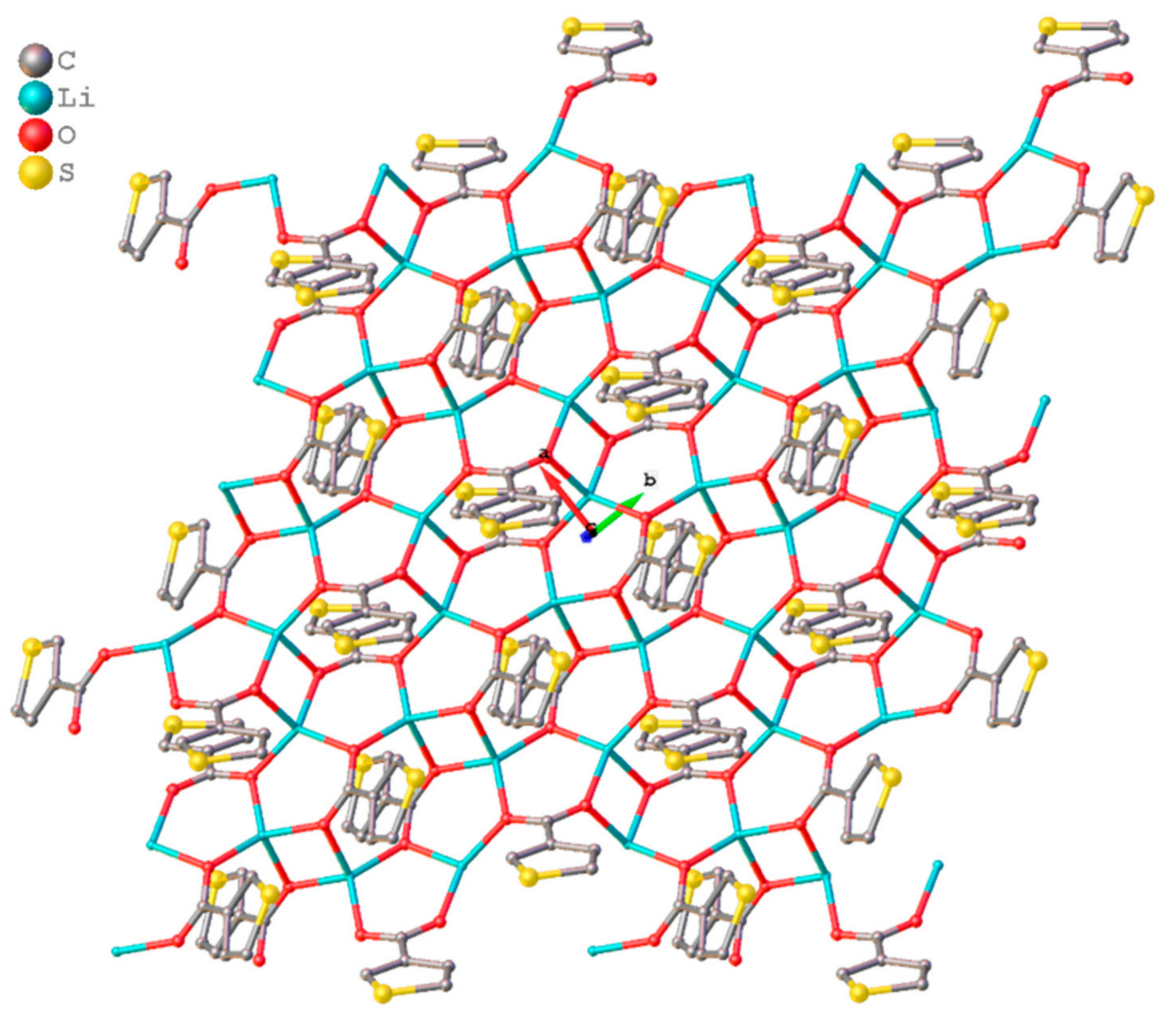
Projection along the c-axis showing the crystal packing of **1Li** into a **2D** network.

The Li2#1-Li1-Li2-Li1#4 chain of the complex crosslinks with neighbouring chains through bridging 3tpc ligands, forming a 2D network. Li2 is connected to the Li1#3 metal atom of the neighbouring chain through oxygen atoms (O2 and O3#3), while Li1 is bound to Li2#2 through bridging oxygen atoms (O3 and O2#2). In both cases, there is a separation of 2.712(9) Å, forming four-membered rings that are perpendicular to each other (Figure 3). The longest metal–oxygen bond in the structure is between Li2 and O3#3, while the shortest bond is between Li1 and O3.

**Figure 4:**
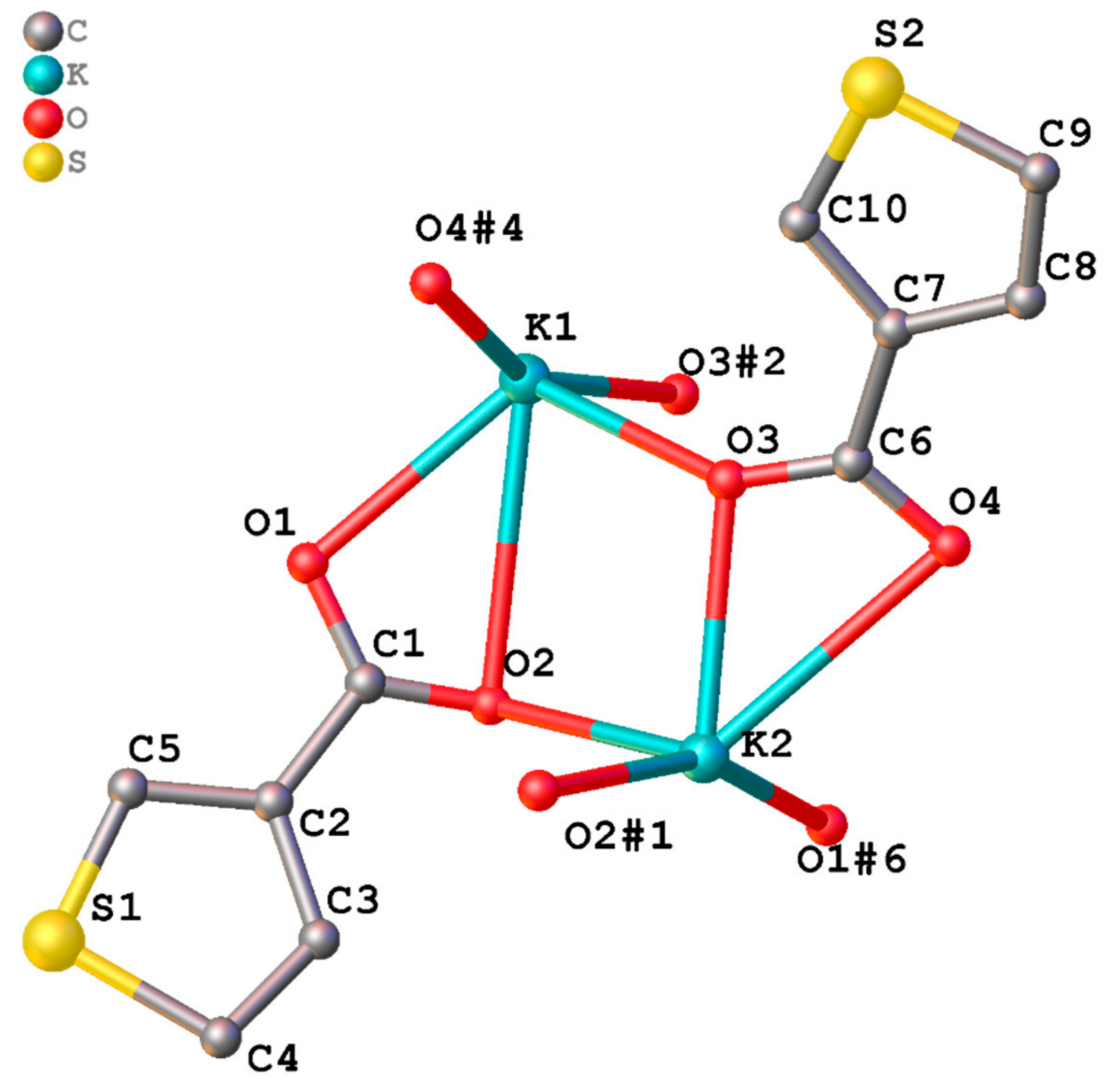
The asymmetric unit of [K_2_(3tpc)_2_]_n_ **1K** expanded to show the K coordination. Symmetry code: ^#1^ + X,-1 + Y, + Z; ^#2^ + X,1 + Y, + Z; ^#4^1/2 + X,-Y, + Z; ^#6^-1/2 + X,1-Y, + Z.

The complex [K_2_(3tpc)_2_]_n_ (**1K**) crystallises in the orthorhombic *Pca*2_1_ space group as a 2D polymer. The asymmetric unit is composed of two potassium atoms, K1 and K2, both of which are five-coordinated. The geometry around the five-coordinated K is distorted from any regular polyhedron due to the arrangements of three bridging ligands and one chelating ligand atom around the metal atoms. K1 and K2 are separated by a distance of 4.208(2) Å and are linked by two µ_4_-1κ(O);2κ(O,O′);3κ(O′);4κ(O′) ligands (O1,2 and O3,4) (Figure 4).

**Figure 5:**
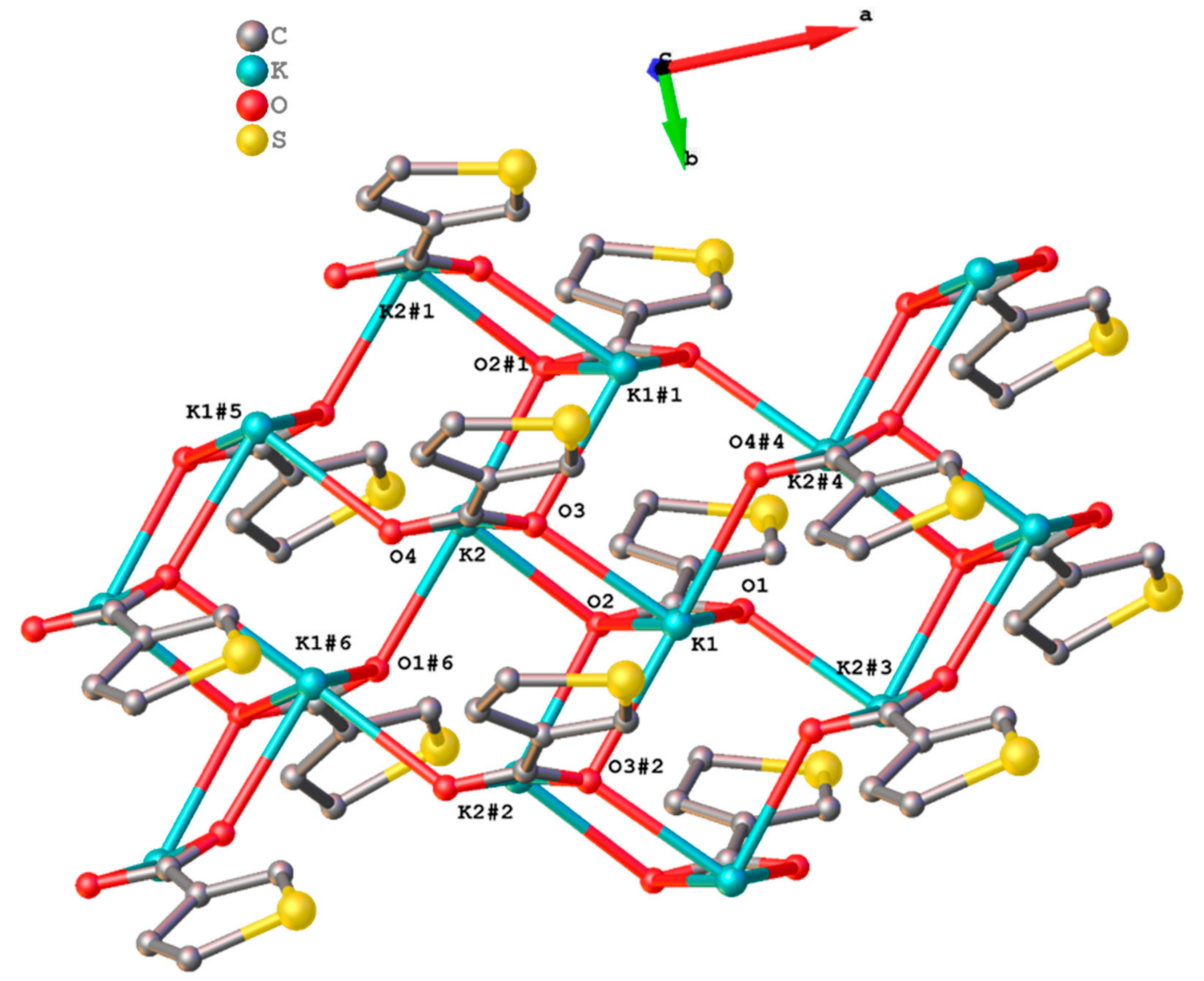
A c-axis projection of the crystal packing of **1K**. Symmetry code: ^#1^ + X,-1 + Y, + Z; ^#2^ + X,1 + Y, + Z; ^#3^ 1/2 + X,1-Y, + Z; ^#4^ 1/2 + X,-Y, + Z; ^#5^ -1/2 + X,-Y, + Z; ^#6^ -1/2 + X,1-Y, + Z.

The former pair are chelated to K1 and bridge to K2, K2#2, and K2#3, while the latter pair are chelated to K2 and bridge to K1, K1#1, and K1#5 through carboxylate oxygen bonds (Figure 5). The dinuclear units are connected into a 1D chain by the *chelating, bridging* ligands between K2 and K1#6 (through O1#6), as well as between K1 and K2#4 (through O4#4), separated by distances of 4.058 (2) Å and 4.108 (2) Å, respectively. The 1D polymeric chain extends into a 2D network via the coordination of the bridging ligand (through O3 and O2#1) to the metal atoms in neighbouring chains with an inter-chain distance (K1…K1#1 and K2…K2#1) of 3.9751 (1) Å. The longest metal–oxygen bond is 3.155(7) Å between K1 and O2, involving a chelating oxygen atom. The shortest metal–oxygen bond is 2.635(6) Å between K2 and O1#6, arising from a bridging oxygen atom.

**Figure 6:**
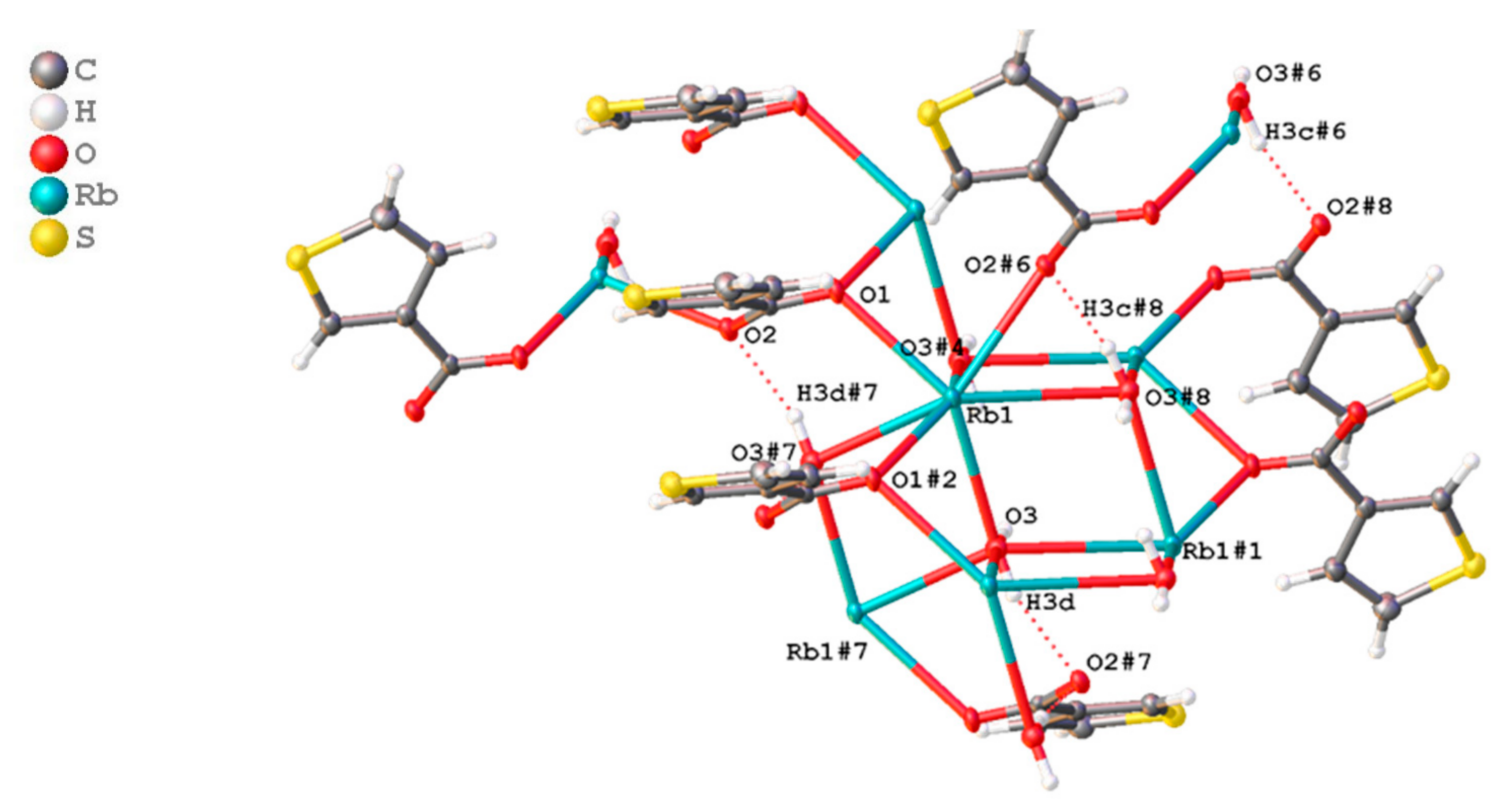
The immediate coordination environment around Rb metal atom in [Rb(3tpc)(H_2_O)]_n_ **1Rb**. Symmetry code: ^#1^ 1-X,1/2 + Y,3/2-Z; ^#2^ + X,1 + Y, + Z; ^#4^ + X,-1 + Y, + Z; ^#6^ + X,1/2-Y,1/2 + Z; ^#7^ 1-X,2-Y,1-Z; ^#8^ 1-X,-1/2 + Y,3/2-Z.

The complex [Rb(3tpc)(H_2_O)]_n_ (**1Rb**) consists of mononuclear units and crystallises in the monoclinic *P*2_1_*/c* space group (Figure 6). The seven-coordinate rubidium metal centres have a distorted capped trigonal prismatic geometry. Three of the seven coordinating oxygen atoms are provided by carboxylate ligands, while the remaining four oxygen atoms are from water. In the structure, only one carboxylate binding mode is observed, with three µ_3_-1κ(O):2κ(O′):3κ(O′) carboxylates coordinating through the oxygen atoms O1, O1#2, and O2#6. The four oxygen atoms in the ligated water molecules are O3, O3#4, O3#7, and O3#8, with each water oxygen bound to four rubidium atoms. Specifically, O3 binds to Rb1, Rb1#1, Rb1#2, and Rb1#7. µ_4_-aqua ligands are rare but known [30,31,32,33]. Compound **1Rb** forms a one-dimensional (1D) polymer via bridged Rb1#7…Rb1…Rb1#1 chains. The separation between the Rb metal centres is Rb1…Rb1#1 (5.1213(12) Å) and Rb1#7…Rb1 (4.8220(13) Å), respectively, with Rb1#7…Rb1…Rb1#1 at an angle of 51.910(19)°.

**Figure 7:**
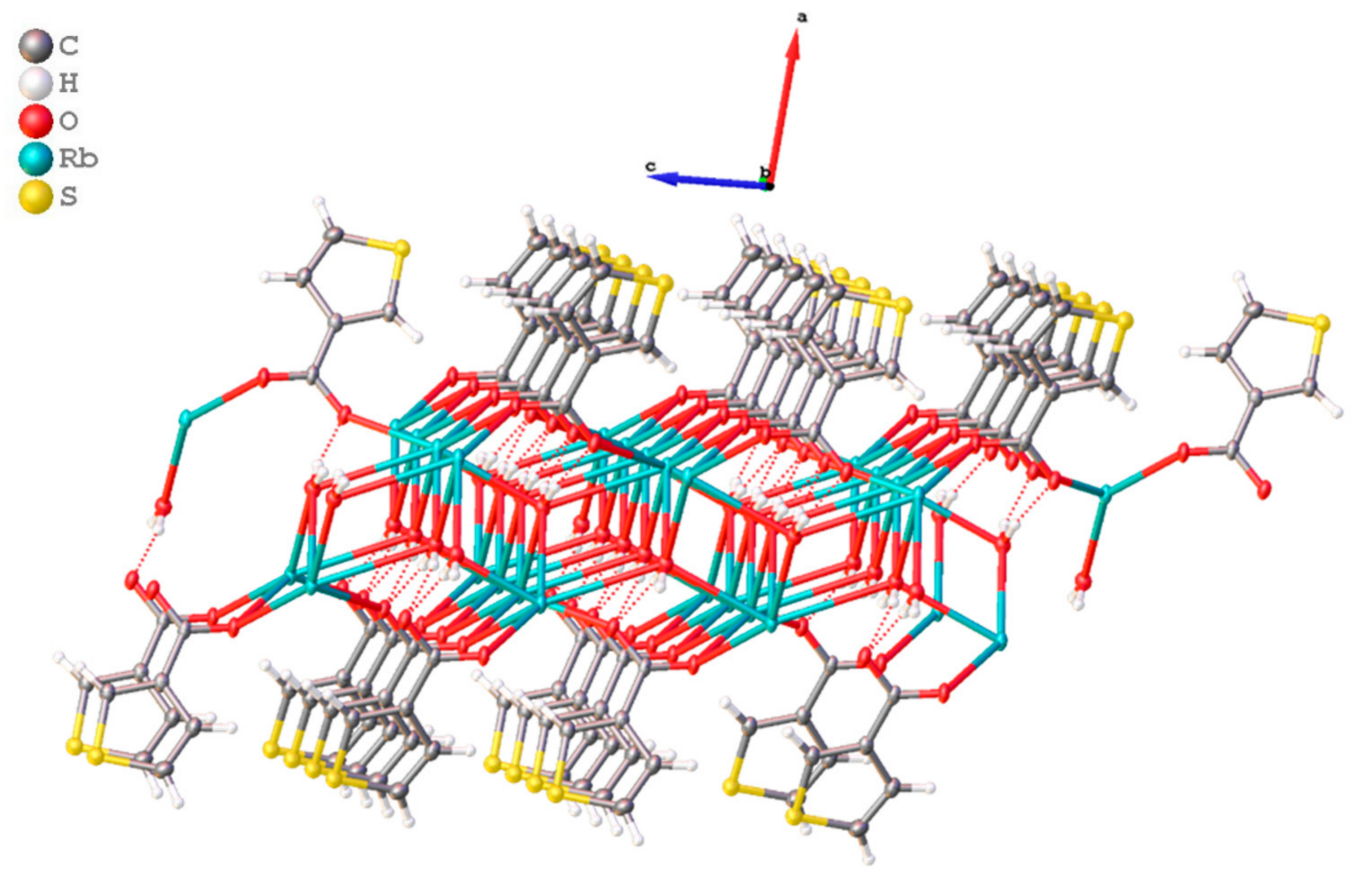
Projection along the b-axis of **1Rb** showing the 2D network.

The longest Rb–O bond is from a water oxygen, Rb1–O3#8 (3.278(3) Å), and the shortest bond is for a bridging carboxylate Rb1–O1 (2.828(2) Å). The coordinated water oxygens O3 and O3#4 appear to connect the wave-like 1D polymers into a two-dimensional (2D) chain (Figure 7). All four water oxygen atoms found in the Rb coordination environment are involved in hydrogen bonding to carboxylate-O (Figure 6, Table S12), forming an extensive network of hydrogen bonding and further linking the 2D network together.

The polymeric complex [Cs{H(3tpc)_2_}]_n_ (**1Cs**) crystallises in the space group *C*2*/c.* Although the reaction stoichiometry was intentionally maintained at 1:1 Cs:3tpcH to avoid the incorporation of excess unreacted proligands in the structure, only partial deprotonation of the carboxylic acid group occurred. As a result, a hydrogen atom is symmetrically shared between two carboxylate groups, making a (HL_2_)^−^ ligand. The Cs atom is eight-coordinate with cubic geometry. In the asymmetric unit, the ligand bridges Cs1 and Cs1#2 via O1, and the only binding mode observed for the structure is µ_4_-1κ(O):2κ(O):3κ(O′):4κ(O′). Even though the crystal structure of **1Cs** does not indicate the presence of either coordinated or lattice water molecules, the TGA of the bulk sample revealed the presence of 0.5 water molecules.

**Figure 8:**
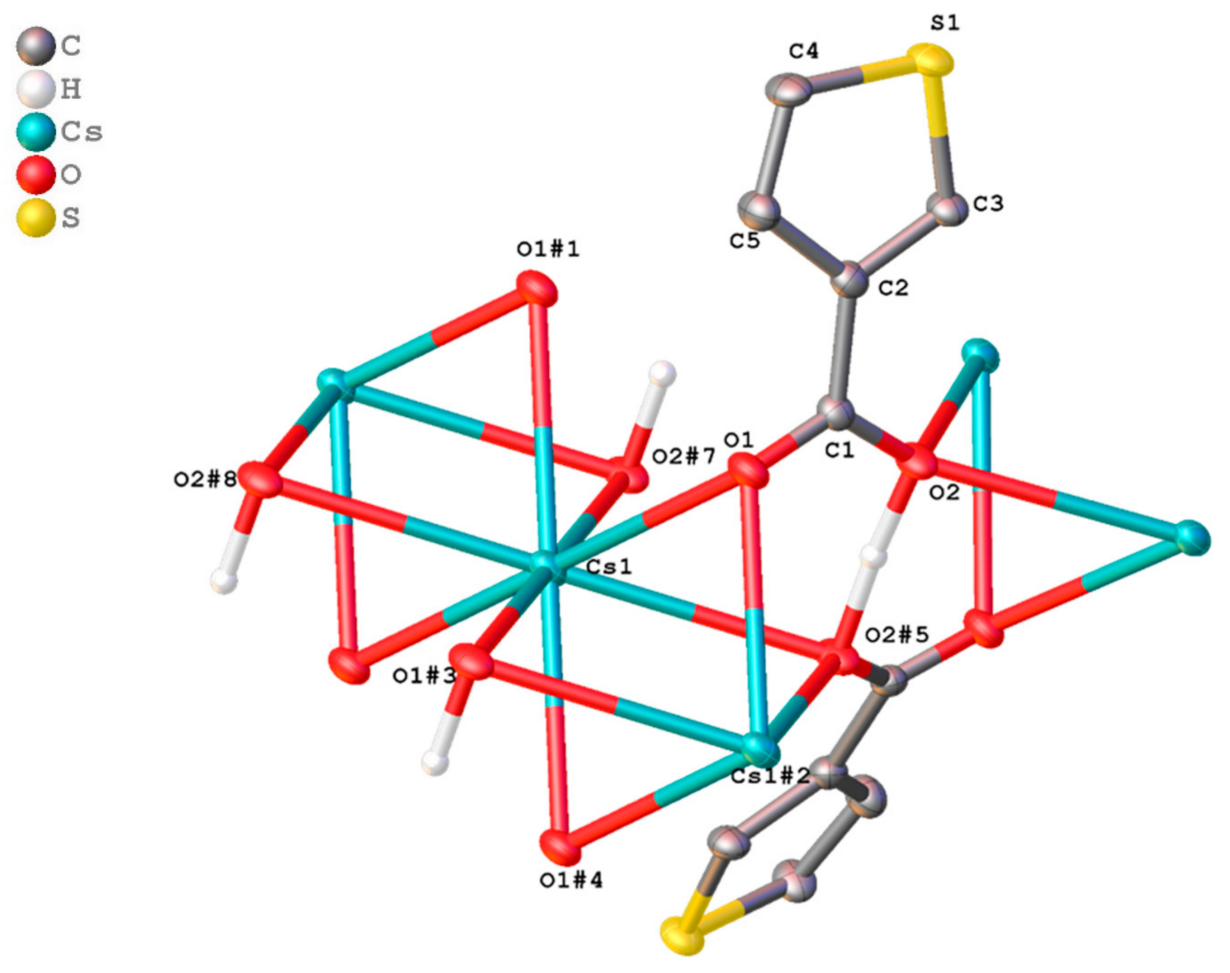
The asymmetric unit of [Cs{H(3tpc)_2_}]_n_ **1Cs** expanded to illustrate the Cs coordination, highlighting the presence of a centrosymmetric hydrogen atom located between the two carboxylate groups. Symmetry code: ^#1^ + X,1 + Y, + Z; ^#2^ + X,-1 + Y, + Z; ^#3^ 1-X,2-Y,1-Z; ^#4^ 1-X,1-Y,1-Z; ^#5^ 1-X, + Y,1/2-Z; ^#7^ 1-X,1 + Y,1/2-Z; ^#8^ + X,2-Y,1/2 + Z.

**Figure 9:**
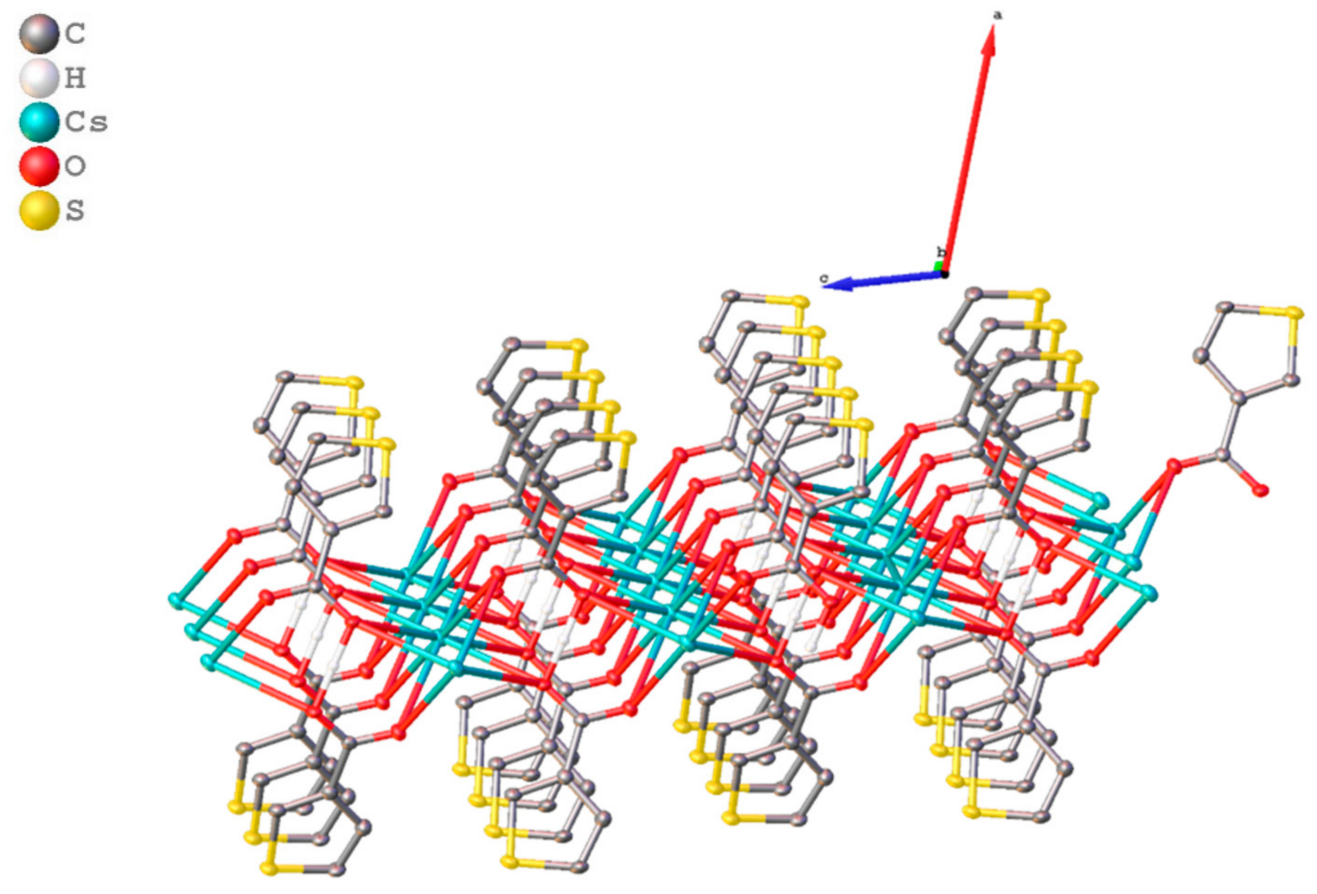
The crystal packing of **1Cs** is shown in a b-axis projection. The hydrogen atoms, except for those forming symmetrical hydrogen bonds, have been omitted for clarity.

The observed partial deprotonation results in both the Cs^+^ cation and O–H hydrogen atom being located on crystallographic inversion centres. In the asymmetric unit, both entities carry an effective charge of 0.5+, effectively counterbalancing the negative charge of the 3tpc^-^ anion. The oxygen atoms of the carboxylate groups from the two 3tpc ions are involved in forming symmetrical bonds with the hydrogen atom located on the inversion centre (Figure 8). This results in O–H bond lengths of 1.232(4) Å, which are longer than those of a typical O–H covalent bond (0.97 Å) [34], but shorter compared to the lengths observed in O..H hydrogen-bond interactions. The symmetrical hydrogen bond that is observed is commonly accompanied by a significantly short O...O distance ( 2.460 Å here). The coordinated O from the 3tpc^−^ anion bridges the 1D polymers, forming a two-dimensional (2D) chain (Figure 9).

Despite multiple attempts to refine the hydrogen atom as disordered, appearing on either side of the inversion centre, it consistently reverted back to the inversion centre. Determining the precise location of a hydrogen atom solely based on X-ray diffraction data presents a challenge. Nonetheless, by examining the distance between oxygen atoms and analysing the hydrogen atom behaviour during refinement, we have confidence in its location. Therefore, we can conclude that only the partial deprotonation of 3tpcH occurs in **1Cs**.

#### 2.2.2. M(2m3fur) Complexes (2M = 2Li, 2K)

**Figure 10:**
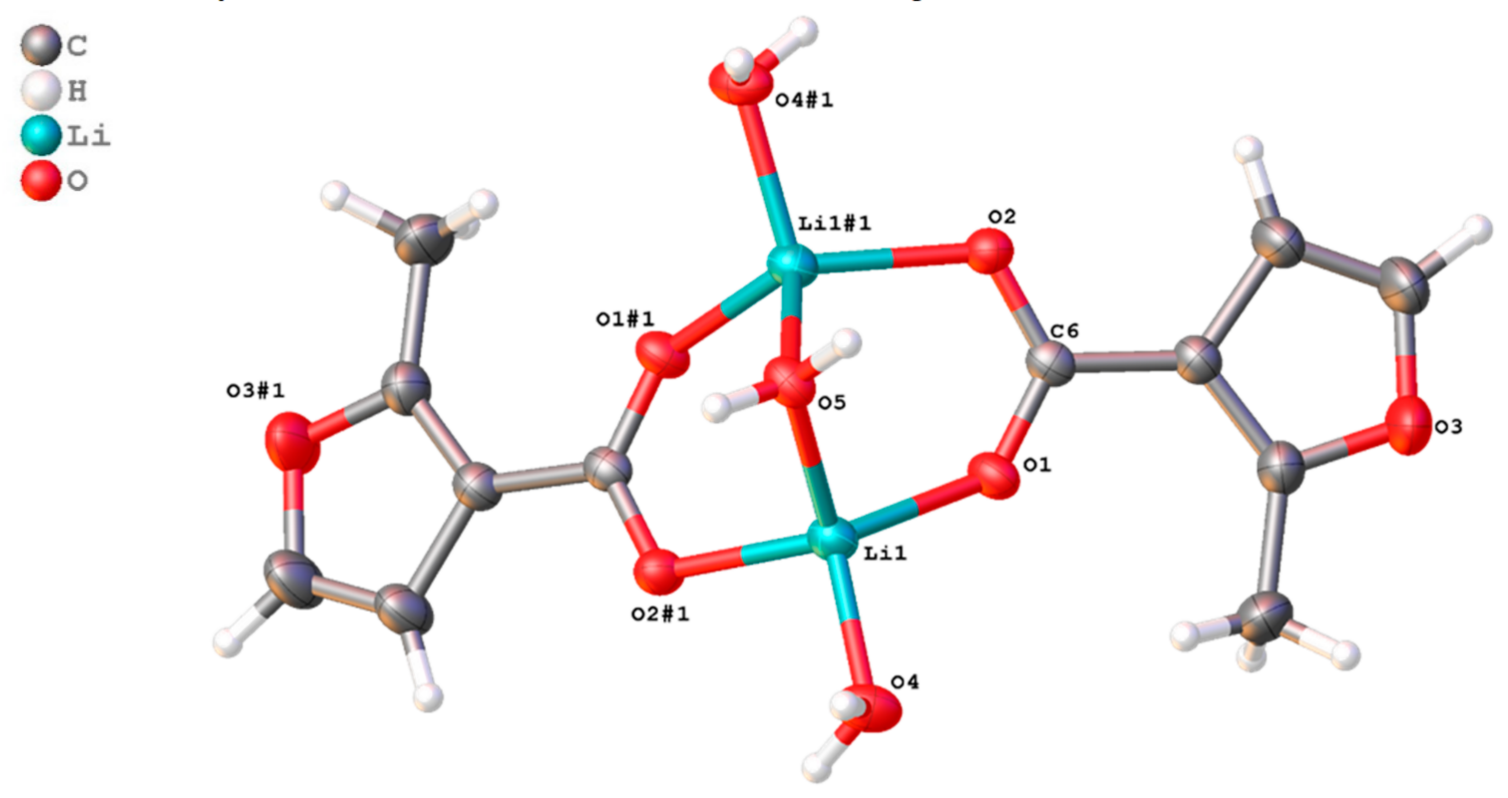
Molecular diagram of the dinuclear [Li_2_(2m3fur)_2_(H_2_O)_3_] (**2Li**). Symmetry code: ^#1^ 1-X, + Y,1/2-Z.

The dinuclear unit of [Li_2_(2m3fur)_2_(H_2_O)_3_] (**2Li**) consists of two tetracoordinated lithium metal centres, and it crystallises in the orthorhombic *Pbcn* space group. The stereochemistry around each metal is tetrahedral (Figure 10).

The complex **2Li** contains two *syn–syn* µ-1κ*O*:2κ*O′* carboxylate ligands (O(1,2) and symmetry-related O(1#1,2#1) and three-coordinated (O4, O4#1,O5) water molecules. The Li1…Li1#1 separation measured is 2.758(8) Å. The shortest Li-O bond is formed by the ligated water molecule O4 and its symmetry equivalent O4#1 (1.893(3) Å), while the longest bond is Li1-O5 (2.034(3) Å) of the bridging water molecule. The bridging oxygen atoms of the carboxylate ligands form an angle of 123.49(14)° at O1-Li1-O2#1 (and the symmetry-equivalent O1#1-Li1-O2). The O5 oxygen atom from a coordinated water molecule bridges the two metal atoms of the dinuclear complex, forming a Li1-O5-Li1#1 angle of 85.41(15)°. The angle between the bound water molecules and the metal is *cisoid* for O4-Li-O5 and its symmetry equivalent, O4#1-Li1#1-O5 (116.51(13)°).

The two-dimensional polymer [K_2_(2m3fur)_2_(H_2_O)]_n_ (**2K**) crystallises in the monoclinic *P*2_1_*/c* space group. Despite the crystal structure being a monohydrate, thermal analysis of the bulk precipitate indicated the presence of an extra water molecule. The asymmetric unit of the compound contains two distinct potassium metal centres. K1 is eight-coordinated; but it can be considered as pseudo-seven-coordinated if the carboxylate takes one coordinated site, resulting in a distorted pseudo-pentagonal bipyramidal stereochemistry. Thus, O4 and a chelating carboxylate are in axial positions, with an O4-K1-C7#5 angle of 168.53 (4)°, while O1, O1#5, O2#4, O7#2, and O7#6 are in equatorial positions. The six-coordinated K2 can be regarded as distorted pseudo-square pyramidal if the carboxylate is considered to occupy one coordination position. In this case, four basal oxygen atoms (O2#3, 4#1, 5, 7) form a square plane, while the carboxylate is in the axial position.

**Figure 11:**
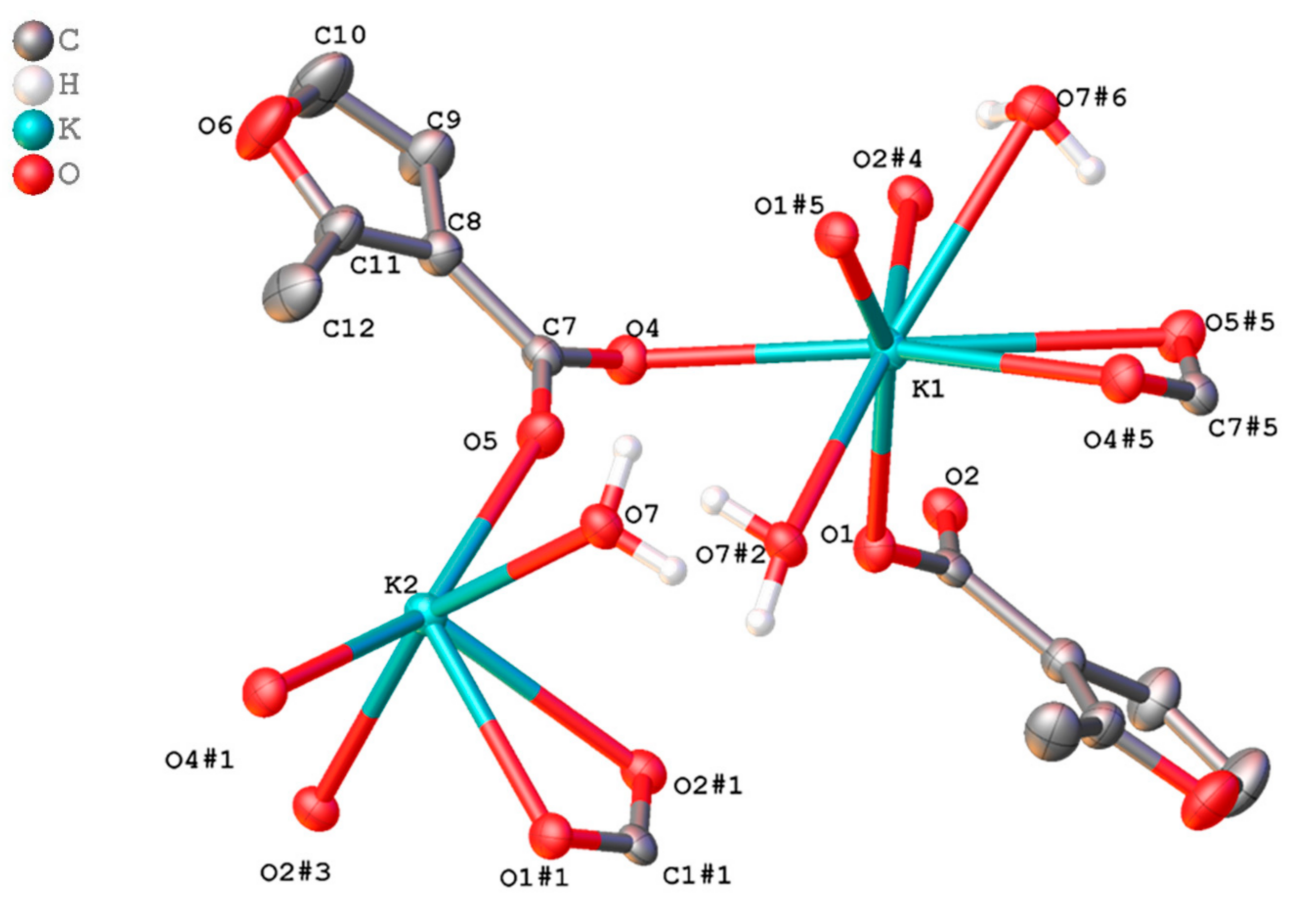
The expanded asymmetric unit of [K_2_(2m3fur)_2_(H_2_O)]_n_ **2K** extended to show the K1 and K2 coordination sphere. Symmetry code: ^#1^ + X,3/2-Y,-1/2 + Z; ^#2^ 1-X,1-Y,1-Z; ^#3^ 1-X,1/2 + Y,3/2-Z; ^#4^ 1-X,1-Y,2-Z; ^#5^ 1-X,-1/2 + Y,3/2-Z; ^#6^ + X,1/2-Y,1/2 + Z. ^#7^ 1-X,1 + Y,1/2-Z; ^#8^ + X,2-Y,1/2 + Z.

The carboxylate ligand binds in two unique modes: µ_5_-1κ(O);2κ(O);3κ(O,O′);4κ(O′);5κ(O′) and µ_4_-1κ(O);2κ(O);3κ(O,O′);4κ(O′). Eight-coordinated K1 is ligated by two chelating oxygen atoms (O4#5,5#5) of a µ_4_-1κ(O);2κ(O);3κ(O,O′);4κ(O′) linked carboxylate, bridging oxygen atoms (O1, O1#5, O2#4) of three µ_5_-1κ(O);2κ(O);3κ(O,O′);4κ(O′);5κ(O′) ligands, one bridging oxygen atom (O4) of a µ_4_-1κ(O);2κ(O);3κ(O,O′);4κ(O′) oxylatecarb, and two ligated water oxygen atoms (O7#2,7#6). K2 is surrounded by a µ_5_-1κ(O);2κ(O);3κ(O,O′);4κ(O′);5κ(O′) chelate (O1#1,2#1), two bridging oxygen atoms from a µ_4_-1κ(O);2κ(O);3κ(O,O′);4κ(O′) (O5,O4#1) ligand, one bridging oxygen atom from µ_5_-1κ(O);2κ(O);3κ(O,O′);4κ(O′);5κ(O′) (O2#3) bridges, and one ligated water oxygen atom (O7), resulting in a six-coordinate metal atom (Figure 11).

The repeating asymmetric units in the one-dimensional polymeric chain are oriented in opposite directions with a slight wave. The propagation of the complex into a 1D polymer occurs through bridged K2…K1…K2#7…K1#7…K2#8 chains, and four-membered rings of K1–O4–K2#7–O2#4 are formed between the adjacent asymmetric units in the 1D polymeric chain. Adjacent metal centres in **2K** have K2…K1 separations of 6.1085(12) Å and K1…K2#7 of 3.6743(8) Å. The average K–O bond lengths observed in **2K** are as follows: K1 with a bond length of 2.841 Å, and K2 with a bond length of 2.750 Å. The longer bond length observed for K1 can be attributed to its higher coordination number. The K–O bond distances vary within the structure, ranging from the shortest bond involving bridging K2-O5 (2.6548(12) Å) to the longest bond involving chelating K1-O5#5 (3.0462(13) Å). The latter bond length is consistent with K1 being the eight-coordinated metal centre in **2K**.

**Figure 12:**
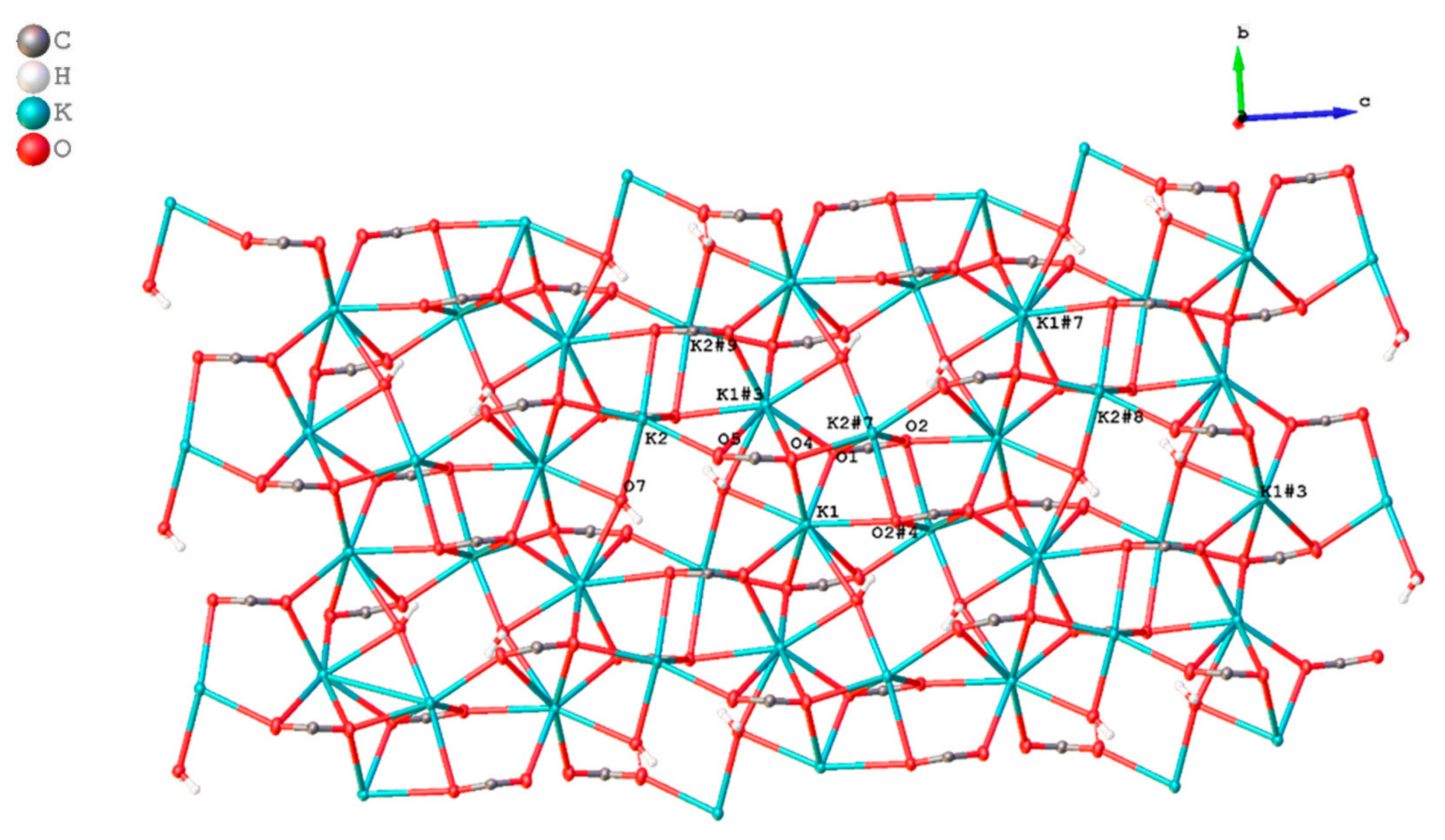
An a-axis projection of the crystal packing of **2K** (2-methyl-3-furan rings have been omitted for clarity). Symmetry code: ^#3^ 1-X,1/2 + Y,3/2-Z; ^#4^ 1-X,1-Y,2-Z; ^#7^ 1-X,1 + Y,1/2-Z; ^#8^ + X,2-Y,1/2 + Z; ^#9^ 1-X,2-Y,1-Z.

The adjacent polymeric chains are oriented in opposite directions, and they further extend to form a 2D network through the bridging of the water oxygen atoms, as well as the carboxylate oxygen, from within the chain to the neighbouring chain (Figure 12).

##### 2.2.3. 2D Polymeric Chain of the [Li(3fur)]_n_ (3Li) Complex

**Figure 13:**
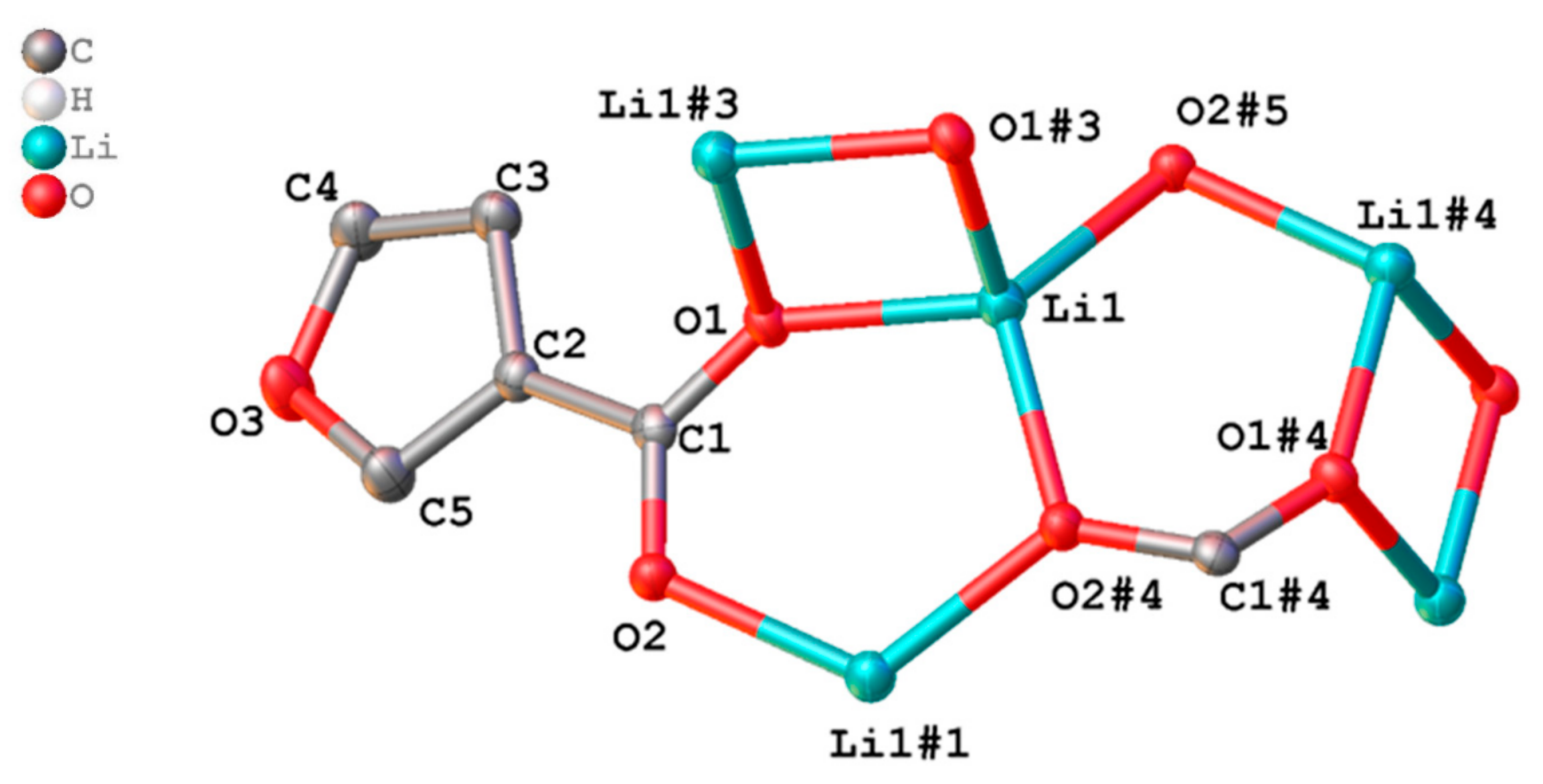
The asymmetric unit of [Li(3fur)]_n_ (**3Li**) expanded to show the Li coordination sphere. Symmetry code: ^#1^ 1-X,1/2 + Y,3/2-Z; ^#3^ 1-X,-Y,1-Z; ^#4^ 1-X,-1/2 + Y,3/2-Z; ^#5^ + X,-1 + Y, + Z.

The complex [Li(3fur)]_n_ (**3Li**) forms a 2D polymeric sheet with tetracoordinated lithium ions and crystallises in the monoclinic *P*2_1_*/c* space group with a tetrahedral stereochemistry. Four µ_4_ -1κ(O);2κ(O);3κ(O′);4κ(O′) oxygen atoms (O1, O1#3, O2#4, O2#5) complete the coordination sphere around the metal centre (Figure 13).

**Figure 14:**
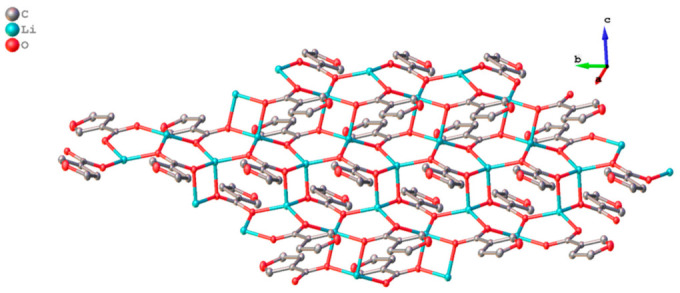
Crystal packing of **3Li** into a **2D** network (aromatic H atoms- are omitted for clarity).

The Li–O bond lengths range from 1.935(3) Å (Li1-O1) to 2.025(3) Å (Li1-O1#3), with an average bond length of 1.978 Å. The two unique, alternating six-membered rings formed by the five-bridging oxygen atoms O1, O2, O2#4, O1#4, and O2#5 form the backbone of the 1D polymeric chain, where Li1#1…Li1…Li1#4 forms an angle of 114.93 (16)° with Li1-Li#1 and Li1-Li1#4 separations of 3.155(3) Å. The successive 1D chains are oriented in opposite directions, thereby constructing two types of chains. The carboxylate oxygen atoms O1, O1#3 of the carboxylate µ_4_-1κ(O);2κ(O);3κ(O′);4κ(O′) ligand bridge lithium metals of neighbouring chains, leading to the formation of a two-dimensional (2D) network with a Li1-Li#3 distance of 2.758(5) Å (Figure 14).

#### 2.2.4. M(4hocin) Complexes (4M = 4K, 4Rb, 4Cs)

**Figure 15:**
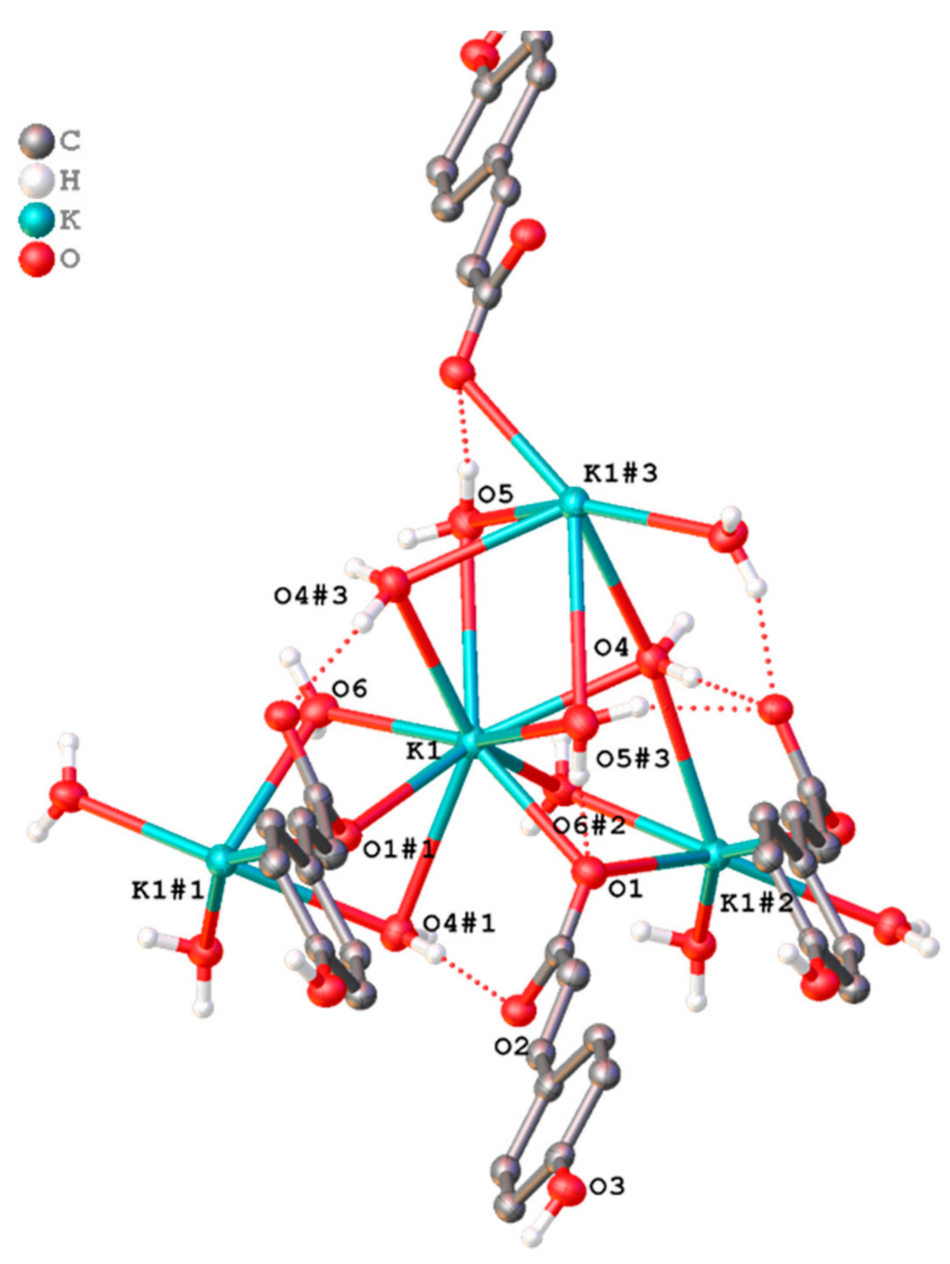
The immediate coordination environment of potassium in [K(4hocin)(H_2_O)_3_]_n_ (**4K**) (hydrogen atoms, except for those forming H-bonds, are omitted for clarity). Symmetry code: ^#1^ + X,3/2-Y,1/2 + Z; ^#2^ + X,3/2-Y,-1/2 + Z; ^#3^ -X,2-Y,-Z.

The 2D polymeric complex [K(4hocin)(H_2_O)_3_]_n_ (**4K**) crystallises in the space group *P*2_1_*/c* with a distorted tricapped trigonal prismatic geometry. The nine-coordinate potassium atoms are each attached to two µ-1κ(O);2κ(O) carboxylates and seven oxygen atoms from aqua ligands. The water oxygen atom O4 is bound to three potassium atoms (K1, K1#2, K1#3), while O5 and O6 are each bound to two potassium atoms: K1 and K1#3 and K1 and K1#1, respectively. The chain propagation exhibits a twist, with an angle of 126.48(2)° between K1#1, K1 and K1#2, and the distance between K1 and K1#1 is 3.9478 (7) Ǻ. Four ligated aqua molecules link neighbouring polymeric chains that run in opposite directions, forming a 2D network through O4, O4#3, O5, and O5#3 (Figure 15). µ_3_-H_2_O coordination is known. For recent examples, see [35,36,37].

**Figure 16:**
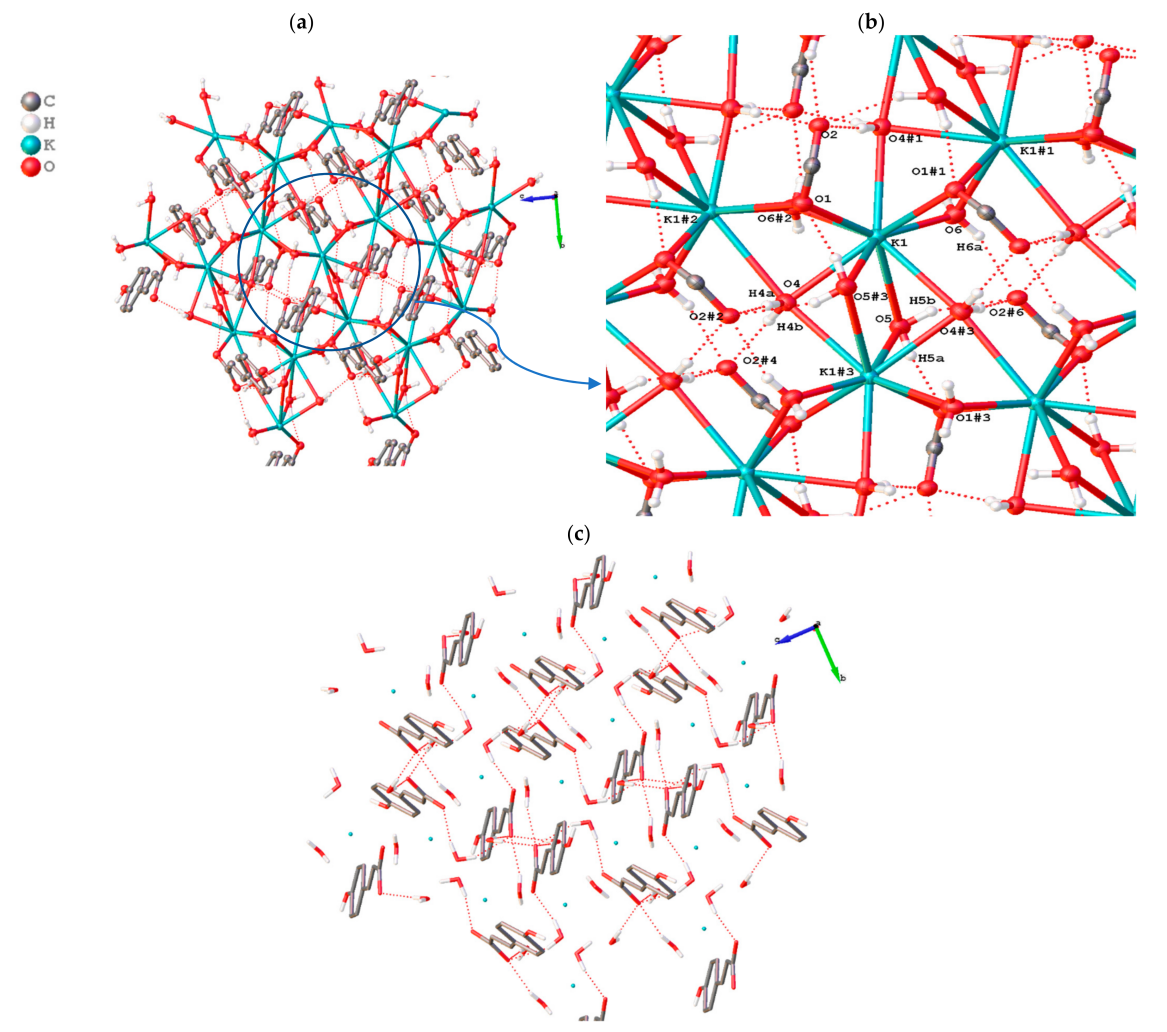
(**a**) A projection along the a-axis of **4K**. (**b**) H-bonded interaction in [K(4hocin)(H_2_O)_3_]_n._ (**c**) The hydrogen-bonded network formed by the 4hocin and water molecules. (K–O coordination bonds are omitted for clarity). Symmetry code: ^#1^ + X,3/2-Y,-1/2 + Z; ^#2^ 1-X,1-Y,1-Z; ^#3^ 1-X,1/2 + Y,3/2-Z; ^#4^ 1-X,1-Y,2-Z; ^#6^ + X,1/2-Y,1/2 + Z.

The longest metal–oxygen bond occurs between K1 and O5 (3.3762(14) Å), while the shortest bond is between K1 and O6 (2.7326(15) Å), both originating from ligated water molecules. The ligated water molecules form an extensive network of hydrogen bonding (Figure 16 and Table S12), along with the carboxylate oxygen atoms of the ligands, which further interconnect the adjacent 1D chains into a 2D intermolecular framework.

The three-dimensional polymeric complex [Rb{H(4hocin)_2_}]_n·_nH_2_O (**4Rb**) crystallises in the space group *C*2*/c*. Similar to compound **1Cs**, **4Rb** exhibits only partial deprotonation of the ligand despite utilising equal amounts of metal salt and ligand. The hydrogen atom exhibits centrosymmetric sharing between the carboxylate groups, making a [HL_2_]^−^ ligand, which is reinforced by the positioning of both the Rb^+^ cation and the O...H...O hydrogen atom on crystallographic inversion centres. A noticeably short O…O distance (2.469(2) Å) and the behaviour of the hydrogen atom observed during refinement further support this centrosymmetric arrangement and confirm the location of this hydrogen atom.

**Figure 17:**
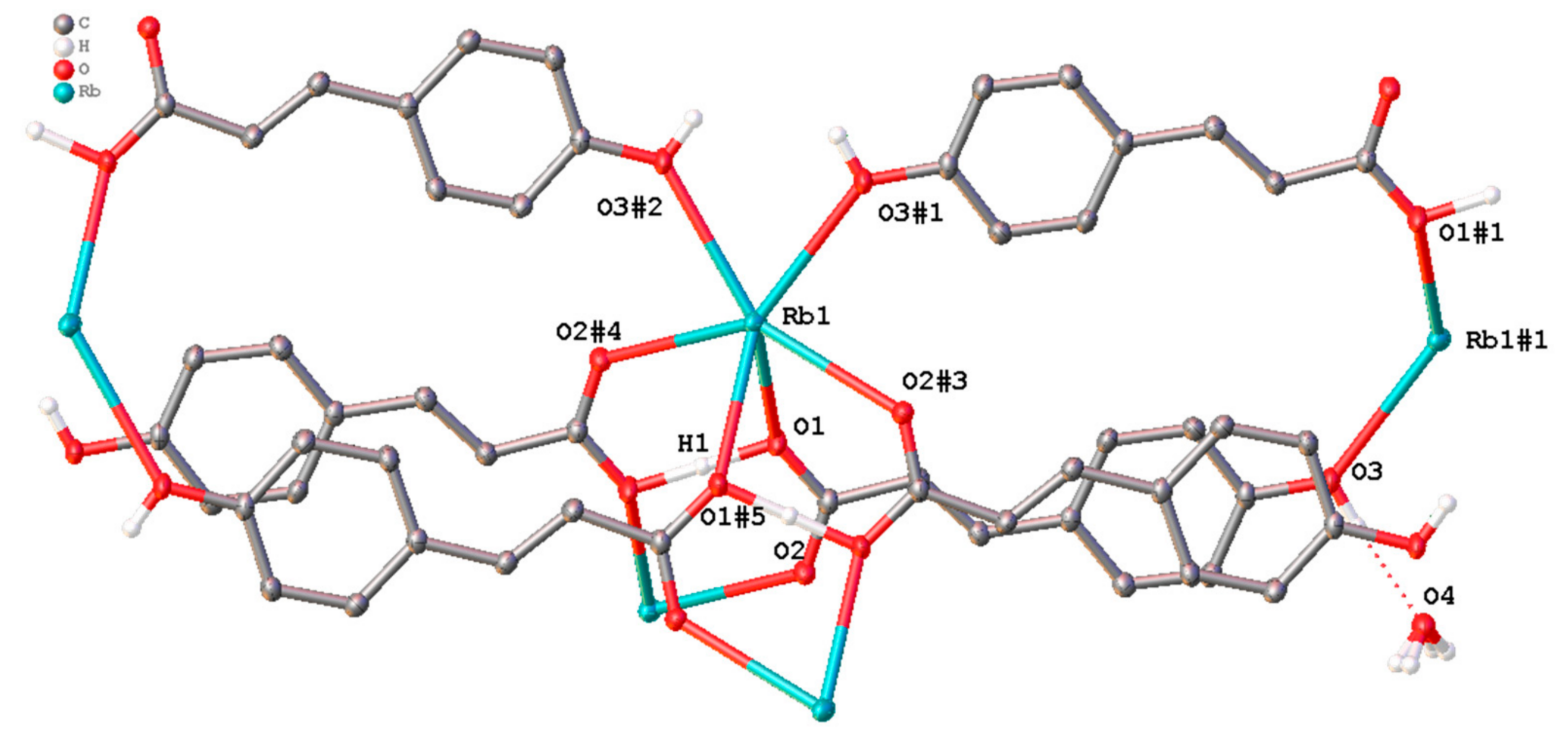
The expanded asymmetric unit of **4Rb** reveals the coordination of Rb atoms. (H atoms of the ligand, except for the phenolic hydroxyl group, are omitted for clarity; H of H(4hocin)_2_—is H1.) Symmetry Code: ^#1^ 3/2-X,3/2-Y,1-Z; ^#2^ -1/2 + X,3/2-Y,-1/2 + Z; ^#3^ + X,1-Y,1/2 + Z; ^#4^ 1-X,1-Y,-Z; ^#5^ 1-X, + Y,1/2-Z.

The asymmetric unit of **4Rb** consists of a single type of Rb atom and is expanded to depict the complete Rb coordination sphere in Figure 17. The arrangement of the six-coordinated Rb metal centre is distorted from any regular polyhedron. Contrary to **1Cs**, in the asymmetric unit of **4Rb**, the ligand is connected to Rb1 by the carboxylate oxygen atom (O1), which also binds to an H atom on the inversion centre. Within the structure, the water molecule functions as a focus for crystallisation through hydrogen bonding, and it does not form coordination bonds with the rubidium centre. The coordination sphere of **4Rb** consists of four bridging µ-1κ(O):2κ(O′) carboxylate-O atoms (O1, 1#5, 2#3, 2#4) and two phenolic-O atoms (O3#1 and O3#2) of the ligand.

**Figure 18:**
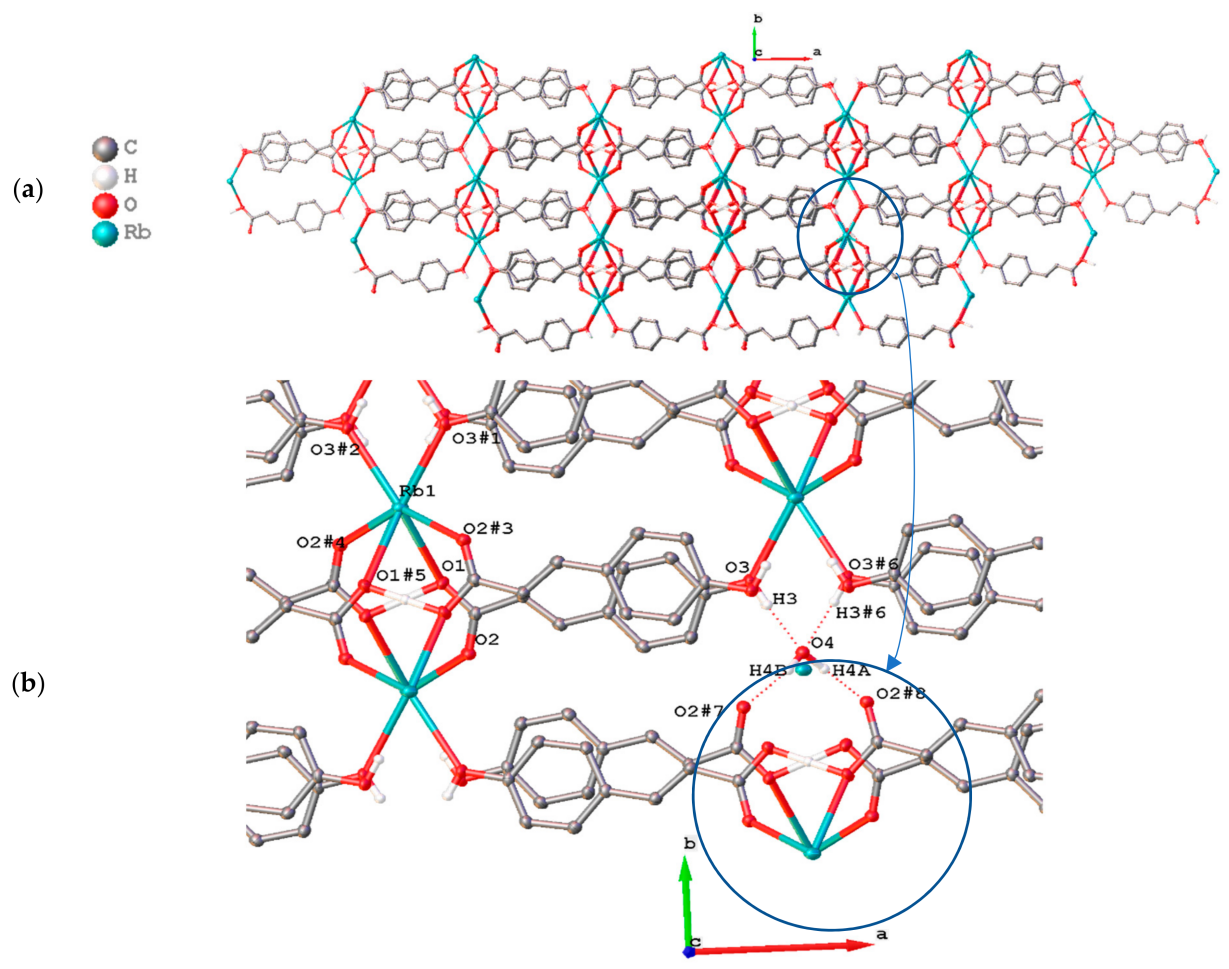
(**a**) A c-axis projection of the crystal packing of **4Rb**. (**b**) An alternative view of the crystal packing by omitting the Rb-O coordination to show the hydrogen bonds forming an extended 3D packing arrangement. ^#1^ + X,3/2-Y,-1/2 + Z; ^#2^ 1-X,1-Y,1-Z; ^#3^ 1-X,1/2 + Y,3/2-Z; ^#4^ 1-X,1-Y,2-Z; ^#5^ 1-X,-1/2 + Y,3/2-Z; ^#6^ + X,1/2-Y,1/2 + Z. ^#7^ 1-X,1 + Y,1/2-Z; ^#8^ + X,2-Y,1/2 + Z.

The Rb-O bond lengths vary from a shorter distance of 2.8868(12) Å for Rb1-O1 and the symmetry-equivalent Rb1-O1#5, involving the bridging µ-1κ(O):2κ(O′) oxygen atoms (O1 and O1#5), to a longer distance of 2.9685(13) Å between Rb1-O2#3 and Rb1-O2#4, involving the bridging µ-1κ(O):2κ(O′) oxygen atoms (O2#3 and O2#4). The metal atoms within the 1D chains are linked together by phenolic-hydroxy groups of the 4-hydroxybenzoate ligands. The adjacent chains in the 2D network are further connected by the bridging carboxylate oxygen atoms. The hydrogen bonding involving the phenolic-hydroxy groups and the carboxylate oxygen atoms of the ligand with the lattice water of crystallisation holds the neighbouring chains of polymers together, forming an extended three-dimensional packing arrangement of the polymeric chains (Figure 18 and Table S12).

**Figure 19:**
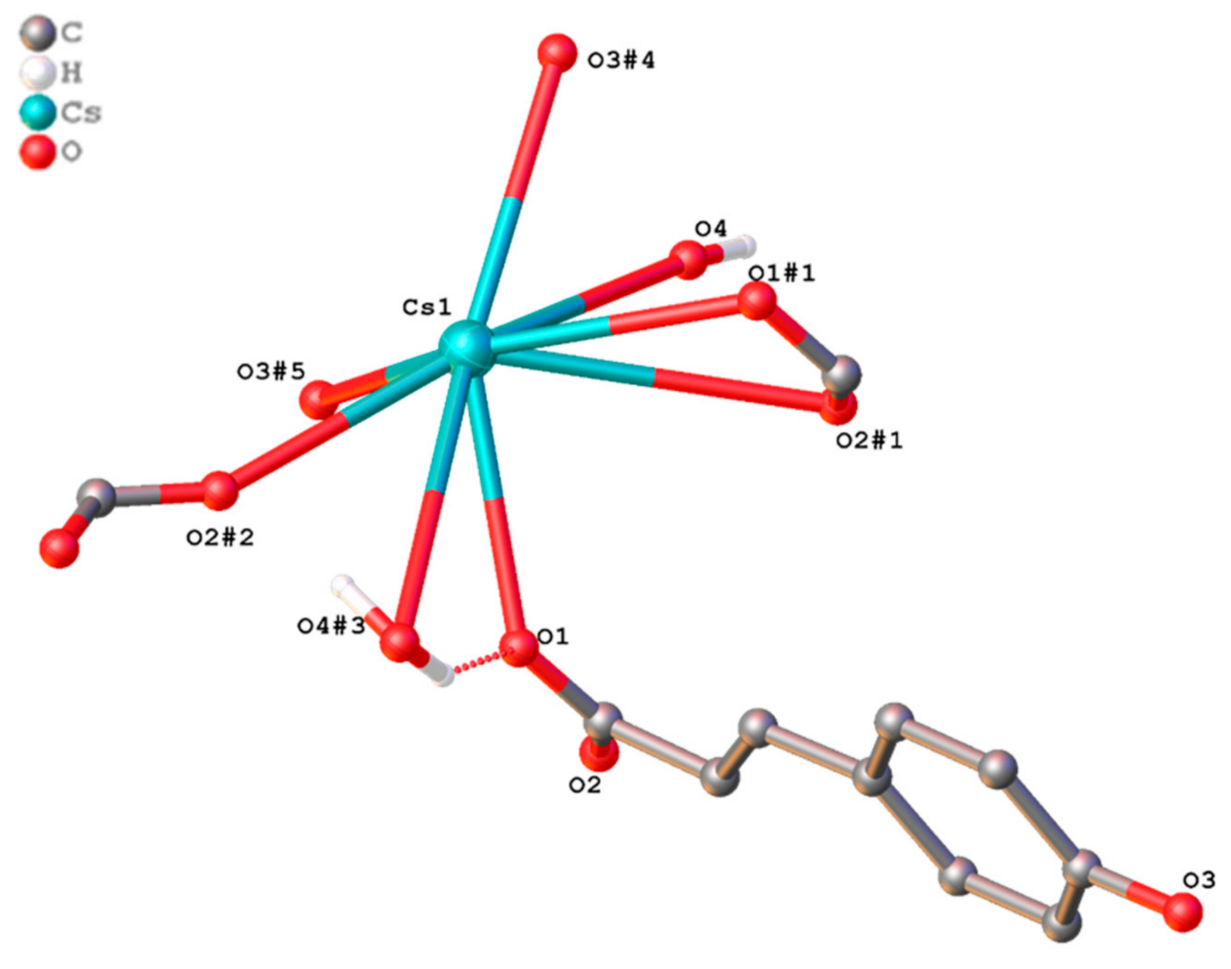
The asymmetric unit of [Cs(4hocin)(H_2_O)]_n_ (**4Cs**) expanded to show the complete Cs coordination sphere. Symmetry code: ^#1^ 1 + X, + Y, + Z; ^#2^ -X,1/2 + Y,2-Z; ^#3^ 1-X,1/2 + Y,2-Z; ^#4^ + X, + Y,-1 + Z; ^#5^ -1 + X, + Y,-1 + Z.

The X-ray crystal structure of [Cs(4hocin)(H_2_O)]_n_ (**4Cs**) reveals **a** monoclinic crystal system with a *P*2_1_ space group. The eight-coordinate caesium atoms have two bridging oxygen atoms (O1, O2#2) and two chelating oxygen atoms (O1#1,2#1) from three µ_3_-1κ(O);2κ(O,O′);3κ(O′) ligands, two water molecules (O4, O4#3), and two phenolic oxygen atoms of the ligand (O3#4, O3#5). Four oxygen atoms are close to the plane with the Cs centre, while the other four oxygen atoms sit on one side of the plane. This arrangement creates an irregular eight-coordinate polyhedron (Figure 19).

**Figure 20:**
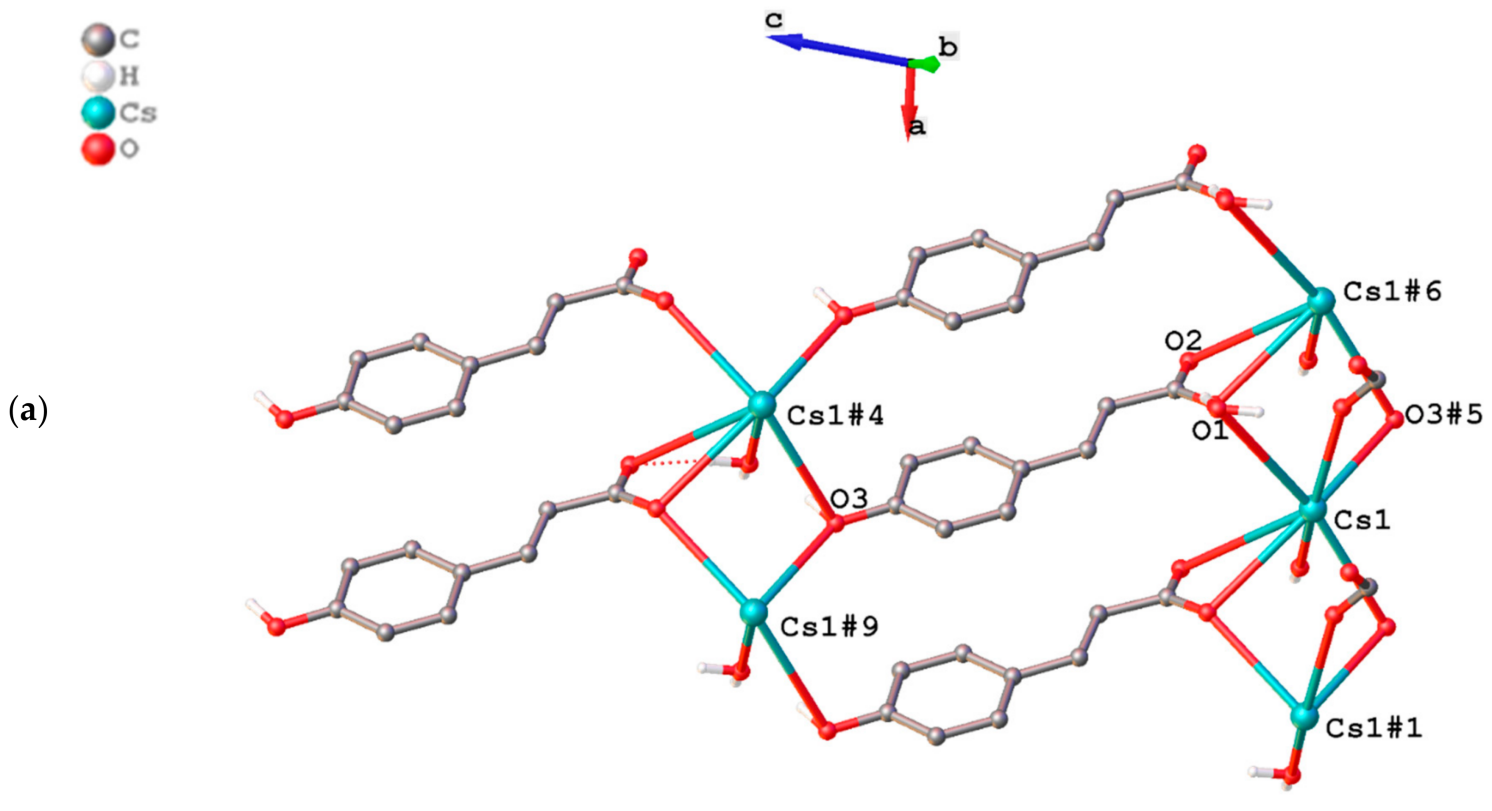

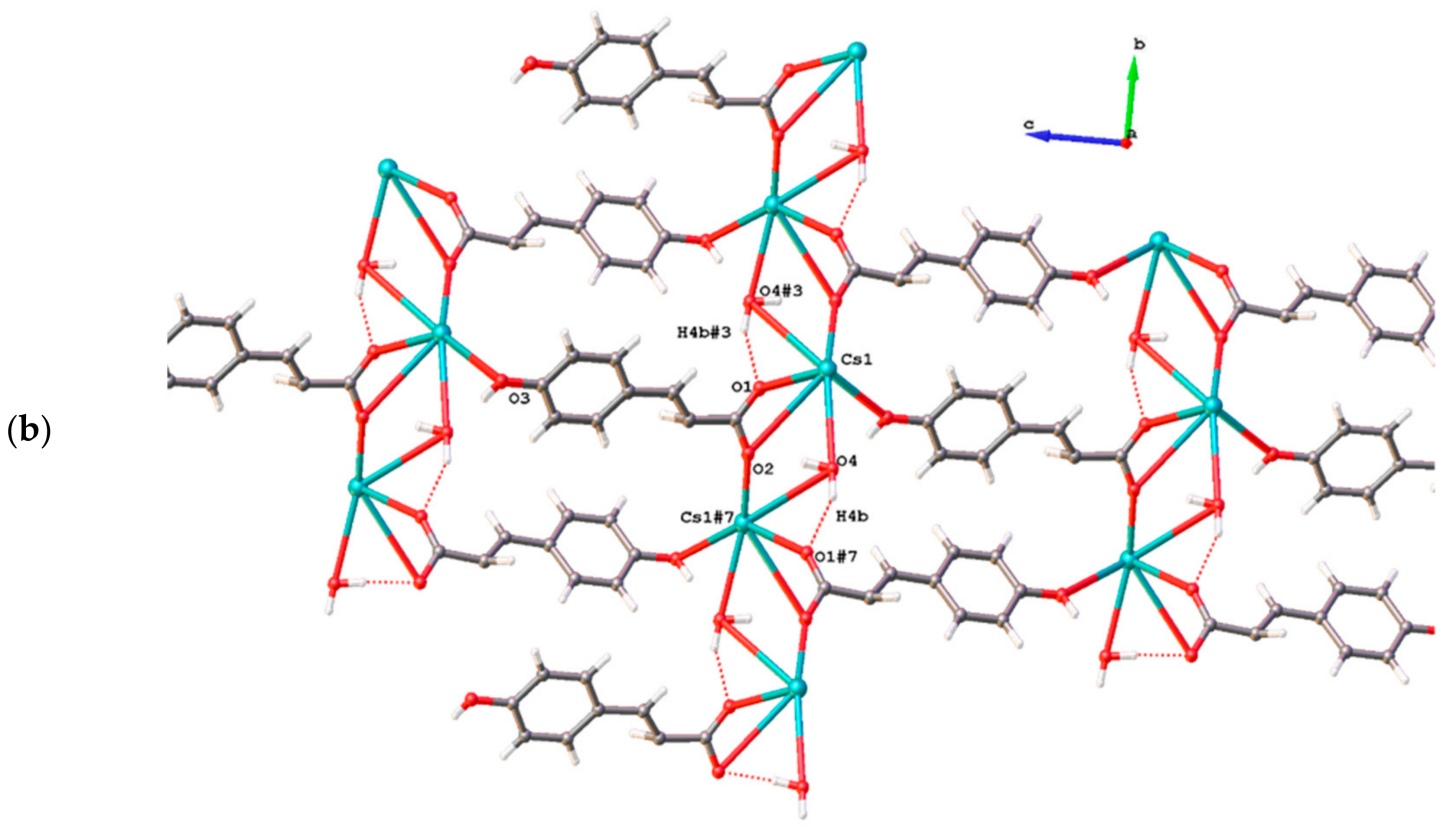
(**a**) A projection along the ac-plane shows the formation of a 2D-layer network of **4Cs** (H atoms, except for those forming H-bonds, are omitted for clarity). (**b**) A projection along the a-axis showing interaction that links the 2D networks together. Symmetry code: ^#1^ + X,3/2-Y,-1/2 + Z; ^#3^ 1-X,1/2 + Y,3/2-Z; ^#4^ 1-X,1-Y,2-Z; ^#5^ 1-X,-1/2 + Y,3/2-Z; ^#6^ + X,1/2-Y,1/2 + Z; ^#7^ 1-X,1 + Y,1/2-Z; ^#9^ 1+X,+ Y,1+Z.

The Cs-O bond lengths for the bridging carboxylates are consistently shorter than their chelating counterparts (Table S8). The angle between the bridging water oxygen atoms O4 and O4#3, which originates from the adjacent caesium atom, has an O4-Cs1-O4#3 angle of 138.84(10)°. The carboxylate-O1,2, as well as the phenolic oxygen atom O3#5, link the metal centres into a 1D chain with a Cs1…Cs1#6 distance of 4.3870(9) Å and a Cs1#1...Cs1…Cs1#6 angle of 180°. A phenolic oxygen atom, O3, bridges an adjacent metal centre in neighbouring chains, forming a 2D-layer network in the ac-plane (Figure 20a). A projection along the a-axis (Figure 20b) illustrates the interconnection of these 2D networks via a bridging polydentate ligand (via O1, 2), a coordinated aqua molecule (O4), as well as hydrogen-bonding interactions (Table S12) into an overall 3D structure.

#### 2.2.5. M(4hob) Complexes (5M = 5Li, 5K, 5Rb, 5Cs)

**Figure 21:**
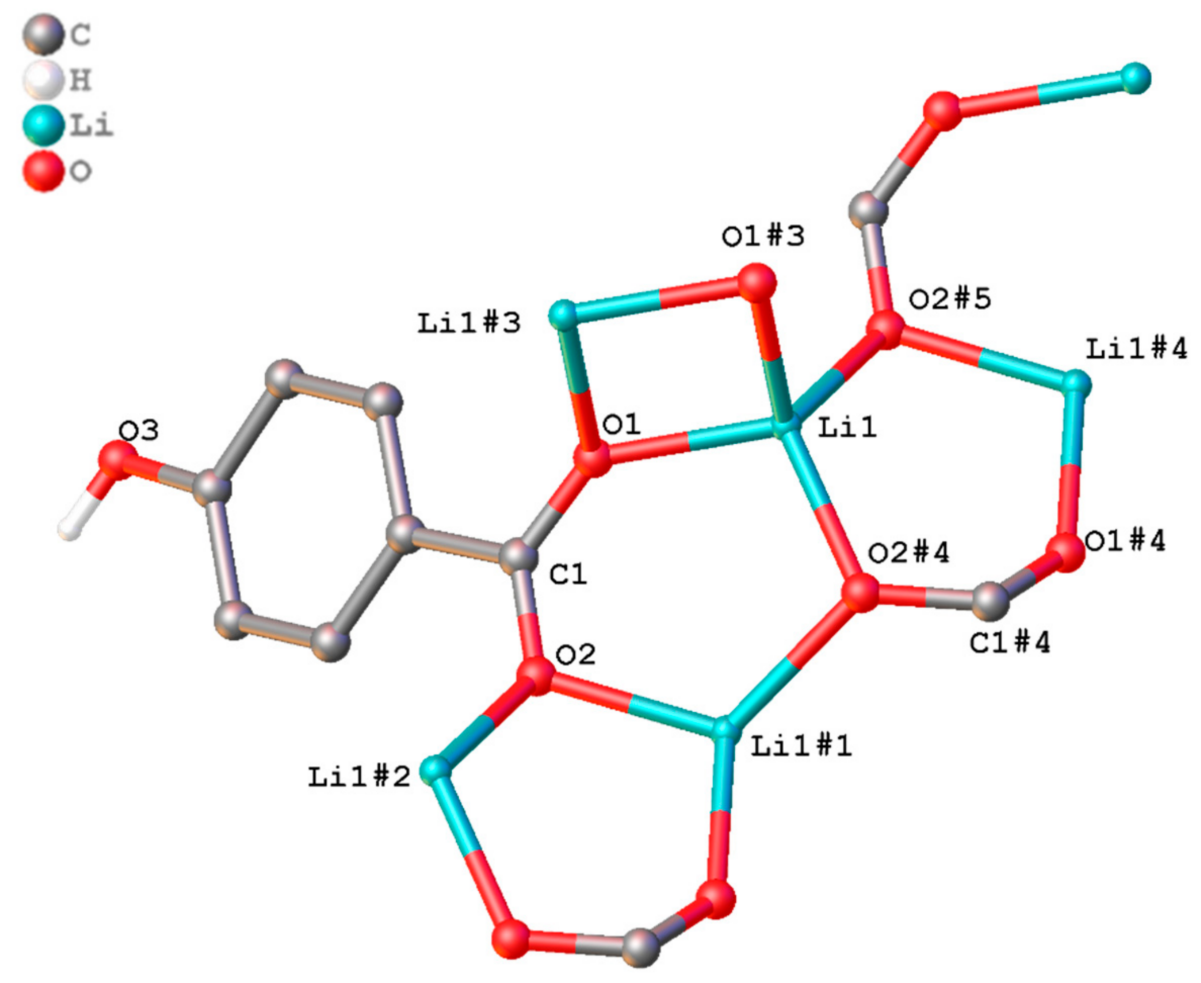
The asymmetric unit of [Li(4hob)]_n_ (**5Li**) expanded to show the Li coordination sphere. Symmetry code: ^#1^ 1-X,1/2 + Y,1/2-Z; ^#2^ +X,1 + Y, + Z; ^#3^ 1-X,-Y,1-Z; ^#4^ 1-X,-1/2 + Y,1/2-Z; ^#5^ + X,-1 + Y, + Z.

The complex [Li(4hob)]**_n_** (**5Li**) crystallises in the monoclinic *P*2_1_*/c* space group and exhibits similar ligand-binding modes and coordination environments to the **3Li** structure (Table 2). Analogous to the **3Li** structure, the structure of **5Li** also forms a 1D polymeric chain through the two alternate six-membered rings involving the bridging oxygen atoms O1, O2, O2#4, O1#4, and O2#5 (Figure 21).

**Figure 22:**
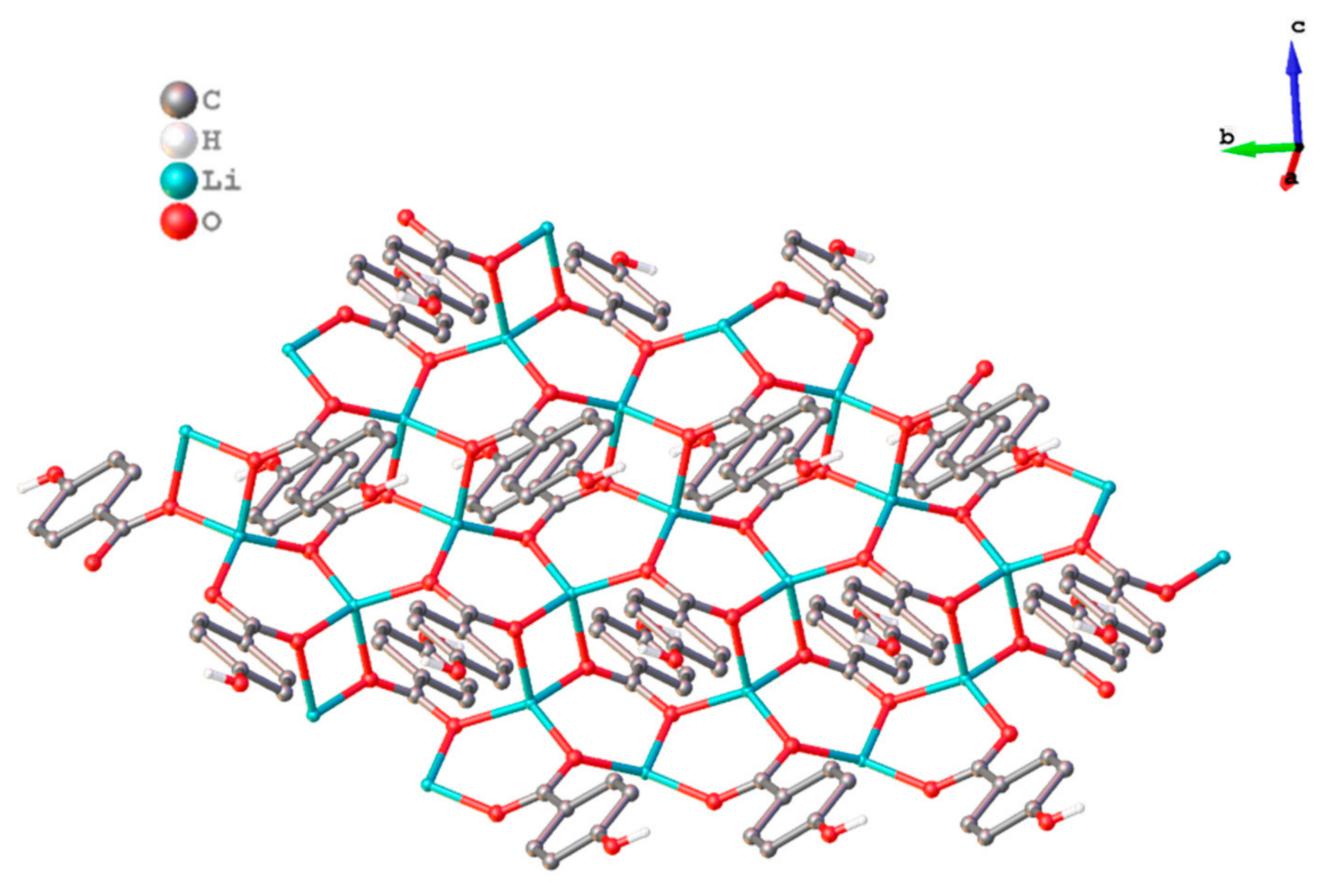
Extended diagram of **5Li** (aromatic C-H bonds are omitted for clarity).

In comparison, the compound **5Li** forms an angle of 109.6(4)° between Li1#1…Li1…Li1#4, with Li1…Li1#1 and Li1…Li1#4 separations of 3.086(8) Å. The shortest and longest Li-O bonds are attributed to Li1-O1 (1.921(7) Å) and Li-O1#3 (1.969(8) Å), respectively. A 2D layer is formed in a similar manner to the **3Li** compound through the bridging oxygen atoms (O1, O1#3) of the µ_4_-1κ(O);2κ(O);3κ(O′);4κ(O′) ligand, linking Li…Li1#3 at a distance of 2.700(14) Å (Figure 22). Although the crystallographic determination indicated the compound as anhydrous, the elemental analysis data of the complex revealed the presence of a quarter of a water molecule.

**Figure 23:**
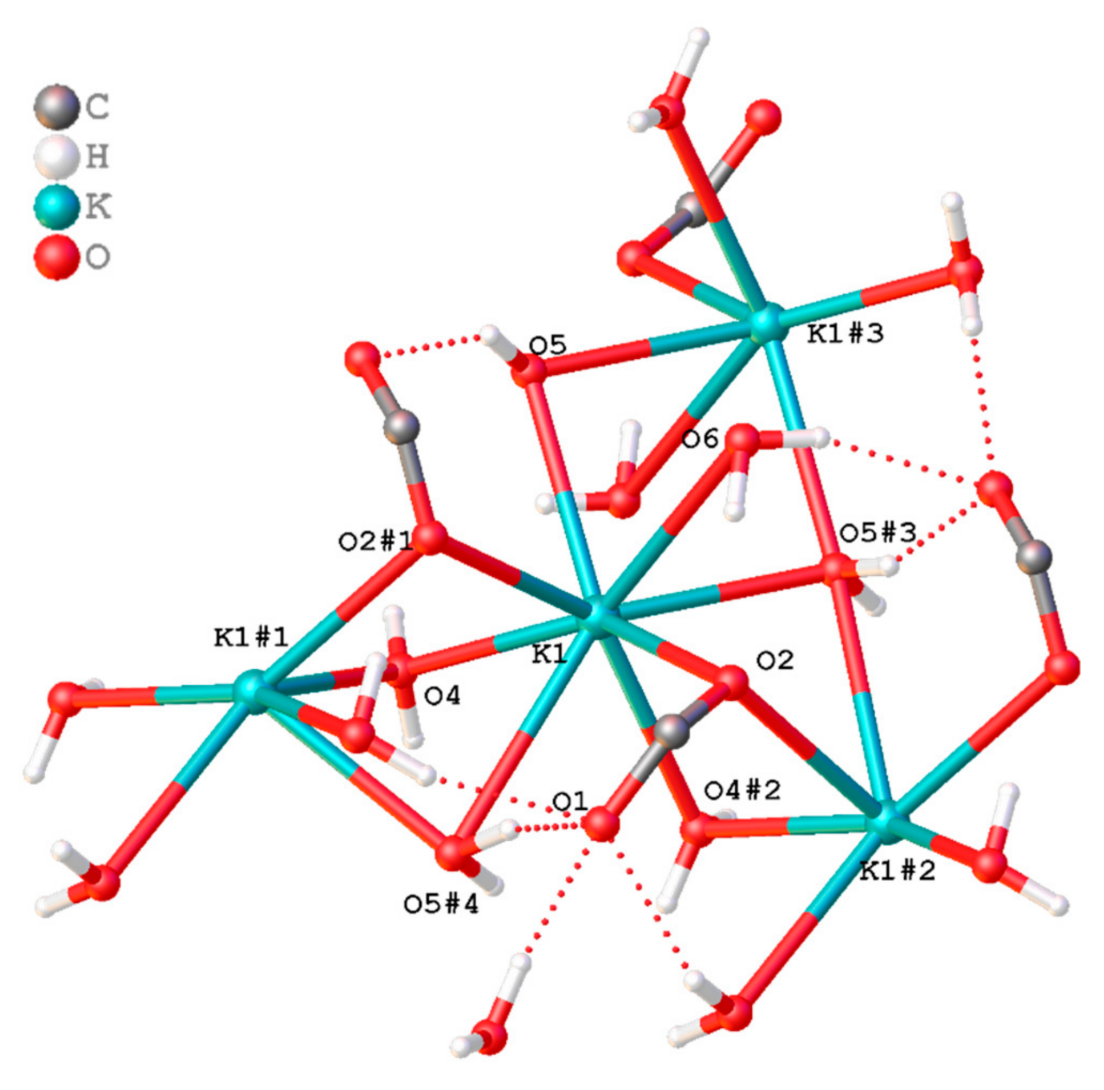
The asymmetric unit of [K(4hob)(H_2_O)_3_]_n_ (**5K**) expanded to show the K coordination. Symmetry code: ^#1^ + X,3/2-Y,-1/2 + Z; ^#2^ + X,3/2-Y,1/2 + Z; ^#3^ 2-X,2-Y,1-Z; ^#4^ 2-X,-1/2 + Y,1/2-Z.

The [K(4hob)(H_2_O)_3_]_n_ (**5K**) complex forms a 2D polymeric sheet with eight-coordinate potassium ions and crystallises in the monoclinic *P*2_1_*/c* space group with a square antiprismatic array. A partial representation of the polymeric structure is shown in Figure 23. In the asymmetric unit, there is one µ_2_-1κ(O):2κ(O) carboxylate bond (binding through O2), along with three ligated water molecules (O4, O5, and O6). The metal atom achieves a total coordination of eight by interacting with another µ_2_-1κ(O):2κ(O) oxygen atom (O2#1) and three additional ligated water oxygen atoms (O4#2, O5#3, O5#4) from adjacent metal atoms. The structure forms a nonlinear 1D polymeric chain through bridging oxygen O2, connecting K1 and K1#2 and O2#1 between the K1 and K1#1 atoms (both at 3.9243(7) Å), with K1#1…K1…K1#2 at an angle of 128.53(2)°.

**Figure 24:**
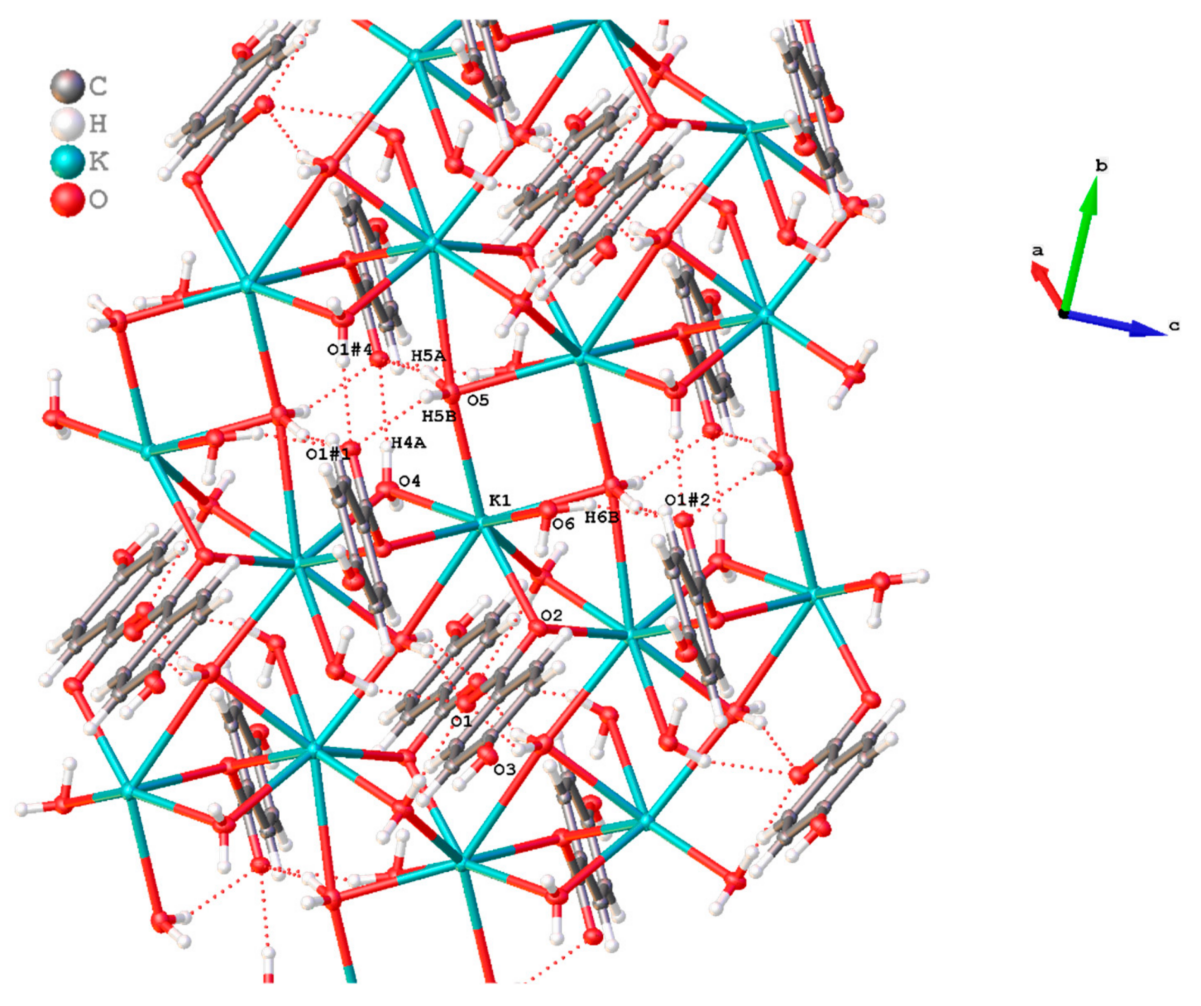
The packing diagram of the 1D polymeric chains of **5K** linked by O…H...O hydrogen bonds into a 2D network. (The 1D polymeric chain is propagating along the *c*-axis). Symmetry code: ^#1^ + X,3/2-Y,-1/2 + Z; ^#2^ + X,3/2-Y,1/2 + Z; ^#4^ 2-X,-1/2 + Y,1/2-Z.

The average K-O bond length observed in complex **5K** is 2.9123 Å, with the shortest bond being K1-O4 (2.734(13) Å), and the longest bond being K1-O5#4 (3.172(5) Å). Both the longest and shortest bonds involve aqua ligands rather than any of the bridging carboxylate oxygens. The adjacent polymeric chains connect to form a 2D network through the bridging of the water oxygen atoms (O5, O5#3). There is a significant presence of hydrogen bonding in the system, involving the carboxylate oxygen atoms and the OH groups of all six-coordinated water molecules. Figure 24 illustrates the hydrogen-bonding pattern observed, while Table S12 provides the corresponding hydrogen-bond lengths. These hydrogen bonds play a crucial role in holding the neighbouring chains of polymers tightly together, further connecting the extended two-dimensional packing. It is noteworthy that none of the phenolic-hydroxy groups participate in these hydrogen-bonding interactions.

**Figure 25:**
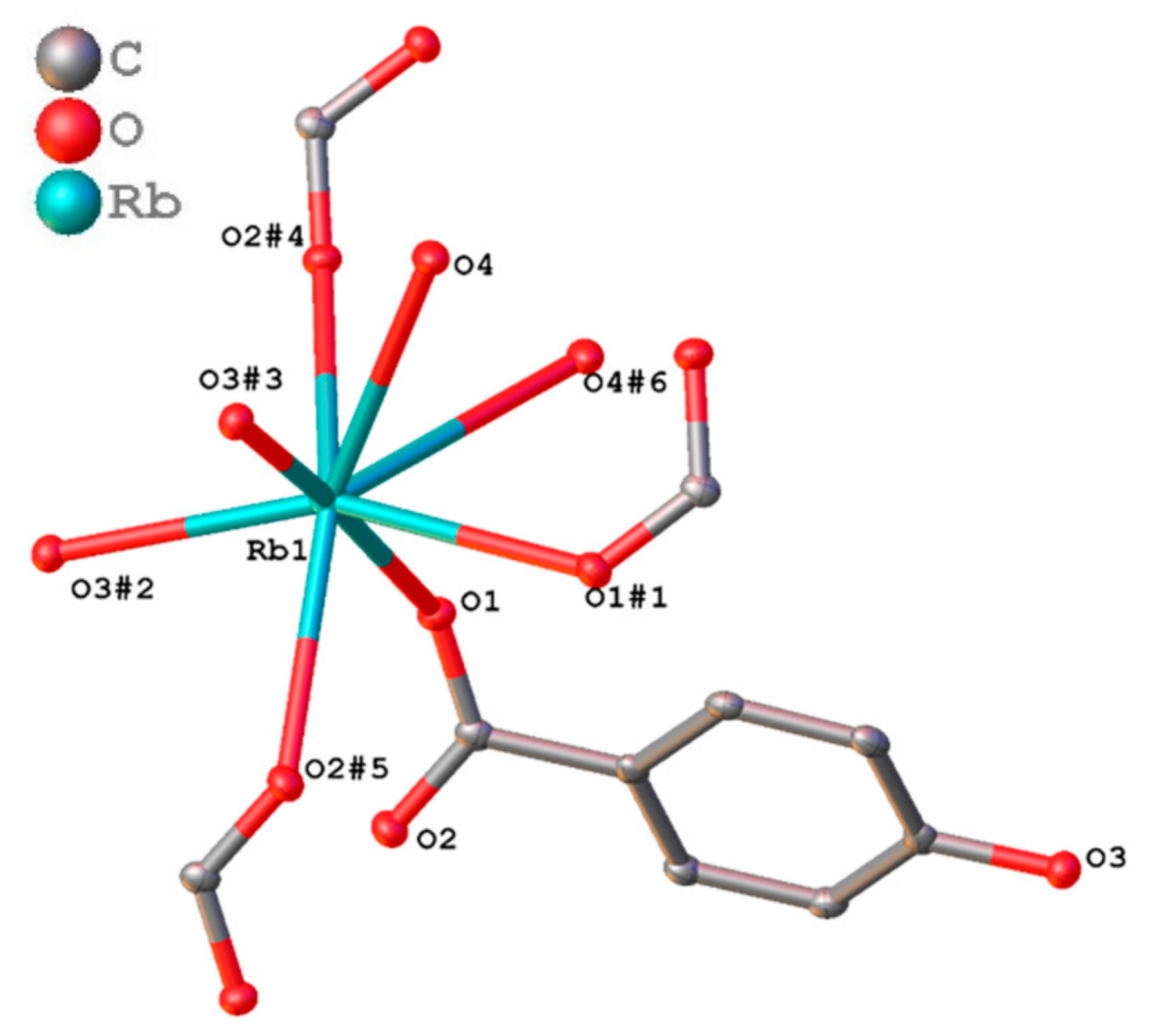
The asymmetric unit of [Rb(4hob)(H_2_O)]_n_ (**5Rb**) showing the total coordination sphere (hydrogen atoms are omitted for clarity). Symmetry code: ^#1^ + X,1/2-Y,-1/2 + Z; ^#2^ -1 + X, + Y, + Z; ^#3^ -1 + X,1/2-Y,-1/2 + Z; ^#4^ -X,-1/2 + Y,3/2-Z; ^#5^ -X,1-Y,1-Z; ^#6^ -X,-Y,1-Z.

[Rb(4hob)(H_2_O)]_n_ (**5Rb**) crystallises in the monoclinic *P*2_1_*/c* space group with a square antiprismatic donor atom arrangement around Rb. Figure 25 illustrates the structure of the extended asymmetric unit of **5Rb**.

Within the polymeric structure, there are eight-coordinate rubidium atoms bonded by four bridging carboxylate oxygen atoms (O1, O1#1, O2#4, O2#5). Additionally, two phenolic oxygen atoms (O(3#2, 3#3)) from the µ_4_-1κ(O);2κ(O);3κ(O′);4κ(O′) 4hob ligand and two coordinated water oxygen atoms (O4 and O4#6) contribute to the coordination. The metal centre achieves a total coordination of eight by incorporating another ligated water oxygen atom (O4#6) bridging from the adjacent metal centre. The binding of the phenolic oxygen atom to the metal centre is also observed in the **4Cs** structure. Each 4-hydroxybenzoate ligand in the chain forms bonds with four Rb atoms, following the same coordination pattern. For **5Rb**, the average Rb-O bond distance is 3.0486 Å. Among the Rb-O bonds, the bridging Rb1-O1 bond is the shortest (2.844(14) Å). In contrast, one of the ligated water molecules exhibits the longest Rb-O bond distance of 3.570 (16) Å (Rb1-O4#6). The two ligated water oxygen atoms form an O4-Rb1-O4#6 angle of 82.89(4)°.

**Figure 26:**
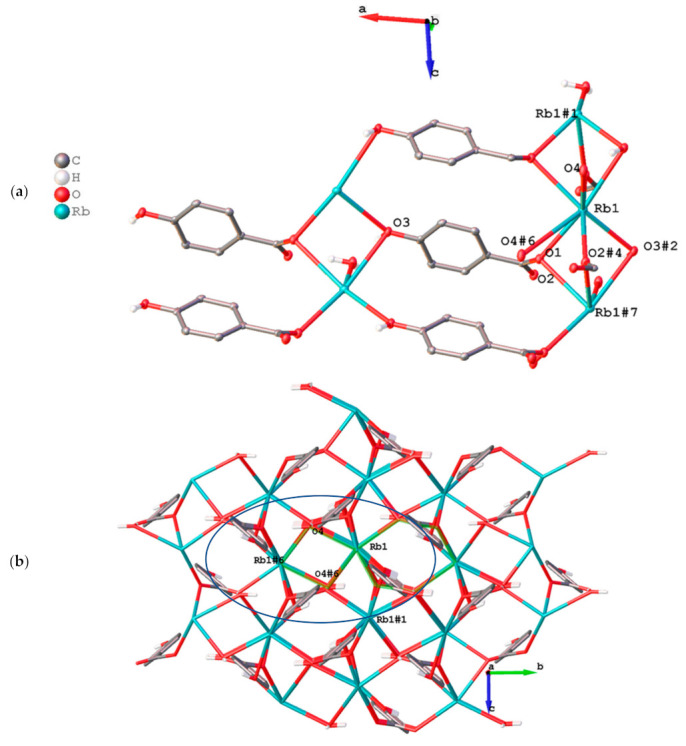
(**a**) A projection along the ac-plane showing the formation of a 2D-layer network of **5Rb**. (**b**) A projection along the a-axis of **5b** showing alternative four- and eight-membered rings (circled) that link the 2D networks together. Symmetry code: ^#1^ + X,3/2-Y,-1/2 + Z; ^#2^ 1-X,1-Y,1-Z; ^#4^ 1-X,1-Y,2-Z; ^#6^ + X,1/2-Y,1/2 + Z; ^#7^ 1-X,1 + Y,1/2-Z.

The metal atoms in the 1D chain (Rb1#1…Rb1…Rb1#7) are linked together through carboxylate-O1 and the phenolic oxygen atom O3#2 with a Rb1…Rb1#1 distance of 4.0584(8) Å at an angle of 162.109(5)°. A phenolic oxygen atom, O3, bridges to a Rb metal in a neighbouring chain, forming a 2D network in the ac-plane (Figure 26a). When projected along the a-axis (Figure 26b), it becomes apparent how these 2D networks are linked together by alternative four-membered rings (through coordinated aqua molecules (O4 and O4#4)) and eight-membered rings (through two bridging ligands), resulting in an overall 3D structure.

[Cs(4hob)(H_2_O)]_n_ (**5Cs**) crystallises in the monoclinic *P*2_1_*/c* space group and exhibits square antiprismatic eight-coordination. Although the crystal structures of **5Rb** and **5Cs** have different unit cells due to the inequivalent b-angles and a slight variation in the c-axis, the ligand-binding modes within the asymmetric units remain the same. Furthermore, the eight-coordinated metal atoms in both cases have similar coordination environments (Table 2). The 1D polymeric chain of Cs atoms is nonlinear, with Cs1#1-Cs1-Cs1#7 at an angle of 168.391(8)°. The separation of the metal atoms in the 1D chains is longer in **5Cs** (4.3322(9) Å) compared to the 4.0584 (8) Å in **5Rb**. In contrast to **5Rb**, the average Cs-O bond distance in **5Cs** is slightly longer (3.2165 Å). The disparity in the metal–metal distance and the average metal–oxygen bond distances between **5Rb** and **5Cs** aligns with the increase in the ionic radii of eight-coordinate monovalent caesium compared to rubidium [29]. Similar to **5Rb**, the bridging Cs1-O1 bond in **5Cs** is the shortest (3.012(3) Å), while the coordinated water oxygen O4#6 exhibits the longest Cs-O bond distance of 3.761(3) Å (Cs1-O4#6). The two ligated water oxygen atoms form an O4-Cs1-O4#6 angle of 81.89(4)°.

**Figure 27:**
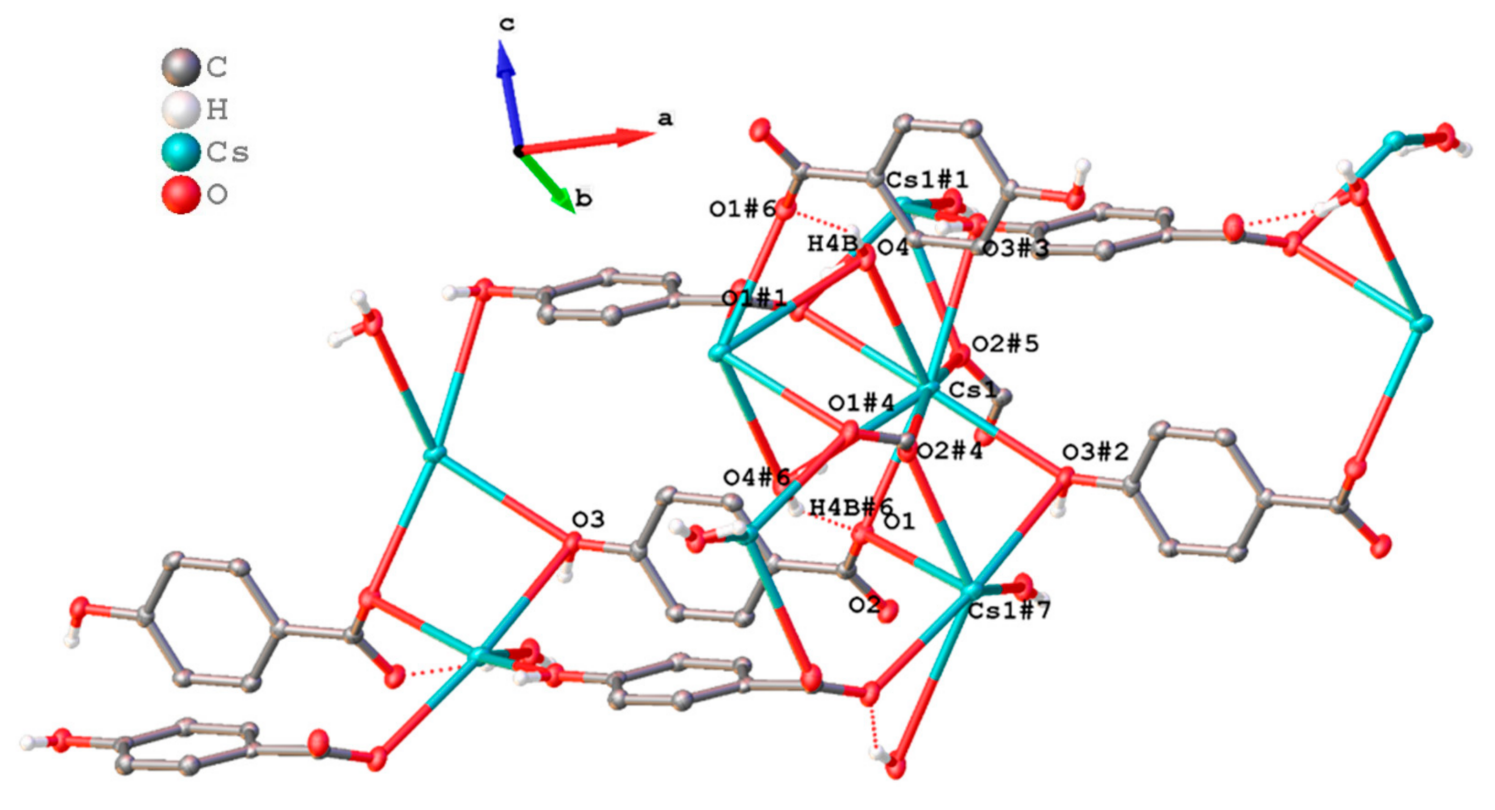
Molecular diagram of [Cs(4hob)(H_2_O)]_n_ **5Cs** indicating the immediate coordination environment around Cs1. Hydrogen atoms, except for those involved in forming H-bonds, are omitted for clarity. (The 1D polymeric chain propagates along the *c*-axis, while a 2D framework forms in the ac-plane. The 2D polymeric chains are linked by alternative four- and eight-membered rings, resulting in the formation of a 3D network along the *b*-axis). Symmetry code: ^#1^ + X,1/2-Y,1/2 + Z; ^#2^ 1 + X, + Y, + Z; ^#3^ 1 + X,1/2-Y,1/2 + Z; ^#4^ 2-X,-1/2 + Y,1/2-Z; ^#5^ 2-X,1-Y,1-Z,; ^#6^ 2-X,-Y,1-Z; ^#7^ + X,1/2-Y,-1/2 + Z.

Additionally, the interactions that join the 1D networks and contribute to the overall 3D structure are similar in both **5Rb** and **5Cs**. However, the **5Cs** structure exhibits a network of H-bonding (Table S12) that holds the 3D network together (Figure 27).

### 2.3. Corrosion Inhibition

#### Immersion Tests

The immersion tests were conducted as an initial screening, lasting for seven days in a 0.01M NaCl control solution. After the completion of the test, digital photographs were taken of the sample coupons (Figure S16). Because the primary objective of the current study was to observe and compare the effectiveness of alkali-metal complexes of various carboxylate derivatives with rare-earth-metal carboxylate corrosion inhibitors, detailed immersion analysis was not pursued for any of the compounds.

Table 3 provides a summary of the weight-loss measurements obtained after 168 h of immersing the samples in specific inhibitor solutions. Upon examination of the results, it was found that the mild-steel coupons immersed in 800 ppm [K(4Hocin)(H_2_O)_3_]_n_ (**4K**) exhibited the highest corrosion inhibition rates, reaching 80%. [K_2_(2m3fur)_2_(H_2_O)]_n_ (**2K**) displayed the lowest inhibition efficiency, with only 9% corrosion inhibition. The remaining tested compounds demonstrated inhibition efficiencies ranging from 25% to 31%.

Previous studies involving 4-hydroxycinnamate as the ligand have indicated that the corrosion rates calculated from the weight-loss data were generally higher for sodium salts compared to their rare earth metal-substituted analogues [18]. In our most recent studies with RE 3-thiophenecarboxylate [14] and 3-furoate [13], we demonstrated the inhibition properties of the rare-earth-metal complexes. By comparing both sets of results (Table 3 and Table 4), it is evident that the corrosion rates of alkali-metal carboxylates on steel are generally much higher than those of rare-earth analogues, indicating the value of rare-earth carboxylates in inhibition.

## 3. Materials and Methods

### 3.1. General Consideration

The experiments were carried out utilising commercially available chemicals and reagents of standard quality, which were used without undergoing any additional purification steps. The elemental Analysis Service Team at the Science Centre, London Metropolitan University, England, performed the elemental analyses. Melting points were determined using glass capillaries, and the values were reported without calibration. To obtain infrared (IR) spectra, a Nicolet™ iS™ 5 FTIR Spectrometer (ThermoFisher Scientific, Waltham, MA, USA) was employed in ATR mode, covering the range of 4000–500 cm^−1^. Powder diffraction patterns were conducted at room temperature using a Bruker D2 PHASER diffractometer (Bruker corporation, Billerica, MA, USA). The measurements were conducted within the range of 2–60, using a 0.2° divergence slit and with increments of 0.02°. To generate X-ray powder simulations, the Mercury program provided by the Cambridge Crystallographic Data Centre [38] was employed. These simulations utilised the single-crystal X-ray diffraction data. Thermogravimetric analysis (TGA) was performed on a TA instrument SDT 650 (TA Instruments, New Castle, DE, USA), using standard 90 µL alumina metal pans. The analysis was conducted at a heating rate of 10 °C/min under a nitrogen atmosphere with a flow rate of 50 mL/min.

### 3.2. X-ray Crystallography

The mounting of single crystals was performed on loops using viscous hydrocarbon oil on the respective diffractometers. For the complexes, **2Li** and **1K** data were collected using the Oxford Diffraction Gemini Ultra dual-source (Mo and Cu) CCD diffractometer collected at 123 K using a Cu source (λ = 1.54184 Å). Data processing was conducted using the CrysAlisPro.55 software suite [39]. Data collection for the rest of the compounds took place at the MX1 beamline located at the Australian Synchrotron. The integration of the data was carried out using Blue-ice [40] and XDS [41] software programs. The structures were solved using SHELXT, and they were refined using full-matrix least-squares methods against F2 with SHELX2018 [42]. The refinement was performed using the Olex2 [43] graphical user interface (GUI). The graphical representations were created using the bitmap image GUI of Olex2 [43]. All hydrogen atoms were placed in calculated positions using the riding model. Detailed information regarding the crystal data and refinement can be found in Table S1.

### 3.3. Synthesis of Alkali-Metal Carboxylate Complexes

General Synthetic Method: Each ligand acid (LH; L = 3tpc, 2m3fur, 3fur, 4hocin, and 4hob) was dissolved in 15 mL of 95% ethanol and neutralised with an equimolar amount of aqueous potassium hydroxide, as shown in Scheme 1 (1), to produce KL compounds. Sodium complexes were also synthesised using the same procedure, employing NaOH instead. For the syntheses of lithium, rubidium, and caesium carboxylate complexes, ligand acid solutions in ethanol (LH) were neutralised with a half-molar amount of aqueous Li_2_CO_3_, Rb_2_CO_3_, and Cs_2_CO_3_, respectively (Scheme 1, (2)). The resulting solutions were stirred at room temperature for 2 h, transferred to separate vials, and left to stand for several days at room temperature, resulting in precipitates. These precipitates were dried over silica gel. Crystals were obtained by the subsequent recrystallisation of the bulk products of **1Li**, **1K**, **1Rb**, **2Li**, **2K** from 95% ethanol and **1Cs**, **3Li**, **4M** (M = K, Rb, Cs) and **5M** (M = Li, K, Rb, Cs) from water/ethanol (95/5 %v/v).

**1Li:** [Li_2_(3tpc)_2_]_n_ white-colour powder. Yield: 50.1%. m.p. >300 °C. Elemental analysis calculated for C_10_H_6_Li_2_O_4_S_2_ (MW: 268.15 gmol^−1^): calculated (%) C: 44.79; H: 2.26; found (%) C: 44.08; H: 2.20. IR (cm^−1^): 3123w, 3105w, 3083w, 1566s, 1514s, 1414s, 1399s, 1352s, 1206m, 119m, 1071m, 935w, 913w, 896w, 869m, 827m, 756s, 694s, 626m, 585m, 540m, 430s.

**1K:** [K_2_(3tpc)_2_]_n_ white-colour powder. Yield: 49.5%. m.p. >300 °C. Elemental analysis calculated for C_10_H_6_K_2_O_4_S_2_ (MW: 332.47 gmol^−1^): calculated (%) C: 36.12; H: 1.82; found (%) C: 36.26; H: 1.77. IR (cm^−1^): 3115w, 3083w, 2358w, 2340w, 1738w, 1614w, 1558s, 1516s, 1412s, 1401s, 1348s, 1204m, 1121m, 1109m, 1067m, 916w, 870m, 849w, 830m, 779m, 753s, 683s, 624m, 574w, 514m, 457w, 442w.

**1Rb:** [Rb(3tpc)(H_2_O)]_n_ white-colour powder. Yield: 90.0%. m.p. >300 °C. Elemental analysis calculated for C_5_H_5_O_3_RbS (MW: 230.62 gmol^−1^): calculated (%) C: 26.04; H: 2.19; found (%) C: 26.54; H: 1.98. IR (cm^−1^): 3348w, 3100w, 3083w, 1723w, 1563s, 1512s, 1405s, 1341s, 1210m, 1199m, 1125m, 1108m, 1065m, 866m, 827m, 760s, 692s, 624m, 553m, 511m, 458w. TGA weight loss (26–110 °C); 7.4% (calc. for loss of 1 × H_2_O = 7.8%).

**1Cs:** [Cs{H(3tpc)_2_}]_n_ white-colour powder. Yield: 20%. m.p. >300 °C. Elemental analysis calculated for C_10_H_7_CsO_4_S_2_ (MW: 388.20 gmol^−1^): calculated (%) C: 30.94; H: 1.82; found (%) C: 30.80; H: 1.73. IR (cm^−1^): 3097w, 1682w, 1636w, 1617w, 1560s, 1510s, 1398s, 1384s, 1342s, 1280w, 1261w, 1197w, 1152w, 1106m, 1063w, 1015w, 994w, 973w, 945w, 926w, 864m, 826m, 765s, 757s, 696s, 670w, 624m, 591w, 557w, 537w, 508m, 439w. TGA weight loss (40–220 °C); 2.5% (calc. for loss of 0.5 × H_2_O (assuming absorbed water = 2.3%)).

**2Li:** [Li_2_(2m3fur)_2_(H_2_O)_3_] white-colour powder. Yield: 51%. m.p. 250 °C (dec.). Elemental analysis calculated for C_12_H_16_Li_2_O_9_ (MW: 318.13 gmol^−1^): calculated (%) C: 45.30; H: 5.07; found (%) C: 44.56; H: 4.68. IR (cm^−1^): 3373m, 3301m, 3128w, 2926w, 1675w, 1598s, 1544m, 1521s, 1458m, 1428s, 1383s, 1361m, 1349m, 1289w, 1248w, 1231w, 1219m, 1138m, 1104s, 1052w, 1035w, 944m, 895m, 852w, 815s, 761w, 729s, 685m, 618s, 599s, 588s, 530s, 500s, 462s. TGA weight loss (50–120 °C); 17.68% (calc. for loss of 3 × H_2_O = 16.97%).

**2K:** [K_2_(2m3fur)_2_(H_2_O)]_n_ white-colour powder. Yield: 50%. m.p. 280 °C (dec.). Elemental analysis calculated for C_12_H_12_K_2_O_7_ (MW: 346.42 gmol^−1^): calculated (%) C: 41.61; H: 3.49; found (%) C: 41.53; H: 3.29. IR (cm^−1^): 3373m, 3301m, 3128w, 2926w, 1675w, 1598s, 1544s, 1521s, 1458m, 1428s, 1383s, 1361s, 1349s, 1289w, 1231w, 1219s, 1138m, 1104s, 1052w, 1035w, 944m, 895m, 815s, 761w, 729s, 685m, 618s, 599s, 588s, 530s, 500s, 462s. TGA weight loss (35–180 °C); 9.4% (calc. for loss of 2 × H_2_O (assuming absorbed water = 9.9%)).

**3Li:** [Li(3fur)]_n_ white-colour powder. Yield: 80%. m.p. 280 °C (dec.). Elemental analysis calculated for C_5_H_3_LiO_3_ (MW: 118.01 gmol^−1^): calculated (%) C: 50.88; H: 2.56; found (%) C: 50.78; H: 2.50. IR (cm^−1^): 3154w, 3132w, 2298w, 1563s, 1552s, 1505s, 1428s, 1368m, 1251w, 1213m, 1149m, 1069m, 1004m, 970mw, 873m, 780s, 752s, 606m, 595m, 553m, 468s.

**4K:** [K(4hocin)(H_2_O)_3_]_n_ white-colour powder. Yield: 69%. m.p. 200 °C (dec.). Elemental analysis calculated for C_9_H_13_KO_6_ (MW: 256.29 gmol^−1^): calculated (%) C: 42.18; H: 5.11; C_9_H_10_KO_4.5_ (MW: 229.27 gmol^−1^, loss of 1.5 H_2_O): calculated (%) C: 47.15; H: 4.40; found (%) C: 46.51; H: 4.27. IR (cm^−1^): 3444m, 3023w, 2815w, 1636m, 1607w, 1590m, 1531w, 1511s, 1454w, 1371s, 1289m, 1270m, 1237s, 1167m, 1102m, 1013w, 973s, 936w, 874w, 829s, 802m, 697m, 641w, 516s, 465s, 452s, 460m. TGA weight loss (25–90 °C); 20.3% (calc. for loss of 3 × H_2_O = 21.1%).

**4Rb:** [Rb{H(4hocin)_2_}]_n·_nH_2_O white-colour powder. Yield: 59%. m.p. 200 °C (dec.). Elemental analysis calculated for C_18_H_17_O_7_Rb (MW: 430.79 gmol^−1^): calculated (%) C: 50.19; H: 3.98; C_11.25_H_11_O_4.75_Rb (MW: 307.67 gmol^−1^, loss of 0.75 4hocin): calculated (%) C: 43.92; H: 3.60; found (%) C: 44.19; H: 3.67. IR (cm^−1^): 3287w, 2624m, 1634m, 1608w, 1591m, 1537w, 1514s, 1454s, 1381s, 1288w, 1243s, 1198w, 1171m, 1103m, 1010w, 979s, 939m, 900m, 864m, 828s, 804s, 748w, 710m, 628w, 551s, 525s, 515s, 458m. TGA weight loss (50–115 °C); 6.0% (calc. for loss of 1 × H_2_O from {[Rb(4hocin)(4hocinH)_0.25_] (H_2_O)}_n_ = 5.9%).

**4Cs:** [Cs(4hocin)(H_2_O)]_n_ white-colour powder. Yield: 86%. m.p. 200 °C (dec.). Elemental analysis calculated for C_9_H_9_CsO_4_ (MW: 314.07 gmol^−1^): calculated (%) C: 34.53; H: 2.58; found (%) C: 34.41; H: 2.90. IR (cm^−1^): 3212m, 2999m, 2808m, 2687m, 1887w, 1633m, 1607m, 1588m, 1535w, 1514s, 1453s, 1379s, 1287w, 1243s, 1167m, 1099m, 1010w, 978s, 938w, 878m, 861w, 827s, 802m, 748w, 710m, 661w, 641w, 549s, 511s, 459m. TGA weight loss (50–120 °C); 5.4% (calc. for loss of 1 × H_2_O = 5.7%).

**5Li:** [Li(4hob)]_n_ white-colour powder. Yield: 72%. m.p. 270 °C (dec.). Elemental analysis calculated for C_7_H_5_LiO_3_ (MW: 144.05 gmol^−1^): calculated (%) C: 58.37; H: 3.50; C_7_H_5.5_LiO_3.25_(MW: 148.56 gmol^−1^, absorbed 0.25 H_2_O): calculated (%) C: 56.59; H: 3.73; found (%) C: 56.37; H: 3.36. IR (cm^−1^): 3357m, 1602s, 1556, 1399s, 1360m, 1238s, 1168s, 1143m, 1100s, 1011m, 978w, 858m, 841w, 805m, 787s, 701m, 638w, 619s, 548m, 518m, 503w, 450s, 425s.

**5K:** [K(4hob)(H_2_O)_3_]_n_ white-colour powder. Yield: 83%. m.p. 260 °C (dec.). Elemental analysis calculated for C_7_H_11_KO_6_ (MW: 230.26 gmol^−1^): calculated (%) C: 36.51; H: 4.81; C_7_H_7_KO_4_ (MW: 194.23 gmol^−1^,loss of 2 H_2_O): calculated (%) C: 43.29; H: 3.63; found (%) C: 43.84; H: 3.38. IR (cm^−1^): 3437s, 3175s, 2689w, 1635w, 1599s, 1533s, 1501m, 1438w, 1361s, 1297w, 1268m, 1248s, 1163s, 1138m, 1100s, 1008w, 953w, 854s, 787s, 776s, 695s, 614s, 530m, 470s, 432s. TGA weight loss (27–150 °C); 21.2% (calc. for loss of 3 × H_2_O = 23.4%).

**5Rb:** [Rb(4hob)(H_2_O)]_n_ white-colour powder. Yield: 56%. m.p. 270 °C (dec.). Elemental analysis calculated for C_7_H_7_O_4_Rb (MW: 240.60 gmol^−1^): calculated (%) C: 34.94; H: 2.93; found (%) C: 34.92; H: 2.77. IR (cm^−1^): 3300w, 3018m, 2890m, 2798m, 2694m, 1914w, 1694w, 1597s, 1530s, 1510s, 1462m, 1380s, 1252s, 1165s, 1142m, 1100s,1011w, 967w, 849s, 834s, 816w 784s, 704s, 614s, 549s, 496s. TGA weight loss (100–140 °C); 7.5% (calc. for loss of 1 × H_2_O = 7.5%).

**5Cs:** [Cs(4hob)(H_2_O)]_n_ white-colour powder. Yield: 91%. m.p. 270 °C (dec.). Elemental analysis calculated for C_7_H_7_CsO_4_ (MW: 288.04 gmol^−1^): calculated (%) C: 29.19; H: 2.45; found (%) C: 29.39; H: 2.41. IR (cm^−1^): 3194m, 3036w, 2809w, 2675w, 2522w, 2114w, 1660s, 1607s, 1591s, 1512w, 1440m, 1377m, 1355m, 1316w, 1267m, 1227s, 1162s, 1099s, 925m, 849s, 768s, 697s, 666m, 638m, 612s, 546m, 522w, 498s. TGA weight loss (75–130 °C); 5.3% (calc. for loss of 1 × H_2_O = 6.2%)

### 3.4. Corrosion Testing

In order to assess the general corrosion and inhibition characteristics of the synthesised compounds, corrosion immersion experiments were conducted following the standard method ASTM G31-72 [44]. To conduct weight-loss tests, mild-steel alloy AS 1020 coupons were prepared by cutting them into approximately 20 × 20 × 1.5 mm dimensions and progressively abrading them using sanding sheets of grits ranging from 80 to 2000. The specimens were subsequently rinsed with distilled water, followed by ethanol, and dried under flowing N_2_ gas. These coupons were immediately used for a series of immersion tests lasting up to 168 h (7 days). Both the sample and the control coupons were placed in separate beakers containing 0.01 M NaCl solutions, with and without the presence of 800 ppm of the inhibitor compounds. In each setup, coupons were fully immersed at mid-depth using Teflon strings. After completing the tests, the corrosion product adhering to the substrate was initially removed by subjecting it to mild sonication in clean, distilled water. Subsequently, the finest sanding papers were used with minimum force to prevent the removal of intact material. The experiments were carried out in duplicate to ensure reliability. Finally, the coupons were washed with ethanol and dried using N_2_ gas. The corrosion rates (Rs) of the inhibitor solutions were determined by applying Equation (3), outlined below:

(3) $$\mathrm{Corrosion}\;\mathrm{rate}\;(R)=\frac{KW}{ATD}$$

where *K* is the A constant; *W* is the weight loss (g); *A* is the coupon area (cm^2^); *T* is the time of exposure (168 h); *D* is the density of the test metal. 

The percentage corrosion inhibition (η) was determined using Equation (4):

(4) $$\eta =\frac{\;R\left(Control\right)-R\;\left(inhibitor\right)}{R\left(Control\right)}\;\times \;100$$

## 4. Conclusions

Fourteen new complexes of the alkali metals with 3-thiophenecarboxylate (3tpc), 2-methyl-3-furoate (2m3fur), 3-furoate (3fur), 4-hydroxycinnamate (4hocin), and 4-hydroxybenzoate (4hob) were synthesised. The preparations gave moderate yields of crystalline materials, and the complexes were characterised via IR spectroscopy, powder XRD, TGA, and microanalyses. Furthermore, X-ray crystallographic examination of the single crystals of all the compounds revealed unique structures for each of them. In the series of compounds, only the [Li_2_(2m3fur)_2_(H_2_O)_3_] **2Li** complex crystallised as a coordination dinuclear complex with four-coordinate lithium atoms. In contrast, all other complexes were found to be polymeric, with varying coordination numbers and stereochemistry. Compounds **1Cs** and **4Rb** incorporate [HL_2_]^−^ ligands rather than simple carboxylate groups and exhibit centrosymmetric O...H...O interactions between the carboxylate ions.

To investigate the potential application of the complexes as corrosion inhibitors, weight-loss measurements were conducted, but the compounds were less effective than their rare-earth counterparts.

## Figures and Tables

**Table 1:** Selected IR bands of alkali-metal 3tpc, 2m3fur, 3fur, 4hocin, and 4hob complexes.

Compounds	Significant Bands (cm^−1^)				
	ν(OH)	n(C=C) Propenyl	ν_as_(CO_2_^−^)	ν_s_(CO_2_^−^)	Δν ^a^
[Li_2_(3tpc)_2_]_n_ **1Li**	-	-	1566,1514	1414,1399	133.5
[K_2_(3tpc)_2_]_n_ **1K**	-	-	1558,1516	1412,1401	130.5
[Rb(3tpc)(H_2_O)]_n_ **1Rb**	3348	-	1563,1512	1405	132.5
[Cs{H(3tpc)_2_}]_n_ **1Cs**	-	-	1560,1510	1398,1384	144
[Li_2_(2m3fur)_2_(H_2_O)_3_] **2Li**	3301,3373	-	1544,1521	1428	104.5
[K_2_(2m3fur)_2_(H_2_O)]_n_ **2K**	3354	-	1532,1517	1418	106.5
[Li(3fur)]_n_ **3Li**	-	-	1552	1428	124
[K(4hocin)(H_2_O)_3_]_n_ **4K**	3114,3444	1636	1531,1511	1371	150
[Rb{H(4hocin)_2_}]_n·_nH_2_O **4Rb**	3241	1634	1537,1514	1381	144.5
[Cs(4hocin)(H_2_O)]_n_ **4Cs**	3200	1633	1537,1514	1378	147.5
[Li(4hob)]_n_ **5Li**	3351	-	1556	1399	157
[K(4hob)(H_2_O)_3_]_n_ **5K**	3437	-	1533	1361	172
[Rb(4hob)(H_2_O)]_n_ **5Rb**	3317	-	1529,1509	1381	138
[Cs(4hob)(H_2_O)]_n_ **5Cs**	3202	-	1529,1509	1376	143

^a^ Δν = ν_as_(CO_2_)—ν_s_(CO_2_).

**Table 2:** Crystallisation conditions, geometry, coordination number, carboxylate ligation, and average M-O bond distance for alkali-metal 3tpc, 2m3fur, 3fur, 4hocin, and 4hob complexes.

Compound	M Atom	Geometry	Coordination Number	Ligation	Avg M-O (Å)
**1Li**	Li1 Li2	Tetrahedral Tetrahedral	4 4	4 × (i) 4 × (i)	1.963 1.963
**1K**	K1 K2	Irregular polyhedron Irregular polyhedron	5 5	1 × (ii) ^a^; 3 × (ii) ^b^ 1 × (ii) ^a^; 3 × (ii) ^b^	2.791 2.767
**1Rb**	Rb1	Distorted capped trigonal prismatic	7	3 × (iii); 4 × µ_4_-H_2_O	2.995
**1Cs**	Cs1	Cubic	8	8 × (i)	3.152
**2Li**	Li1	Tetrahedral	4	2 × (iv); 1 × H_2_O; 2 × µ_2_-H_2_O	1.957
**2K**	K1	Distorted pseudo-pentagonal bipyramidal	8	1 × (ii) ^a^; 3 × (v) ^b^; 1 × (ii) ^b^; 2 × µ_3_-H_2_O	2.841
	K2	Distorted pseudo-square pyramidal	6	1 × (v) ^a^; 2 × (ii) ^b^; 1 × (v) ^b^; 1 × µ_3_-H_2_O	2.750
**3Li**	Li1	Tetrahedral	4	4 × (i)	1.978
**4K**	K1	Distorted tricapped trigonal prismatic	9	2 × (vi); 3 × µ_3_-H_2_O; 2 × µ_2_-H_2_O; 2 × µ_2_-H_2_O;	2.977
**4Rb**	Rb1	Irregular polyhedron	6	4 × (vii); 2 × phenolic O	2.932
**4Cs**	Cs1	Irregular polyhedron	8	1 × (viii) ^a^; 2 × (viii) ^b^; 2 × µ_2_-H_2_O; 2 × phenolic O	3.349
**5Li**	Li1	Tetrahedral	4	4 × (i)	1.953
**5K**	K1	Square antiprismatic	8	2 × (vi); 1 × H_2_O; 3 × µ_3_-H_2_O; 2 × µ_2_-H_2_O	2.912
**5Rb**	Rb1	Square antiprismatic	8	4 × (i); 2 × µ_2_-H_2_O; 2 × phenolic O	3.049
**5Cs**	Cs1	Square antiprismatic	8	4 × (i); 2 × µ_2_-H_2_O; 2 × phenolic O	3.217

^a^ Chelating; ^b^ unidentate.

**Table 3:** Observed weight loss, corrosion rates (μgm^−2^s^−1^), and percentage inhibition (η) for mild steel. Coupons immersed in specific solutions at 800 ppm concentrations in 0.01 M NaCl for seven days.

Solution	Concentration		Corrosion Rate (μgm^−2^s^−1^)	% Inhibition (η)
	ppm	mM		
Control—NaCl	580	10	39.1	-
[Li_2_(3tpc)_2_]_n_ (**1Li**)	800	2.98	27.1	31
[K_2_(3tpc)_2_]_n_ (**1K**)	800	2.41	28.8	26
[Rb(3tpc)(H_2_O)]_n_ (**1Rb**)	800	3.47	27.4	30
[K_2_(2m3fur)_2_(H_2_O)]_n_ (**2K**)	800	2.31	35.7	9
[Li(3fur)]_n_ (**3Li**)	800	6.78	29.2	25
[K(4hocin)(H_2_O)_3_]_n_ (**4K**)	800	3.12	7.5	80
[K(4hob)(H_2_O)_3_]_n_ (**5K**)	800	3.47	29.7	24

**Table 4:** Anticorrosion data for some corresponding rare-earth carboxylates.

Ligand	Solutions	Concentration		Corrosion Rate (μgm^−2^s^−1^)	% Inhibition (η)	Ref
		ppm	mM			
**1**-3tpc	[La(3tpc)_3_(H_2_O)_3_]_n_	500	0.871	19.4	36	[14]
**1**-3tpc	[Ce(3tpc)_3_(H_2_O)_3_]_n_	500	0.869	18.8	38	[14]
**1**-3tpc	[Y_2_(3tpc)_6_(H_2_O)_4_]. H_2_O	500	0.485	9.65	68	[14]
**3**-3fur	[La(3fur)_3_(H_2_O)_2_]_n_	800	1.57	5.14	87	[13]
**3**-3fur	[Ce(3fur)_3_(H_2_O)_2_]_n_	800	1.57	8.3	79	[13]
**3**-3fur	[Y(3fur)_3_(H_2_O)_2_]_n_	800	1.75	3.9	90	[13]
**4**-4ohcin	La(4hocin)_3_	500	0.7	3.2	91	[18]
**4**-4ohcin	Ce(4hocin)_3_	200	0.28	5.7	84	[18]

## Data Availability

Crystal data can be obtained free of charge from The Cambridge Crystallographic Data Centre (CCDC 2268145-2268148 for complexes **1M**, 2268149-2268150 for complexes **2M**, 2268151 for complex 3**M**, 2268152-2268154 for complexes **4M**, and 2268155-2268158 for complexes **5M**).

## Supplementary Materials

The following supporting information can be downloaded at https://www.mdpi.com/article/10.3390/molecules28145515/s1, Figure S1: TGA plots of [M(3tpc)]_n_ **1M** series (M = Na, Li, K, Rb, Cs); Figure S2: PXRD traces of [M(3tpc)]_n_ (M = Na, Li, K, Rb, Cs) **1M** series at room temperature compared to their simulated patterns; Figure S3: IR spectra of [M(3tpc)]_n_ **1M** series (M = Na, Li, K, Rb, Cs); Figure S4: TGA plots of [M(2m3fur)]_n_ **2M** series (M = Na, Li, K); Figure S5: PXRD traces of [M(2m3fur)]_n_ **2M** series (M = Na, Li, K) at room temperature compared to their simulated patterns; Figure S6: IR spectra of [M(2m3fur)]_n_ **2M** series (M = Na, Li, K); Figure S7: TGA plots of [Na3fur] and [Li(3fur)]_n_ (**3Li**); Figure S8: PXRD traces of [Na3fur] and [Li(3fur)]_n_ (**3Li**) at room temperature compared to their simulated patterns; Figure S9: IR spectra of [Na3fur] and [Li(3fur)]_n_ (**3Li**); Figure S10: TGA plots of [M(4hocin)]_n_ **4M** series (M = Na, K, Rb, Cs); Figure S11: PXRD traces of [M(4hocin)]_n_ **4M** series (M = Na, K, Rb, Cs) at room temperature compared to their simulated patterns; Figure S12: IR spectra of [M(4hocin)]n **4M** series (M = Na, K, Rb, Cs); Figure S13: TGA plots of [M(4hob)]_n_ **5M** series (M = Na, Li, K, Rb, Cs); Figure S14: PXRD traces of [M(4hob)]_n_ **5M** series (M = Na, Li, K, Rb, Cs) series at room temperature compared to their simulated patterns; Figure S15: IR spectra of [M(4hob)]n **5M** series (M = Na, Li, K, Rb, Cs); Figure S16: Mild-steel coupons immersed in control and the inhibited solutions for 168 h (left—Trial 1; right—Trial 2.); Table S1: Crystal data and structural refinement for the alkali-metal carboxylate complexes; Table S2: Selected bond lengths and M…M distances (Å) for [M(3tpc)]_n_ **1M** series (M = Li, K, Rb, Cs); Table S3: Selected bond angles (°) for [M(3tpc)]_n_ **1M** series (M = Li, K, Rb); Table S4: Selected bond lengths and M…M distances (Å) for [M(2m3fur)]_n_ **2M** series (M = Li, K); Table S5: Selected bond angles (°) for [M(2m3fur)]_n_ **2M** series (M = Li, K); Table S6: Selected bond lengths and M…M distances (Å) for [Li(3fur)]_n_ (**3Li**); Table S7: Selected bond angles (°) for [Li(3fur)]_n_ (**3Li**); Table S8: Selected bond lengths and M…M distances (Å) for [M(4hocin)]_n_ **4M** series (M = K, Rb, Cs); Table S9: Selected bond angles (°) for [M(4hocin)]_n_ **4M** series (M = K, Rb, Cs); Table S10: Selected bond lengths and M…M distances (Å) for [M(4hob)]_n_ **5M** series (M = Li, K, Rb, Cs); Table S11: Selected bond angles (°) for [M(4hob)]_n_ **5M** series (M = Li, K, Rb, Cs); Table S12: Hydrogen-bonding parameters [d/Å and </°] for the compounds **1Rb**, **4K**, **4Rb**, **4Cs**, **5K**, **5Cs**.
