# Supplementary material for: Syntheses, Structures, and Corrosion Inhibition of Various Alkali Metal Carboxylate Complexes
Source: Molecules. 2023 Jul 19;28(14):5515. doi: 10.3390/molecules28145515 (PMC10385946; doi:10.3390/molecules28145515)
Supplement: Supplementary file 1 [file molecules-28-05515-s001.zip › molecules-2515692-supplementary.pdf]

Supplementary Material

Content:

1. **Table S1:** Crystal data and structural refinement for the alkali metal carboxylate complexes
2. **Table S2:** Selected bond lengths and M…M distances (Å) for $[M(3tpc)]_n$ **1M** series (M=Li, K, Rb, Cs).
3. **Table S3.** Selected bond angles (°) for $[M(3tpc)]_n$ **1M** series (M=Li, K, Rb).
4. **Table S4.** Selected bond lengths and M…M distances (Å) for $[M(2m3fur)]_n$ **2M** series (M=Li, K).
5. **Table S5.** Selected bond angles (°) for $[M(2m3fur)]_n$ **2M** series (M=Li, K).
6. **Table S6.** Selected bond lengths and M…M distances (Å) for $[Li(3fur)]_n$ (**3Li**).
7. **Table S7.** Selected bond angles (°) for $[Li(3fur)]_n$ (**3Li**.).
8. **Table S8.** Selected bond lengths and M…M distances (Å) for $[M(4hocin)]_n$ **4M** series (M=K, Rb, Cs).
9. **Table S9.** Selected bond angles (°) for the $[M(4hocin)]_n$ **4M** series (M=K, Rb, Cs).
10. **Table S10.** Selected bond lengths and M…M distances (Å) for $[M(4hob)]_n$ **5M** series (M=Li, K, Rb, Cs).
11. **Table S11.** Selected bond angles (°) for the $[M(4hob)]_n$ **5M** series (M=Li, K, Rb, Cs).
12. **Table S12.** Hydrogen bonding Parameters [d/Å and </°] for the compounds **1Rb**, **4K**, **4Rb, 4Cs, 5K, 5Cs**
13. **Figure S1.** TGA plots of $[M(3tpc)]_n$ **1M** series (M=Na, Li, K, Rb, Cs)
14. **Figure S2.** PXRD traces of $[M(3tpc)]_n$ (M=Na, Li, K, Rb, Cs) **1M** series at room temperature compared to their simulated patterns
15. **Figure S3.** IR spectra of $[M(3tpc)]_n$ **1M** series (M=Na, Li, K, Rb, Cs)
16. **Figure S4.** TGA plots of $[M(2m3fur)]_n$ **2M** series (M=Na, Li, K)
17. **Figure S5.** PXRD traces of $[M(2m3fur)]_n$ **2M** series (M=Na, Li, K) series at room temperature compared to their simulated patterns
18. **Figure S6.** IR spectra of $[M(2m3fur)]_n$ **2M** series (M=Na, Li, K)
19. **Figure S7.** TGA plots of [Na3fur] and $[Li(3fur)]_n$ **(3Li)**
20. **Figure S8.** PXRD traces of [Na3fur] and $[Li(3fur)]_n$ **(3Li)** at room temperature compared to the simulated pattern of **3Li**
21. **Figure S9.** IR spectra of [Na3fur] and $[Li(3fur)]_n$ **(3Li)**
22. **Figure S10.** TGA plots of $[M(4hocin)]_n$ **4M** series (M=Na, K, Rb, Cs).
23. **Figure S11.** PXRD traces of $[M(4hocin)]_n$ **4M** series (M=Na, K, Rb, Cs) series at room temperature compared to their simulated patterns
24. **Figure S12.** IR spectra of $[M(4hocin)]_n$ **4M** series (M=Na, K, Rb, Cs).
25. **Figure S13.** TGA plots of $[M(4hob)]_n$ **5M** series (M=Na, Li, K, Rb, Cs).
26. **Figure S14.** PXRD traces of $[M(4hob)]_n$ **5M** series (M=Na, Li, K, Rb, Cs) series at room temperature compared to their simulated patterns
27. **Figure S15.** IR spectra of $[M(4hob)]_n$ **5M** series (M=Na, Li, K, Rb, Cs).
28. **Figure S16.** Mild steel coupons immersed in control and the inhibited solutions for 168 h (Left – Trial 1; right – Trial 2.)

	**Table S1.** Crystal data and structural refinement for alkali metal carboxylate complexes						
	**1Li**	**1K**	**1Rb**	**1Cs**	**2Li**	**2K**	**3Li**
	**$[Li_2(3tpc)_2]_n$**	**$[K_2(3tpc)_2]_n$**	**$[Rb(3tpc)(H_2O)]_n$**	**$[Cs\{H(3tpc)_2\}]_n$**	**$[Li_2(2m3fur)_2(H_2O)_3]$**	**$[K_2(2m3fur)_2(H_2O)]_n$**	**$[Li(3fur)]_n$**
Formula	$C_{10}H_6Li_2O_4S_2$	$C_{10}H_6K_2O_4S_2$	$C_5H_5O_3RbS$	$C_{10}H_7CsO_4S_2$	$C_{12}H_{16}Li_2O_9$	$C_{12}H_{12}K_2O_7$	$C_5H_3LiO_3$
M_r	268.15	332.47	230.62	388.19	318.13	346.42	118.01
Space group	$Pna2_1$	$Pca2_1$	$P2_1/c$	$C2/c$	$Pbcn$	$P2_1/c$	$P2_1/c$
a (Å)	8.2726(2)	11.2493(2)	16.150(3)	28.169(6)	7.2566(15)	13.830(3)	11.110(2)
b (Å)	5.2114(1)	3.97510(10)	3.9200(8)	3.9620(8)	8.3455(17)	7.4700(15)	5.3200(11)
c (Å)	26.1699(6)	28.4846(5)	11.610(2)	11.582(2)	25.301(5)	14.050(3)	8.2900(17)
α (°)	90	90	90	90	90	90	90
β (°)	90	90	95.06(3)	106.22(3)	90	91.50(3)	90.43(3)
γ (°)	90	90	90	90	90	90	90
V (Å^3)	1128.23(4)	1273.75(5)	732.1(3)	1241.2(5)	1532.3(5)	1451.0(5)	489.97(17)
Z	4	4	4	4	4	4	4
ρ_{calc}, g cm^{-3}	1.579	1.734	2.092	2.077	1.379	1.586	1.600
μ, mm^{-1}	4.279	9.691	6.988	3.317	1.002	0.682	0.130
N_τ	7684	13706	15745	6572	4655	22578	7697
N (R_{int})	1675 (0.0397)	1995(0.0819)	1295(0.0584)	1062(0.0993)	1499(0.0335)	2553(0.0640)	862(0.0534)
$R_1(I > 2\sigma(I))$	0.0334	0.0552	0.0299	0.0427	0.0383	0.0287	0.0407
wR_2 (all data)	0.0858	0.1419	0.0829	0.1014	0.1022	0.0921	0.0989
GOF	1.112	1.059	1.095	1.094	1.056	1.144	1.113

	4K	4Rb	4Cs	5Li	5K	5Rb	5Cs
	**$[K(4hocin)(H_2O)]_3]_n$**	**$[Rb[H(4hocin)_2]]_n \cdot H_2O$**	**$[Cs(4hocin)(H_2O)]_n$**	**$[Li(4hob)]_n$**	**$[K(4hob)(H_2O)_3]_n$**	**$[Rb(4hob)(H_2O)]_n$**	**$[Cs(4hob)(H_2O)]_n$**
Formula	$C_9H_{13}KO_6$	$C_{18}H_{17}O_7Rb$	$C_9H_9CsO_4$	$C_7H_5LiO_3$	$C_7H_{11}KO_6$	$C_7H_7O_4Rb$	$C_7H_7CsO_4$
M_r	256.29	430.79	314.07	144.05	230.26	240.60	288.04
Space group	$P2_1/c$	$C2/c$	$P2_1$	$P2_1/c$	$P2_1/c$	$P2_1/c$	$P2_1/c$
a (å)	14.430(3)	21.901(4)	4.3870(9)	14.904(3)	12.343(3)	10.107(2)	10.120(2)
b (å)	11.130(2)	10.628(2)	9.882(2)	5.0420(10)	11.252(2)	10.007(2)	10.100(2)
c (å)	7.0500(14)	7.3180(15)	12.131(2)	8.4750(17)	7.0700(14)	8.0180(16)	8.6200(17)
α (°)	90	90	90	90	90	90	90
β (°)	98.04(3)	97.94(3)	98.13(3)	99.38(3)	102.18(3)	98.55(3)	102.88(3)
γ (°)	90	90	90	90	90	90	90
V (å^3)	1121.1(4)	1687.0(6)	520.62(19)	628.3(2)	989.8(4)	801.9(3)	858.(9)
Z	4	4	2	4	4	4	4
ρ_{calc}, g cm^{-3}	1.518	1.696	2.003	1.523	1.593	1.993	2.227
μ, mm^{-1}	0.484	2.975	3.542	0.117	0.555	6.143	4.282
N_τ	24948	9183	5565	1062	10907	8922	18355
N (R_{int})	1979(0.0649)	1437(0.0292)	1750(0.0694)	1062(twinned)	1679(0.0269)	1377(0.0237)	1471(0.0501)
$R_1(I > 2\sigma(I))$	0.0351	0.0223	0.0328	0.0334	0.0253	0.0148	0.0189
wR_2 (all data)	0.0963	0.0580	0.0720	0.0950	0.0704	0.0412	0.0498
GOF	1.092	1.190	1.147	1.056	1.093	1.116	1.123

Table S2. Selected bond lengths and M…M distances (Å) for $[M(3tpc)]_n$ **1M** series (M=Li, K, Rb, Cs).

**1Li**		**1K**		**1Rb**		**1Cs**	
**Atoms**	**Bond Lengths**	**Atoms**	**Bond Lengths**	**Atoms**	**Bond Lengths**	**Atoms**	**Bond Lengths**
Li1- O1	1.958(8)	K1-O1	2.730(6)	Rb1-O1	2.828(2)	Cs1-O1	3.025(4)
Li1-O3	1.924(8)	K1-O2	3.155(7)	Rb1-O3	3.000(2)	Cs1-O1#3	3.025(4)
Li1-O4#1	1.982(8)	K1-O3	2.662(6)	Rb1-O1#2	2.846(2)	Cs1-O1#1	3.128(4)
Li1-O2#2	1.989(8)	K1-O4#4	2.662(6)	Rb1-O2#6	2.920(2)	Cs1-O1#4	3.128(3)
Li2-O2	1.934(8)	K1-O3#2	2.744(6)	Rb1-O3#8	3.278(3)	Cs1-O2#5	3.304(3)
Li2-O4#4	1.966(8)	K2-O2	2.688(6)	Rb1-O3#4	3.043(2)	Cs1-O2#8	3.304(3)
Li2-O3#3	2.004(8)	K2-O3	2.673(6)	Rb1-O3#7	3.049(3)	Cs1-O2#7	3.151(3)
Li2-O1#4	1.947(8)	K2-O4	3.117(6)	Rb1…Rb1#1	5.1213(12)	Cs1-O2#6	3.151(3)
Li1…Li2	3.146(11)	K2-O2#1	2.722(5)	Rb1#7…Rb1	4.8220(13)	Cs1…Cs1#2	3.962(8)
Li1…Li2#1	3.116(10)	K2-O1#6	2.635(6)				
Li2…Li1#4	3.116(10)	K1…K2	4.208(2)				
Li1…Li2#2	2.712(9)	K1…K2#4	4.108(2)				
Li2…Li1#3	2.712(9)	K2…K1#6	4.058(2)				
		K1…K1#1	3.9751(1)				
		K2…K2#1	3.9751(1)				

Table S3. Selected bond angles (°) for $[M(3tpc)]_n$ **1M** series (M=Li, K, Rb).

**1Li**		**1K**		**1Rb**	
Li1-O1-C1	139.3(4)	K1#6…K2…K1	85.85(3)	Rb1#7…Rb1…Rb1#1	51.910(19)
Li2-O2-C1	115.9(3)	K2…K1…K2#4	85.15(2)	O2#8-Rb1-O3#4	71.00(6)
Li1-O4#4-Li2	105.6(4)			O3#8-Rb1-O3	71.52(6)
Li1-O3-C6	138.7(4)°			O3#8-Rb1-O3#4	71.00(6)
Li2#1-O4-C6	115.6(3)°			O3#8-Rb1-O3#7	133.11(8)
				O3-Rb1-O3#7	74.28(7)
				O3-Rb1-O3#4	80.89(6)
				O3#7-Rb1-O3#4	74.74(7)

Table S4. Selected bond lengths and M…M distances (Å) for $[M(2m3fur)]_n$ **2M** series (M=Li, K).

2Li		2K	
**Atoms**	**Bond Lengths**	**Atoms**	**Bond Lengths**
Li1- O1	1.910(3)	K1-O1	2.7019(12)
Li1-O4	1.893(3)	K1-O4	2.7397(12)
Li1-O5	2.034(3)	K1-O7#2	2.8308(12)
Li1-O2#1	1.992(3)	K1-O1#5	2.7025(12)
Li1…Li1#1	2.758(5)	K1-O4#5	2.8618(12)
		K1-O5#5	3.0462(13)
		K1-O7#6	2.8843(12)
		K1-O2#4	2.9599(13)
		K2-O7	2.6942(12)
		K2-O5	2.6548(12)
		K2-O1#1	2.8213(13)
		K2-O2#1	2.8517(14)
		K2-O4#1	2.6833(12)
		K2-O2#3	2.7962(12)
		K1…K2	6.1085(12)
		K1…K2#7	3.6743(8)

Table S5. Selected bond angles (°) for $[M(2m3fur)]_n$ **2M** series (M=Li, K).

2Li		2K		2K (Cont.)	
O1-Li1-O2#1	123.49(14)	O4#5-C7#5-O5#5	124.09(14)	O5-K2-O4#1	153.67(4)
O1#1-Li1#1-O2	123.49(14)	O1#1-C1#1-O2#1	124.46(14)		
Li1-O5-Li1#1	85.41(15)	O7#2-K1-O7#6	148.20(3)		
O4-Li-O5	116.51(13)	O4-C7-O5	124.09(14)		
O4#1-Li1#1-O5	116.51(13)	O4-K1-C7#5	168.53(4)		
O1-C6-O2	123.14(12)	O7-K2-O2#3	152.91(4)		

Table S6. Selected bond lengths and M…M distances (Å) for $[Li(3fur)]_n$ (**3Li**).

3Li	
**Atoms**	**Bond Lengths**
Li1- O1	1.935(3)
Li1-O1#3	2.025(3)
Li1-O2#5	1.996(3)
Li1-O2#4	1.954(3)
Li1…Li1#1	3.155(3)
Li1…Li1#4	3.155(3)
Li1…Li1#3	2.758(5)

Table S7. Selected bond angles (°) for $[Li(3fur)]_n$ (**3Li**.).

3Li	
Li1#1…Li1…Li1#4	114.93(16)
C1-O1-Li1	141.84(12)
C1-O2-Li1#1	116.97(12)
Li1-O2#4-Li1#1	106.05(9)
Li1-O2#5-Li1#4	106.05(9)
Li1#4-O1#4-C1#4	141.84(12)

Table S8. Selected bond lengths and M…M distances (Å) for $[M(4hocin)]_n$ **4M** series (M=K, Rb, Cs).

4K		4Rb		4Cs	
**Atoms**	**Bond Lengths**	**Atoms**	**Bond Lengths**	**Atoms**	**Bond Lengths**
K1-O1	2.7658(14)	Rb1-O1	2.8868(12)	Cs1-O1#1	3.138(5)
K1-O1#1	2.9157(16)	Rb1-O1#5	2.8868(12)	Cs1-O1	3.020(5)
K1-O4	2.8052(13)	Rb1-O2#3	2.9685(13)	Cs1-O2#1	3.788(6)
K1-O4#1	3.1099(14)	Rb1-O2#4	2.9685(13)	Cs1-O2#2	3.059(6)
K1-O4#3	2.9297 (13)	Rb1-O3#1	2.9396(12)	Cs1-O3#4	3.386(5)
K1-O5	3.3762(14)	Rb1-O3#2	2.9396(12)	Cs1-O3#5	3.3069(5)
K1-O5#3	3.3087(15)			Cs1-O4	3.310(5)
K1-O6	2.7326(15)			Cs1-O4#3	3.788(5)

K1-O6#1	2.8497(16)			Cs1…Cs1#6	4.3870(9)
K1…K1#1	3.9478(7)				
K1…K1#3	4.0171(9)				
O4-K1-O4#1	132.96(4)				
O4-K1-O4#3	91.10(4)				
O4#1-K1-O4#3	135.77(17)				

Table S9. Selected bond angles (°) for $[M(4hocin)]_n$ **4M** series (M=K, Rb, Cs).

**4K**		**4K(cont.)**		**4Rb**		**4CS**	
K1#1…K1…K1#2	126.48(2)	O5-K1-O5#3	106.13(3)	O3#1-Rb1-O3#2	67.40(5)	O4-Cs1-O4#3	138.84(10)
O4-K1-O5	70.01(4)	O6-K1-O4#3	82.61(4)	O3-Rb1#1-O1#1	67.40(5)	O1-Cs1-O2#2	72.29(12)
O4-Ki-O5#3	61.19(4)	O6-K1-O4#1	70.29(4)			O3#4-Cs1-O3#5	85.48(12)
O4-K1-O6#2	73.35(4)	O6-K1-O6#2	84.27(4)			Cs1-O3#5-Cs1#6	85.48(12)
O4-K1-O4#1	132.96(4)	O6-C1-O5#3	150.03(4)			Cs1#1...Cs1…Cs1#6	180°
O4-K1-O6	132.70(4)	O4#3-C1-O4#1	135.768(17)				
O4-K1-O4#3	91.10(4)	O4#3-C1-O5#3	69.66(4)				
O5-K1-O4#3	59.26(4)	O4#3-C1-O6#2	143.32(4)				
O5-K1-O6	66.60(4)	O4#1-C1-O5#3	122.97(4)				
O5-K1-O4#1	130.89(4)	O4#1-K1-O6#2	69.13(4)				
O5-K1-O6#2	84.10(4)	O5#3-K1-O6#2	124.90(4)				

Table S10. Selected bond lengths and M…M distances (Å) for $[M(4hob)]_n$ **5M** series (M=Li, K, Rb, Cs).

**5Li**		**5K**		**5Rb**		**5Cs**	
**Atoms**	**Bond Lengths**	**Atoms**	**Bond Lengths**	**Atoms**	**Bond Lengths**	**Atoms**	**Bond Lengths**
Li1- O1	1.921(7)	K1-O2	2.869.(13)	Rb1-O1	2.844 (14)	Cs1-O1	3.012(3)
Li1-O1#3	1.969(8)	K1-O2#1	2.917(15)	Rb1-O1#1	2.997(14)	Cs1-O1#1	3.166(3)
Li1-O2#5	1.958(8)	K1-O4	2.734(13)	Rb1-O4	3.028(13)	Cs1-O4	3.217(3)
Li1-O2#4	1.962(7)	K1-O5	2.939 (12)	Rb1-O4#6	3.570 (16)	Cs1-O4#6	3.761(3)
Li1…Li1#2	3.086(8)	K1-O6	3.021(13)	Rb1-O2#4	2.947 (12)	Cs1-O2#4	3.079(3)
Li1…Li1#4	3.086(8)	K1-O4#2	2.848(8)	Rb1-O2#5	2.936 (12)	Cs1-O2#5	3.136(3)
Li1…Li1#3	2.700(14)	K1-O5#3	2.799(5)	Rb1-O3#2	2.946 (13)	Cs1-O3#2	3.108(3)
		K1-O5#4	3.172(5)	Rb1-O3#3	3.120(15)	Cs1-O3#3	3.253(3)
		K1…K1#1	3.9243(7)	Rb1…Rb1#1	4.0584(8)	Cs1…Cs1#1	4.3322(9)

		K1…K1#2	3.9243(7)				

Table S11. Selected bond angles (°) for $[M(4hob)]_n$ **5M** series (M=Li, K, Rb, Cs).

**5Li**		**5K**		**5Rb**		**5Cs**	
Li1#1…Li1…Li1#4	109.6(4)	K1#1…K1…K1#2	128.53(2)	Rb1#1…Rb1…Rb1#7	162.109(5)	Cs1#1…Cs1…Cs1#7	168.391(8)
Li1-O1-C1	136.4(3)	O4–K1–O5	79.41(3)	O3#2-Rb1-O3#3	100.26(4)	O3#2-Cs1-O3#3	105.97(9)
Li1#1-O2-C1	116.3(3)	O4–K1–O6	150.87(3)	O4-Rb1-O4#6	82.89(4)	O4-Cs1-O4#6	81.89(4)
Li1-O2#4-Li1#1	103.9(2)	O5–K1–O6	73.92(3)	Rb1-O3#2-Rb1#7	83.93(3)	Cs1-O3#2-Cs1#7	85.82(7)
Li1-O2#5-Li1#4	103.9(2)	O4–K1–O4#2	83.47(7)				
Li1#4-O1#4-C1#4	136.4(3)	O4–K1–O5#3	126.55(17)				
		O4–K1–O5#4	65.96(5)				
		O5–K1–O5#3	88.46(12)				
		O5–K1–O5#4	135.06(11)				
		O5–K1–O4#2	135.79(6)				
		O6–K1–O4#2	124.17(15)				
		O6–K1–O5#3	64.79(2)				
		O6–K1–O5#4	128.46(8)				
		O4#2–K1–O5#3	69.92(19)				
		O4#2–K1–O5#4	69.35913)				
		O5#4–K1–O5#3	134.98(5)				
		K1-O2-K1#2	85.41(4)				
		O2-K1-O6	55.27(3)				
		O2-K1-O4	144.96(4)				
		O2-K1-O4	127.89(9)				

Table S12. Hydrogen bonding Parameters [d/Å and </°] for compounds **1Rb**, **4K**, **4Rb, 4Cs, 5K, 5Cs**

$[Rb(3tpc)(H_2O)]$ (**1Rb**)

D-H…A	d(D-H)	d(H…A)	d(D…A)	<(DHA)
O3#7-H3d#7…O2	0.8701(17)	2.026(17)	2.861(3)	160.46(5)
O3-H3d…O2#7	0.8701(17)	2.026(17)	2.861(3)	160.46(5)

$[K(4hocin)(H_2O)$ (**4K**)

D-H…A	d(D-H)	d(H…A)	d(D…A)	<(DHA)
O5-H5a…O1#3	0.8503(12)	1.908(13)	2.730(18)	162.41(9)
O3-H3d…O2#7	0.8701(17)	2.065(12)	2.861(3)	160.46(5)
O5-H5b…O2#6	0.8499(13)	2.065(12)	2.8636(18)	156.38(9)
O6-H6a…O2#6	0.8500(13)	2.0067(12)	2.7491(18)	145.41(9)

O4-H4b…O2#4	0.856(16)	2.002(17)	2.8539(18)	174(2)
O4-H4a…O2#2	0.853(17)	1.981(17)	2.8331(18)	176(3)

$[Rb\{H(4hocin)_2\}]_{n.}nH_2O$ **(4Rb)**

D-H…A	d(D-H)	d(H…A)	d(D…A)	<(DHA)
O3-H3…O4	0.8400(12)	1.9027(14)	2.7319(18)	168.95(8)
O3#6-H3#6…O4	0.8400(12)	1.9027(14)	2.7319(18)	168.95(8)
O4-H4B…O2#7	0.850(1)	1.8812(10)	2.7139(15)	166.05(3)
O4-H4A…O2#8	0.8506(10)	1.8718(11)	2.7139(15)	170.22(4)

$[Cs(4hocin)(H_2O)]_n$ **(4Cs)**

D-H…A	d(D-H)	d(H…A)	d(D…A)	<(DHA)
O4-H4b…O1#7	0.870(5)	1.923(5)	2.703(7)	148.5(4)
O4#3-H4b#3…O1	0.870(5)	1.923(5)	2.703(7)	148.5(4)

$[K(4hob)(H_2O)_3]_n$ **(5K)**

D-H…A	d(D-H)	d(H…A)	d(D…A)	<(DHA)
O4-H4a…O1#4	0.8754(11)	1.9342(11)	2.7503(16)	154.50(8)
O5-H5a…O1#4	0.8705(11)	1.9513(11)	2.7756(16)	157.51(7)
O5-H5b…O1#1	0.8706(11)	2.1136(11)	2.8718(17)	145.21(7)
O6-H6b…O1#2	0.8697(11)	2.099(11)	2.8776(16)	161.42(7)

$[Cs(4hob)(H_2O)]_n$ **(5Cs)**

D-H…A	d(D-H)	d(H…A)	d(D…A)	<(DHA)
O4-H4B…O1#6	0.850(3)	1.956(3)	2.742(4)	153.2(2)
O4#6-H4B#6…O1	0.850(3)	1.956(3)	2.742(4)	153.2(2)

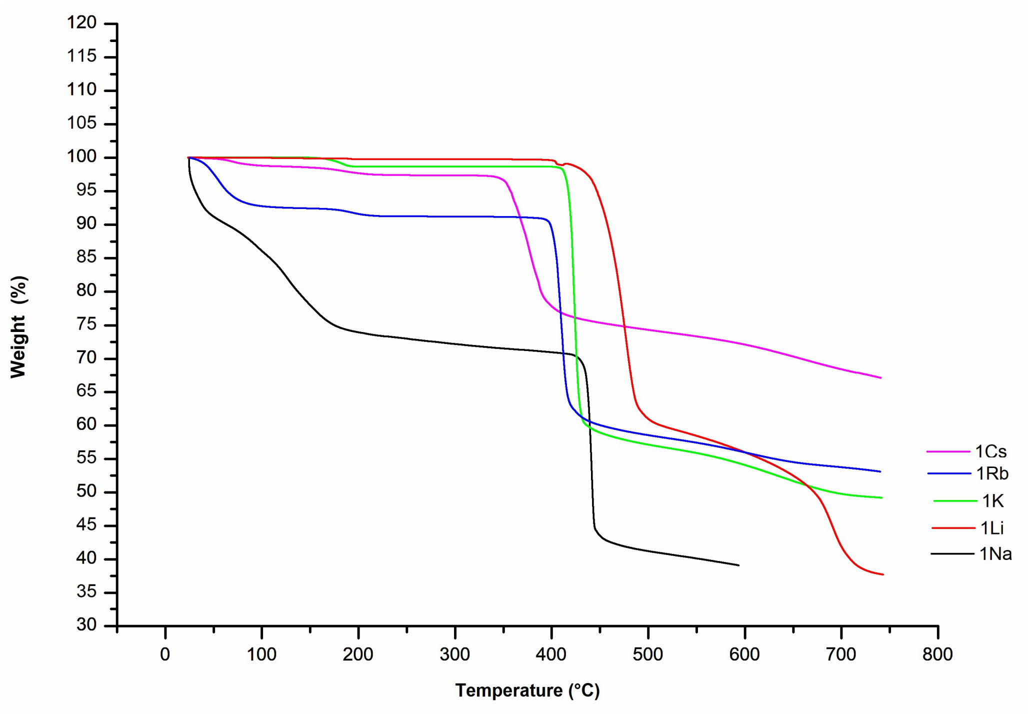

Figure S1. TGA plots of[M(3tpc)]$_n$ **1M** series (M=Na, Li, K, Rb, Cs)

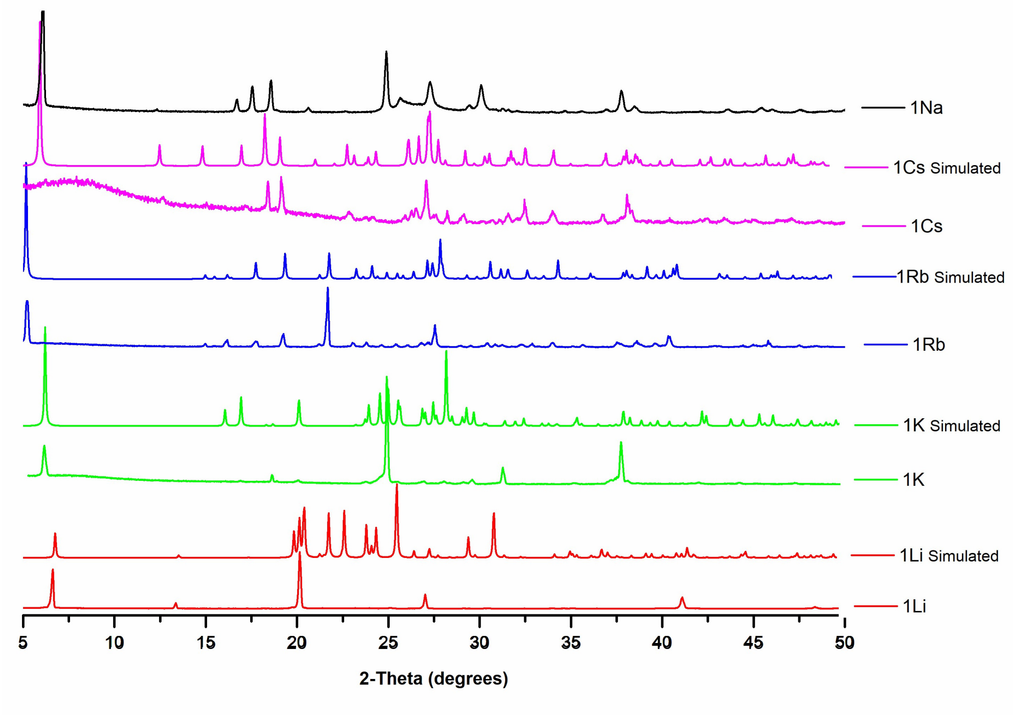

Figure S2. PXRD traces of [M(3tpc)]$_n$ (M=Na, Li, K, Rb, Cs) **1M** series at room temperature compared to their simulated patterns

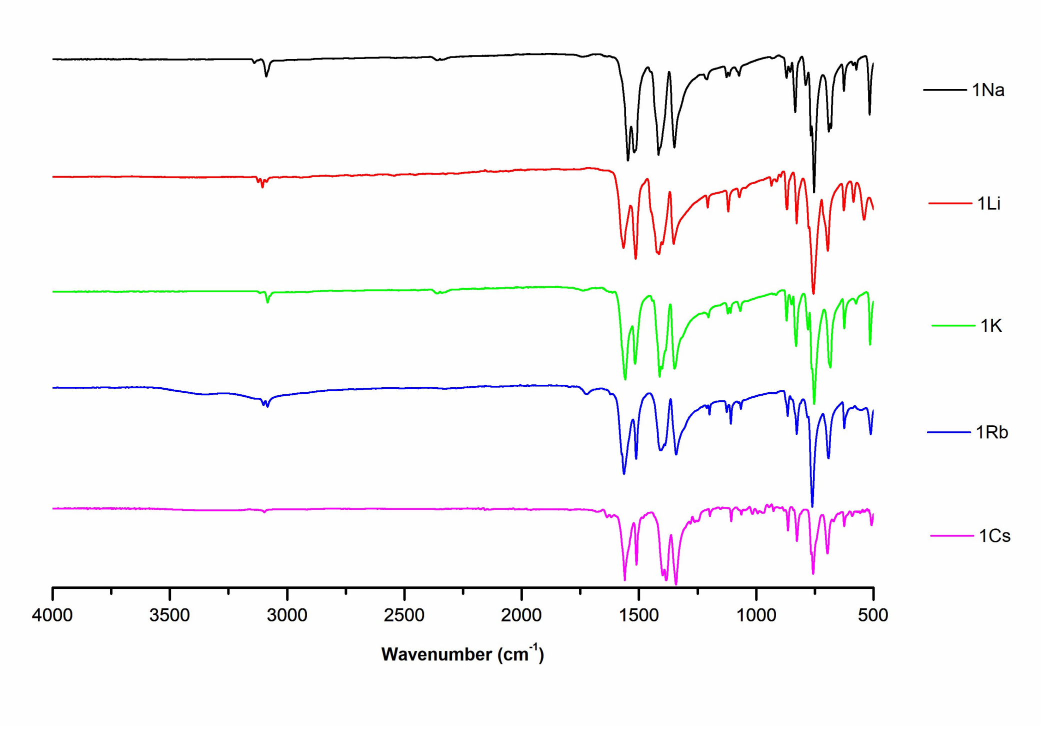

Figure S3. IR spectra of $[M(3tpc)]_n$ **1M** series (M=Na, Li, K, Rb, Cs)

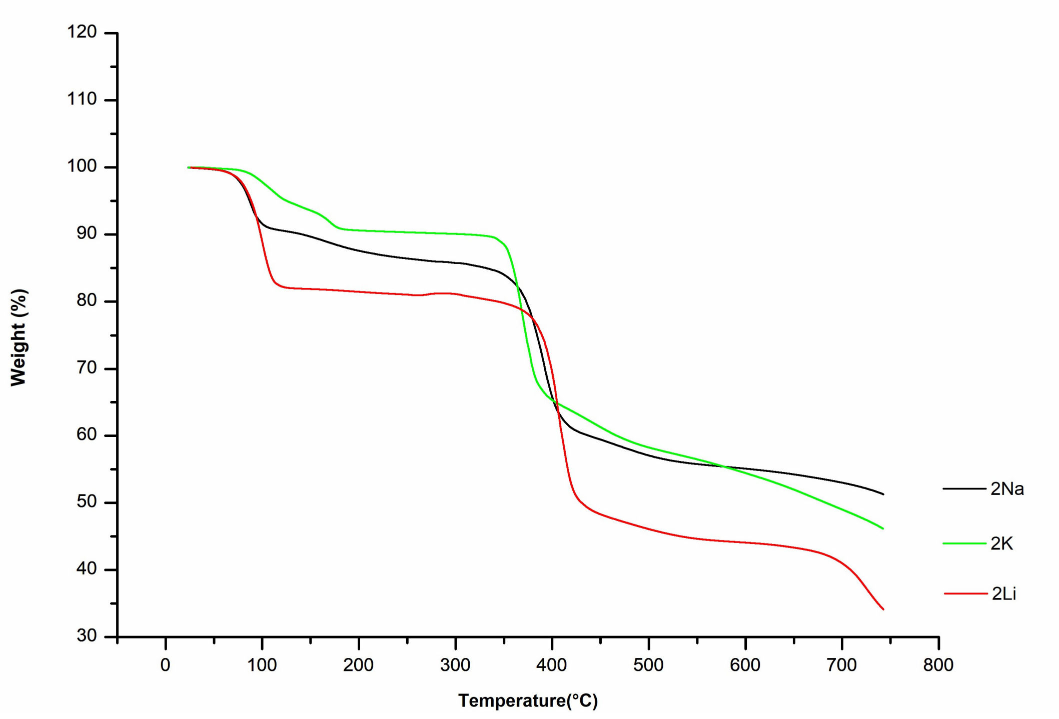

Figure S4. TGA plots of $[M(2m3fur)]_n$ **2M** series (M=Na, Li, K)

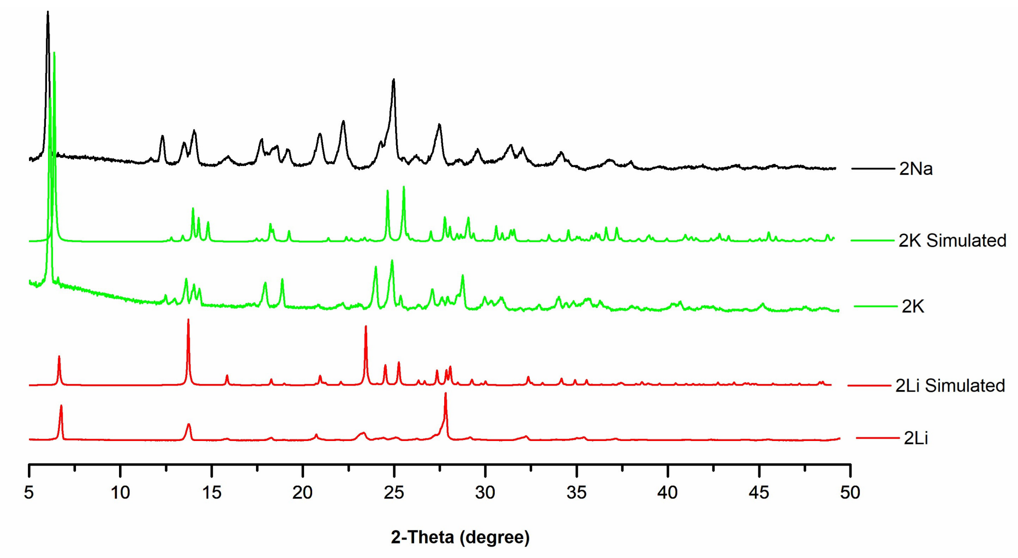

Figure S5. PXRD traces of [M(2m3fur)]$_n$ **2M** series (M=Na, Li, K) series at room temperature compared to their simulated patterns

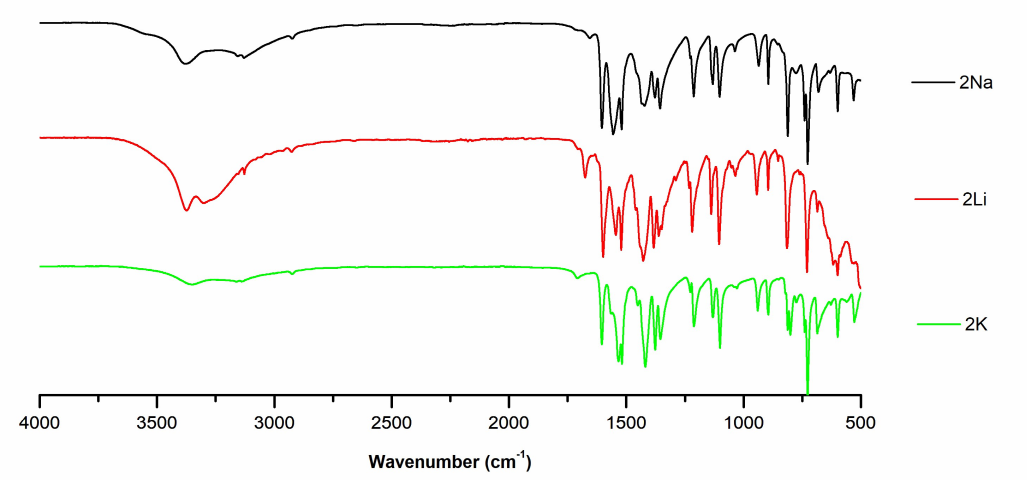

Figure S6. IR spectra of [M(2m3fur)]$_n$ **2M** series (M=Na, Li, K)

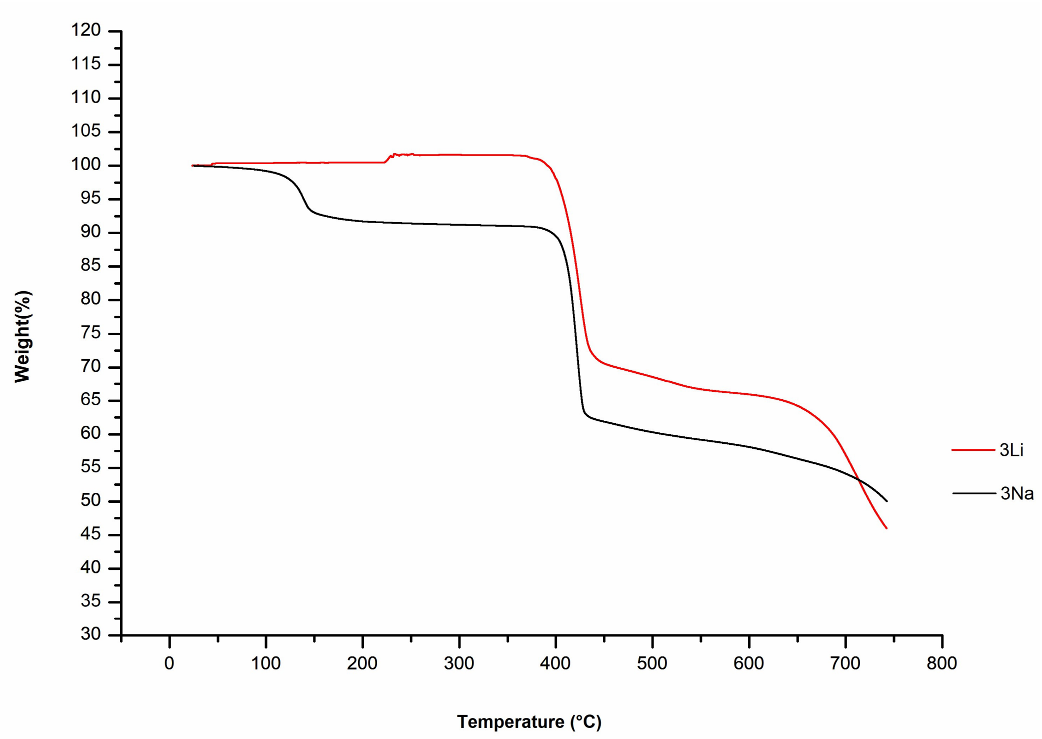

Figure S7. TGA plots of [Na3fur] and [Li(3fur)]$_n$ **(3Li)**

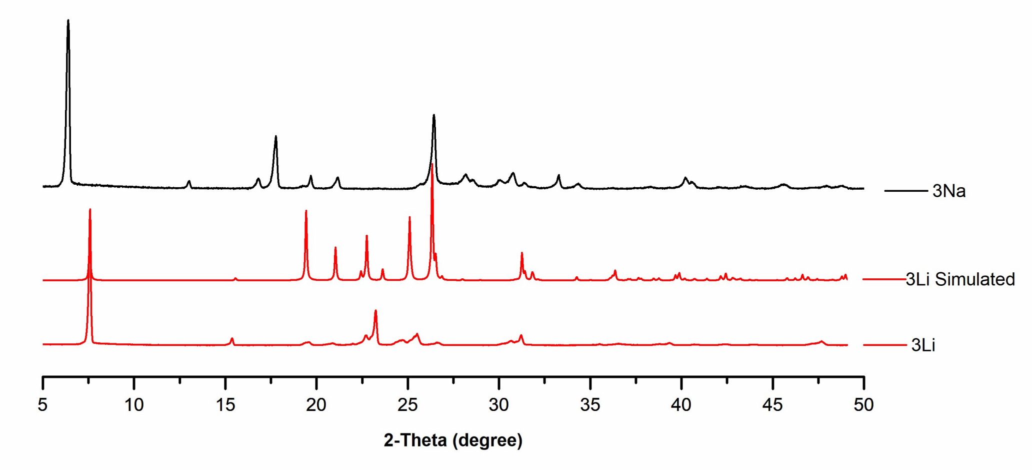

Figure S8. PXRD traces of [Na3fur] and [Li(3fur)]$_n$ **(3Li)** at room temperature compared to the simulated pattern of **3Li**

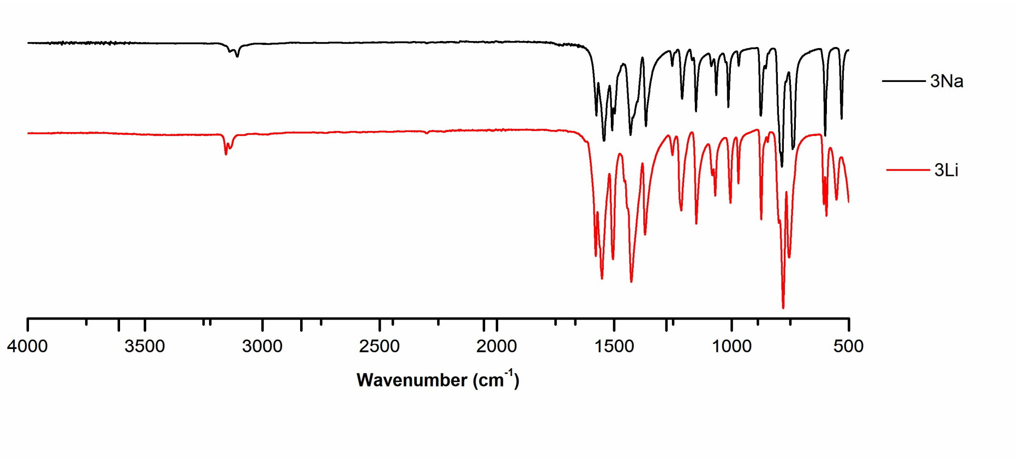

Figure S9. IR spectra of [Na3fur] and $[Li(3fur)]_n$ **(3Li)**

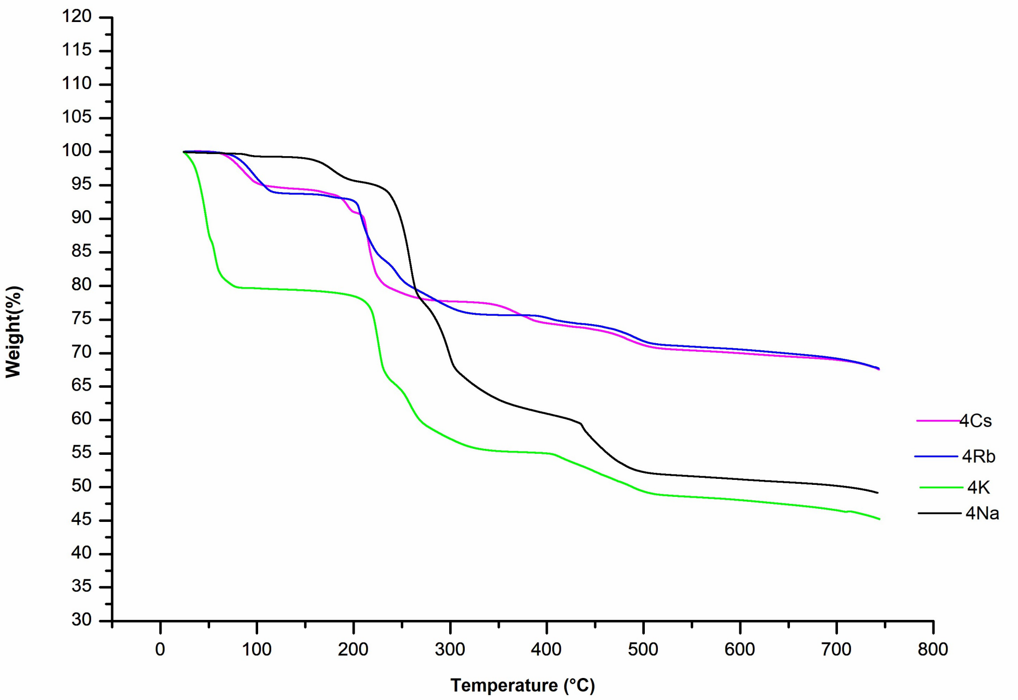

Figure S10. TGA plots of $[M(4hocin)]_n$ **4M** series (M=Na, K, Rb, Cs).

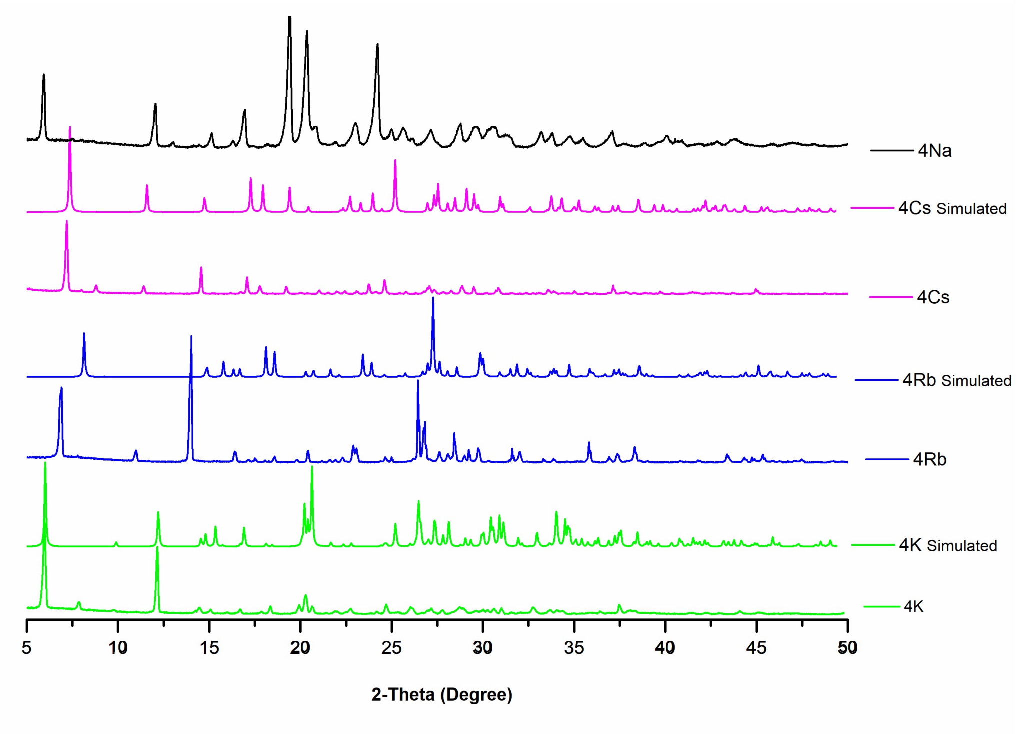

Figure S11. PXRD traces of [M(4hocin)]$_n$ **4M** series (M=Na, K, Rb, Cs) series at room temperature compared to their simulated patterns

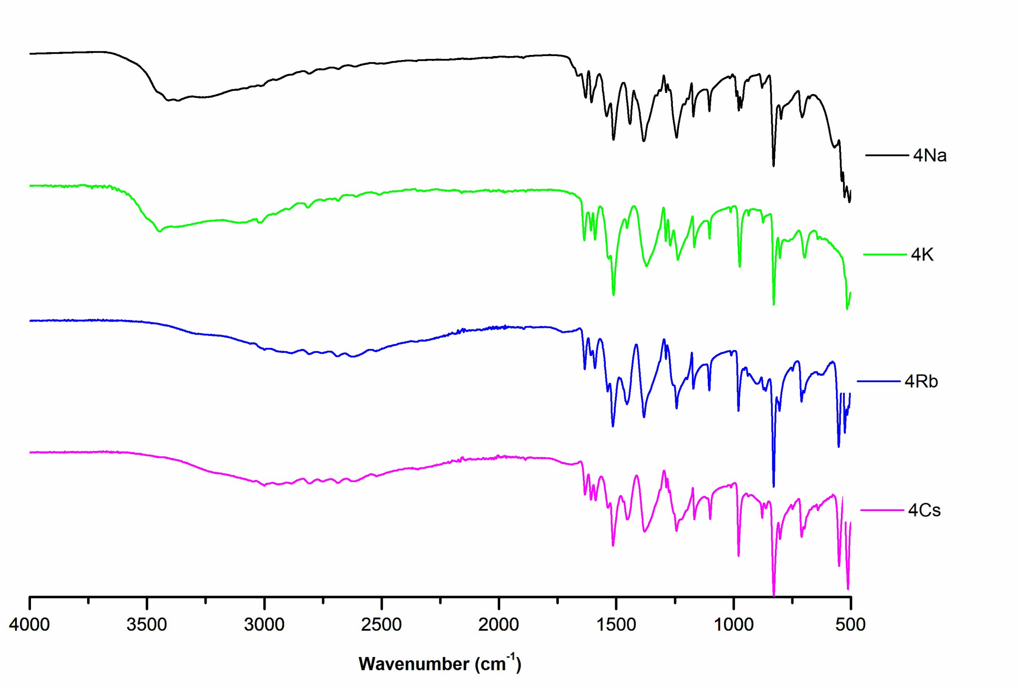

Figure S12. IR spectra of [M(4hocin)]$_n$ **4M** series (M=Na, K, Rb, Cs).

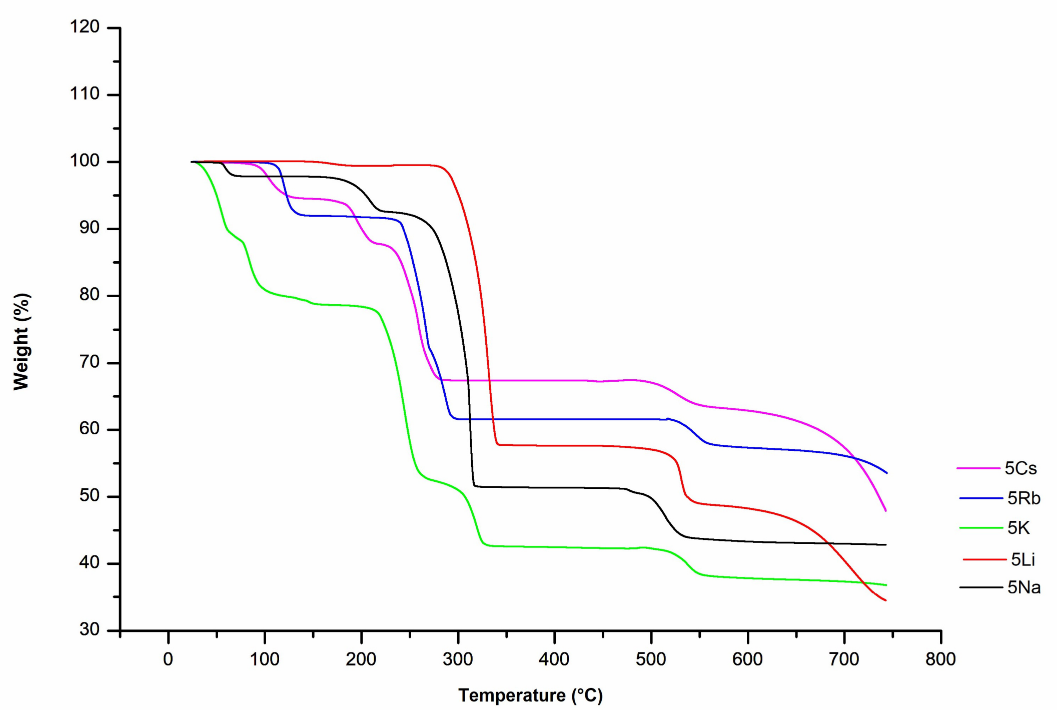

Figure S13. TGA plots of $[M(4hob)]_n$ **5M** series (M=Na, Li, K, Rb, Cs).

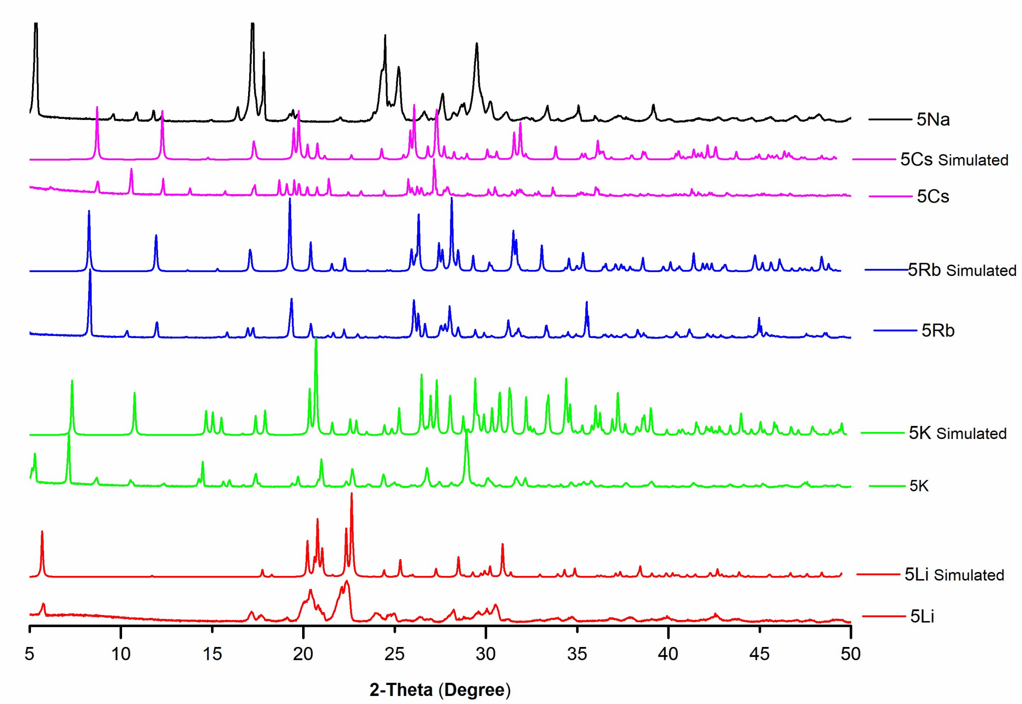

Figure S14. PXRD traces of $[M(4hob)]_n$ **5M** series (M=Na, Li, K, Rb, Cs) series at room temperature compared to their simulated patterns

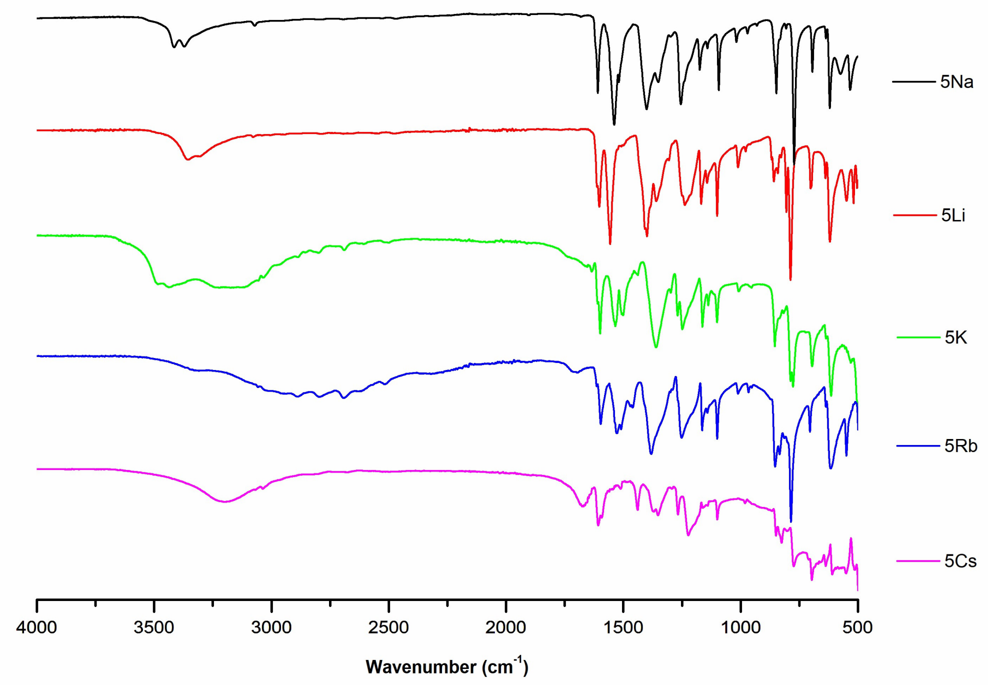

Figure S15. IR spectra of $[M(4hob)]_n$ **5M** series (M=Na, Li, K, Rb, Cs).

	**Before cleaning** the corrosion product	**After Cleaning** the corrosion product
**Control 0.01 M NaCl**	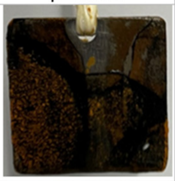	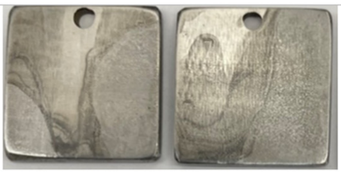
$[Li_2(3tpc)_2]_n$ **(1Li)**	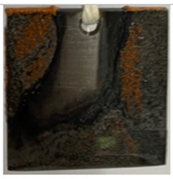	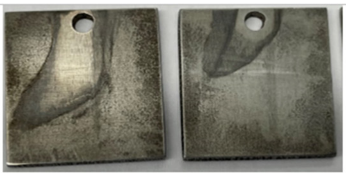
$[K_2(3tpc)_2]_n$ **(1K)**	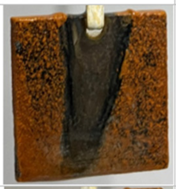	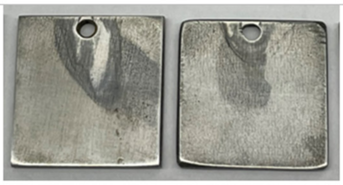
$[Rb(3tpc)(H_2O)]_n$ **(1Rb)**	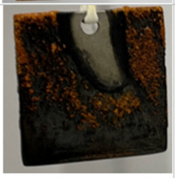	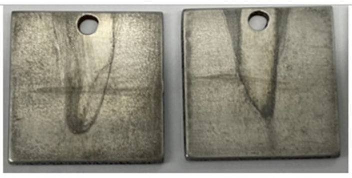
$[K_2(2m3fur)_2(H_2O)]_n$ **(2K)**	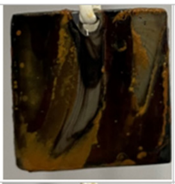	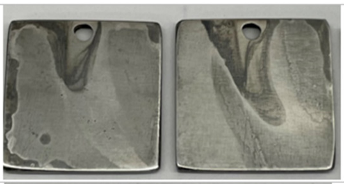
$[Li(3fur)]_n$ **(3Li)**	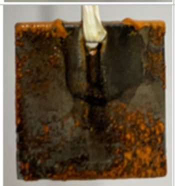	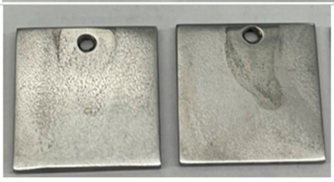
$[K(4hocin)(H_2O)]_3]_n$ **(4K)**	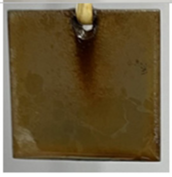	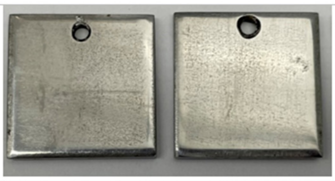
$[K(4HoB)(H_2O)_3]_n$ **(5K)**	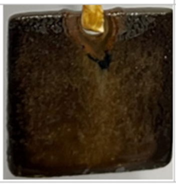	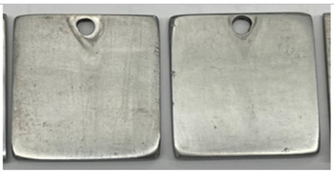

Figure S16. Mild steel coupons immersed in control and the inhibited solutions for 168 h (Left – Trial 1; right – Trial 2.)
